# Crystal structures and conformational features of new forms of tinidazole

**DOI:** 10.1107/S2056989025010126

**Published:** 2025-11-21

**Authors:** Valeryia Hushcha, Justyna Dominikowska, Lilianna Chęcińska

**Affiliations:** a University of Lodz Doctoral School of Exact and Natural Sciences Narutowicza 68 90-136 Łódź Poland; b University of Lodz Faculty of Chemistry Pomorska 163/165 90-236 Łódź Poland; Katholieke Universiteit Leuven, Belgium

**Keywords:** tinidazole, crystal structure, polymorph, inter­action energy, conformers

## Abstract

The crystal structures of two new tinidazole {1-[2-(ethane­sulfon­yl)eth­yl]-2-methyl-5-nitro-1*H*-imidazole, TNZ, C_8_H_13_N_3_O_4_S} forms, triclinic and hemihydrate, have been determined and compared to that of the known monoclinic form. The triclinic and hemihydrate structures each contain two independent mol­ecules with similar conformations, whereas the monoclinic form adopts a distinct geometry. The conformational differences arise mainly from variations in the N—C—C—S torsion angle. A conformational study confirmed two preferred types of mol­ecular conformations characteristic of tinidazole polymorphs. Inter­action-energy analysis indicates that, despite differences in the crystal packing of the monoclinic and triclinic polymorphs, dispersion forces play a major role in consolidation of their structures.

## Abstract 2

Single-crystal X-ray diffraction studies were performed on two new forms of tinidazole: triclinic and hemihydrate, and their structures were compared to that of the known monoclinic form.

## 1. Chemical context

The introduction of nitro­heterocyclic drugs in the late 1950s and the 1960s marked a new era in the treatment of infections caused by both Gram-negative and Gram-positive bacteria, as well as a range of pathogenic protozoan parasites. Azomycin, a 2-nitro­imidazole anti­biotic isolated from a *Streptomyces* bacteria, was the first active nitro­imidazole to be discovered (Nakamura *et al.*, 1955 ▸). It served as the primary impetus for the systematic search for drugs with activity against anaerobic protozoa. Research into alternative 5-nitro­imidazoles began shortly after the introduction of metronidazole, aiming to develop compounds with similar efficacy but improved properties, such as enhanced compliance, longer serum half-life, and better safety profiles.

Tinidazole, 1-(2-ethyl­sulfonyl­eth­yl)-2-methyl-5-nitro-imid­azole (TNZ), synthesized in 1969, has been widely used across Europe and developing countries for the treatment of parasites, mycobacteria, and Gram-positive and Gram-negative bacteria (Ang *et al.*, 2017 ▸). It was approved by the United States Food and Drug Administration (U.S. FDA) in 2004 for the treatment of trichomoniasis, giardiasis, amebiasis, and amoebic liver abscess (Sawyer *et al.*, 1976 ▸; Fung & Doan, 2005 ▸). Tinidazole has emerged as the most successful among these alternative 5-nitro­imidazoles and demonstrates superiority over metronidazole in several respects. It shares a similar anti­microbial spectrum, has a longer half-life, and is better tolerated by patients (Wood & Monro, 1975 ▸; Crowell *et al.*, 2003 ▸; Fung & Doan, 2005 ▸). Most importantly, tinidazole can be effective in overcoming metronidazole resistance in many cases (Gardner & Hill, 2001 ▸).

In this study, the crystal structures of three forms of tinidazole: monoclinic, triclinic and hemihydrate, are described and compared, with emphasis on the mol­ecular conformations and the inter­molecular inter­actions that govern the packing and stability of each polymorph.
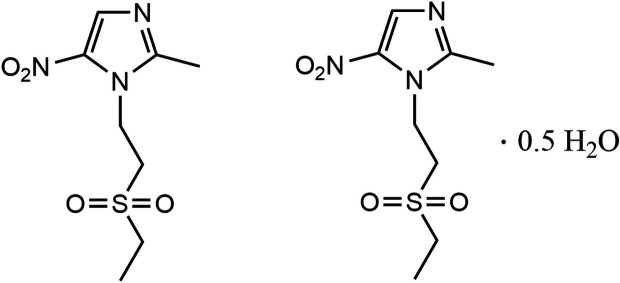


## 2. Structural commentary, conformational analysis and database survey

**Figure 1:**
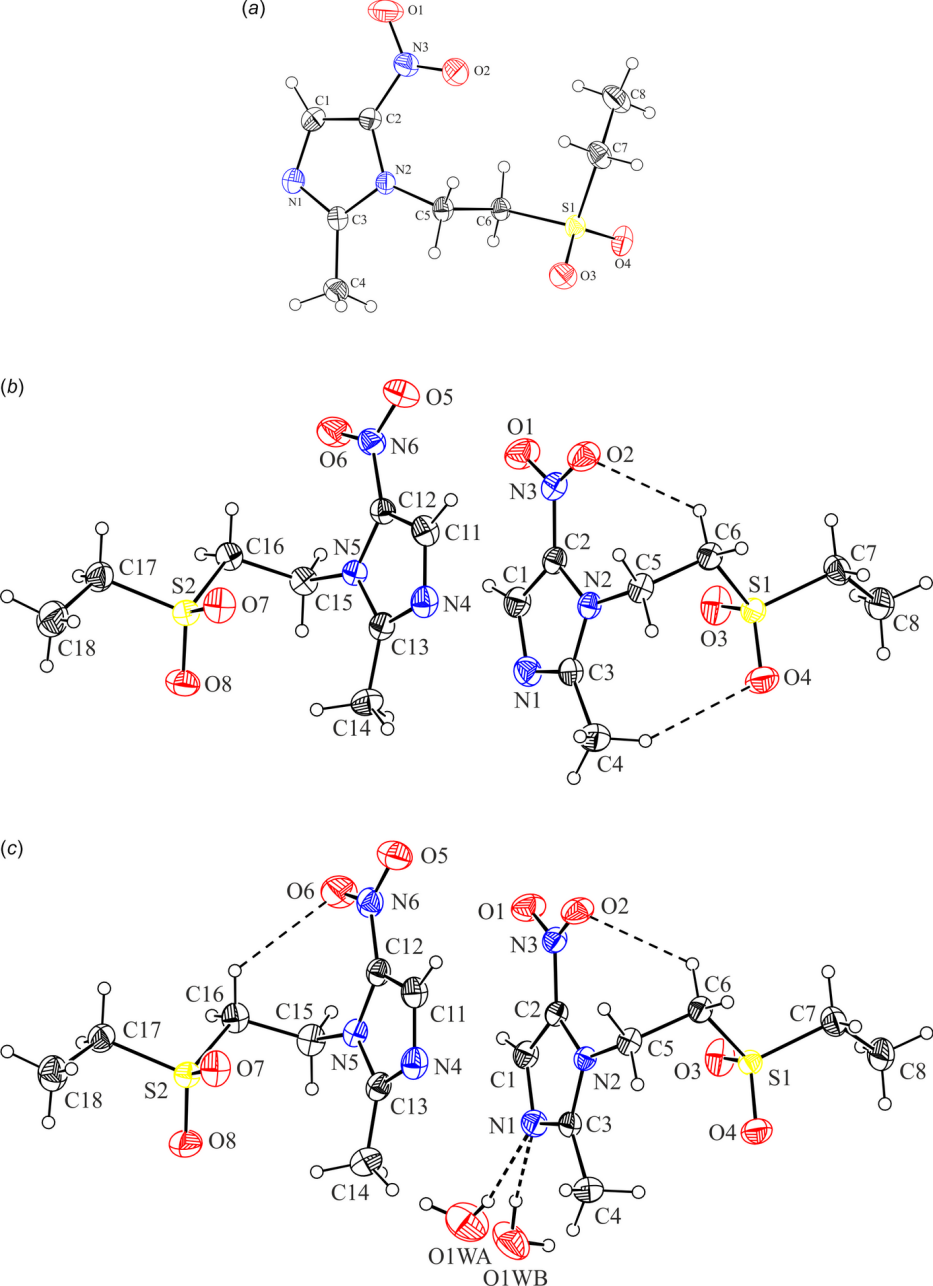
Views of the asymmetric unit of (*a*) TNZ-monoclinic, (*b*) TNZ-triclinic and (*c*) TNZ-hemihydrate, with the atom-numbering schemes. Displacement ellipsoids are drawn at the 30% probability level. H atoms are shown as spheres of arbitrary radii. The disorder components (*A* and *B*) of the water mol­ecule have equal site occupancies (1/2).

**Figure 2:**
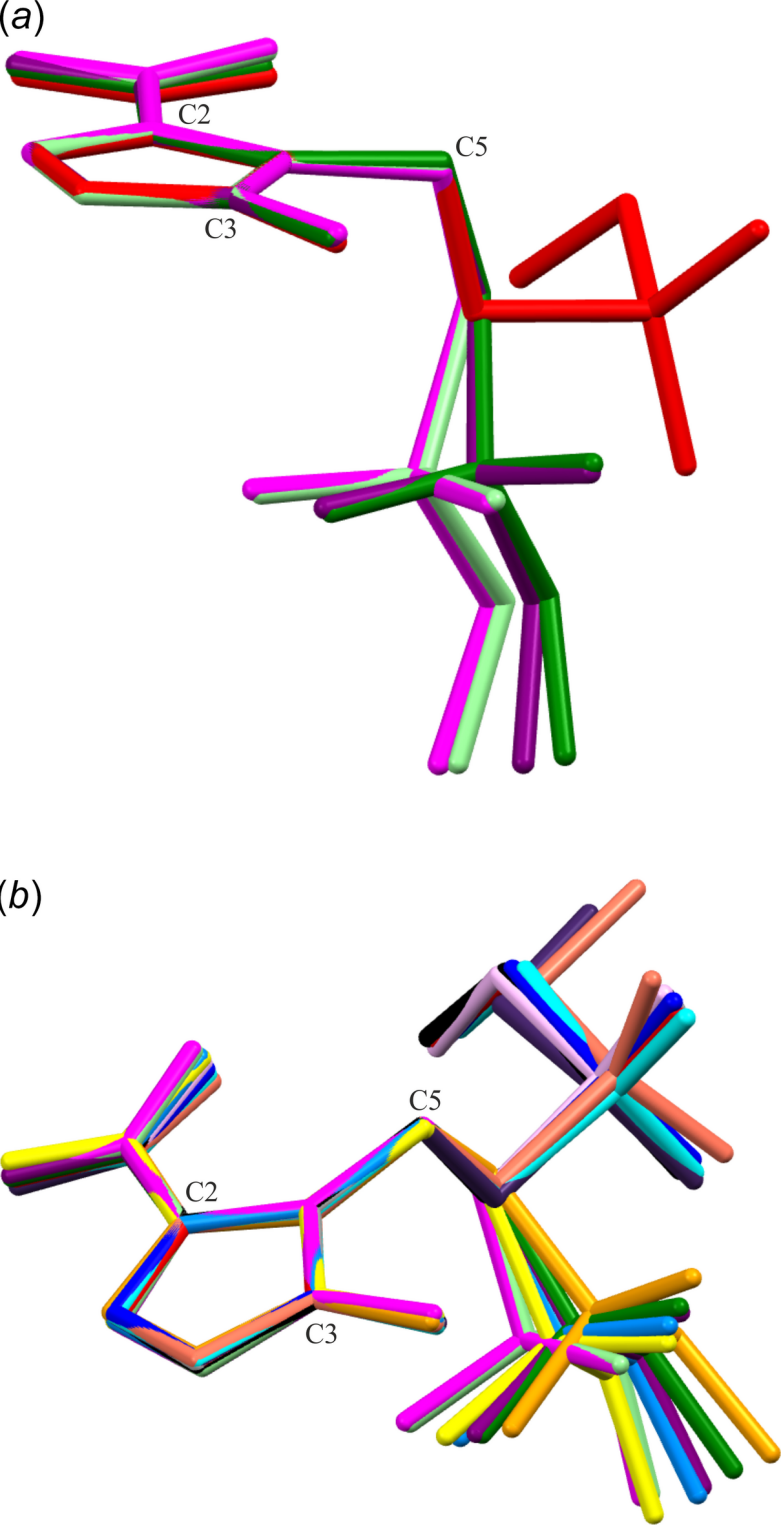
An overlay of (*a*) five tinidazole mol­ecules, with colour codes: red – TNZ-monoclinic, light green – TNZ-triclinic (mol­ecule 1), green – TNZ-triclinic (mol­ecule 2), magenta – TNZ-hemihydrate (mol­ecule 1), purple – TNZ-hemihydrate (mol­ecule 2); and (*b*) additional ten tinidazole mol­ecules from the CSD, with colour codes: CEPSIZ (Chasseaud *et al.*, 1984 ▸) – dark grey, CIPSIZ01 (Zheng *et al.*, 2020 ▸) – black, FISLE (Alfaro-Fuentesa *et al.*, 2014 ▸) – orange, MUKXIC (Fandino *et al.*, 2020 ▸) – cyan, MUKXOI (Fandino *et al.*, 2020 ▸) – blue, NIJCES (Li *et al.*, 2023 ▸) – yellow, NIJCIW (Li *et al.*, 2023 ▸) – pink, NIJCOC (Li *et al.*, 2023 ▸) – navy blue, PUZDEW (mol­ecule 1) (Zheng *et al.*, 2020 ▸) – dark purple, PUZDEW (mol­ecule 2) (Zheng *et al.*, 2020 ▸) – salmon. Atoms C2, C3 and C5 have been used for the overlay.

Fig. 1 ▸*a*–*c* presents the mol­ecular structures of the three pure forms of tinidazole: the monoclinic, triclinic, and hemihydrate forms. In the triclinic and hemihydrate structures, two independent mol­ecules are present, which exhibit highly similar mol­ecular conformations (Fig. 2 ▸*a*). An overlay of all five independent tinidazole mol­ecules shows that the monoclinic form significantly differs from the others.

A search of the Cambridge Structural Database (CSD version 6.00, April 2025; Groom *et al.*, 2016 ▸) revealed eight other additional tinidazole mol­ecules adopting conformations similar to the two observed here (Fig. 2 ▸*b*). Structural analysis of the imidazole valence angle (C—N—C) indicated that only two of these mol­ecules are protonated, with an angle of approximately 109°, whereas the remaining forms are neutral, with the C—N—C angle ranging from 105.6° for TNZ-monoclinic (CEPSIZ; Chasseaud *et al.*, 1984 ▸) to 107.0° for NIJCOC (Li *et al.* 2023 ▸) (Table 1 ▸). The two conformational types can be distinguished by the torsion angle N2—C5—C6—S1/N5—C15—C16—S2, which is approximately ±55–70° in the triclinic and the hemihydrate forms, and ±170° in the monoclinic polymorph. One may say that in the triclinic and the hemihydrate forms, TNZ adopts a conformation close to *gauche* rotamers, whilst the monoclinic polymorph contains TNZ rotamers being close to the anti­periplanar conformation.

In this study, we performed a detailed conformational analysis of the tinidazole mol­ecule based on the experimental crystal structures of its monoclinic and triclinic forms. For this purpose, we determined the crystal structure of the monoclinic form, although it had previously been reported by Chasseaud *et al.* (1984 ▸) and by Zheng *et al.* (2020 ▸).

**Figure 3:**
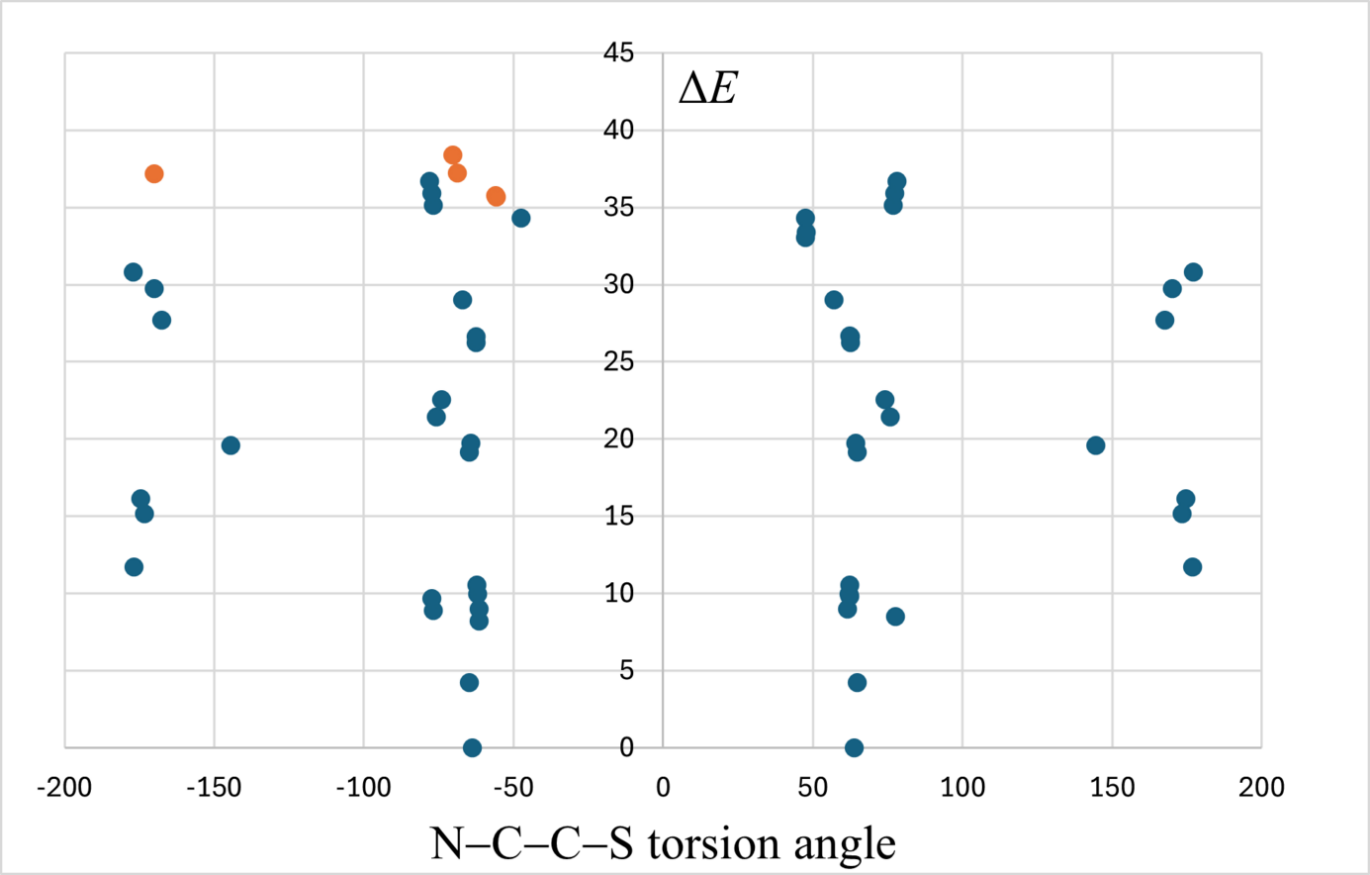
Relative energy values (Δ*E* in kJ mol^−1^) plotted against the N—C—C—S angle (°). Conformers of TNZ generated in the conformational analysis are shown in navy blue; conformers present in the crystal structures of TNZ-monoclinic, TNZ-triclinic and TNZ-hemihydrate in orange.

The geometry optimization at the DFT theory level performed for the generated possible conformers yielded 54 conformers, approximately half of which were found to be unique after evaluation of their relative energies and the sign of the key torsion angle of N—C—C—S. In Fig. 3 ▸ one may notice that, similar to the results of the CSD survey, the obtained conformers can be classified into two groups, namely the one in which the torsion angle N—C—C—S adopts values about ±60° (40 conformers), being close to *gauche* rotamers, and the group in which the values of the N—C—C—S angle are about ±170° (14 conformers), being close to the anti­periplanar rotamer. The energy values for the studied conformers are given in Table S1 in the supporting information. The relative energy values, Δ*E*, for studied conformers range up to 36.7 kJ mol^−1^ and they do not differ significantly for the two main groups of rotamers described above. One may also analyse relative energy values for TNZ in its three forms found in the crystal structure, namely in the monoclinic, triclinic and hemihydrate forms (Table S2 in the supporting information). In this case relative energies are smaller than 2.8 kJ mol^−1^, indicating that the conformers of TNZ present in the crystal structure hardly differ in energy.

## 3. Supra­molecular features

In this study, we compare the supra­molecular architectures of two polymorphs of tinidazole: TNZ-monoclinic and TNZ-triclinic, at room temperature. The analysis is based on inter­action energies calculated using the pairwise model implemented in *CrystalExplorer* (Spackman *et al.*, 2021 ▸). Pairwise model energies (Turner *et al.*, 2014 ▸) were estimated and visualized (Turner *et al.*, 2015 ▸; Mackenzie *et al.*, 2017 ▸) for mol­ecular pairs within a cluster of a radius of 3.8 Å, using a B3LYP/6-31G(d,p) mol­ecular wave function. The total inter­action energy between nearest-neighbour mol­ecular pairs was decomposed into four components: electrostatic, polarization, dispersion and exchange-repulsion with scale factors of 1.057, 0.740, 0,871 and 0.618, respectively.

The crystal structure of the monoclinic form of tinidazole was previously reported by Zheng *et al.* (2020 ▸), including inter­action energy analysis at 100 K. The agreement between the inter­action energies obtained for the room- and low-temperature models is very good, differing by only a few kJ mol^−1^, which can be attributed solely to geometric variations. Nevertheless, this analysis was repeated here to enable direct comparison with the triclinic form, which was found to be unstable at low temperature. The hydrogen-bonding scheme proposed by Zheng *et al.* is very detailed; however in the present work, only the shortest hydrogen bonds with proton⋯acceptor distances shorter by 0.15 Å than the sum of van der Waals radii of the inter­acting atoms were considered. This approach ensures a consistent inter­pretation of the supra­molecular architectures of both polymorphs.

Table 2 ▸ lists selected inter­action energies for mol­ecular pairs connected by hydrogen bonds, as summarized in Tables 3 ▸ and 4 ▸ for TNZ-monoclinic and TNZ-triclinic, respectively. Complete inter­action energy data are provided in Tables S4 and S5 in the supporting information. The pairwise model analysis was not performed for the TNZ-hemihydrate structure because the positional disorder of the water mol­ecule complicated such calculations.

**Figure 4:**
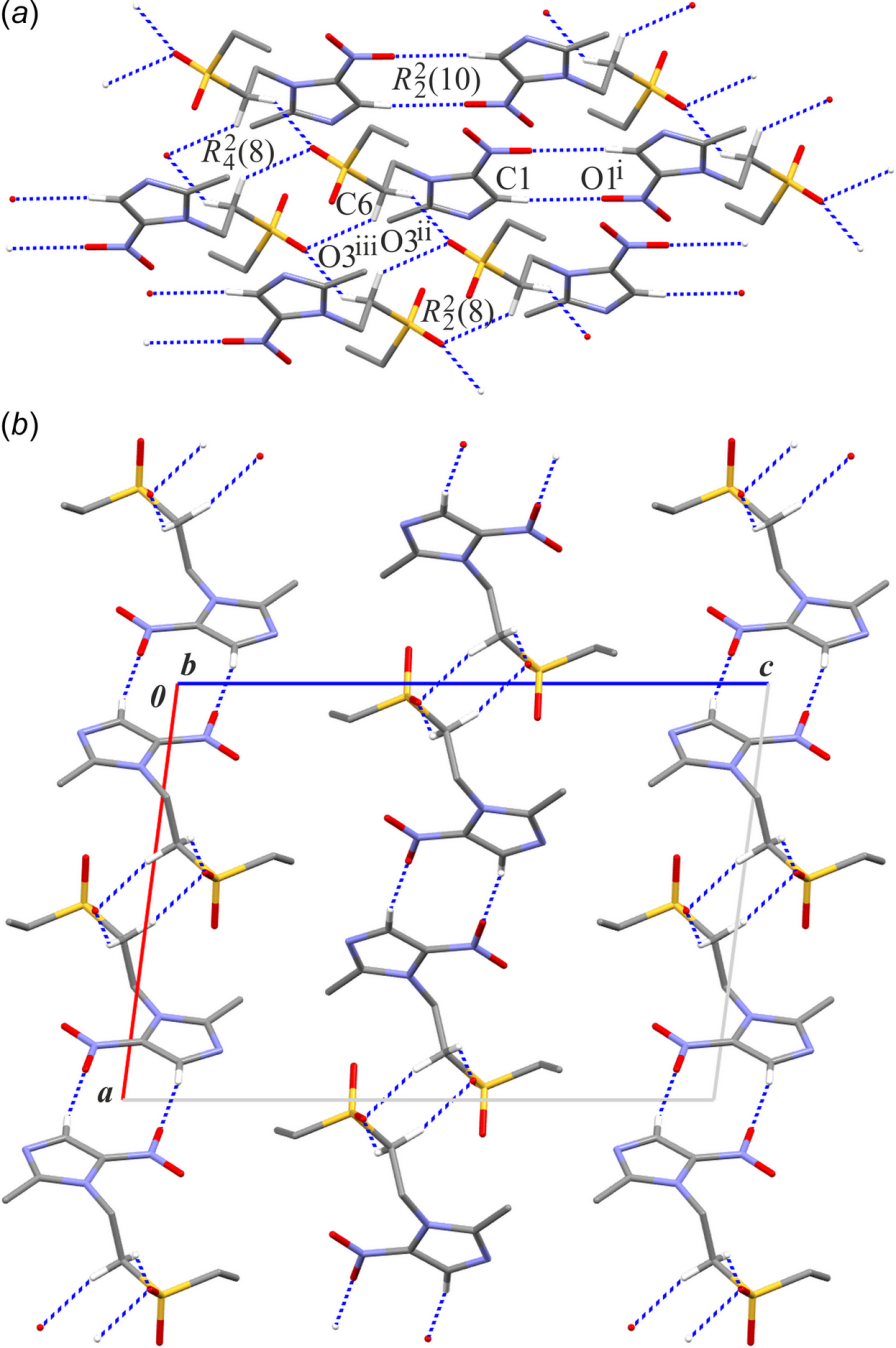
A part of the crystal structure of TNZ-monoclinic showing (*a*) a scheme of inter­actions and (*b*) an arrangement of di-periodic layers in a view along the *b* axis. Hydrogen bonds are drawn as dashed lines and (C)—H atoms not involved in hydrogen bonds have been omitted. Symmetry codes: (i) −*x* + 1, −*y* + 2, −*z* + 1; (ii) *x*, *y* + 1, *z*; (iii) −*x*, −*y*, −*z* + 1.

In the crystal structure of TNZ-monoclinic, three C—H⋯O hydrogen bonds are observed (Table 3 ▸). In two of these inter­actions, atom C6 acts as the donor and atom O3 as an acceptor. The C6—H6*A*⋯O3(*x*, *y* + 1, *z*) inter­action forms a *C*(4) chain motif running along the [010] direction, while the C6—H6*B*⋯O3(−*x*, −*y*, −*z* + 1) inter­action generates an 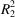
(8) motif (Etter, 1990 ▸; Etter *et al.*, 1990 ▸; Bernstein *et al.*, 1995 ▸). The combination of these two hydrogen bonds results in a finite pattern of 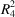
(8) motifs, constructing a chain of edge-fused centrosymmetric rings (Fig. 4 ▸*a*). The total inter­action energies of these two C—H⋯O contacts are −30.6 and −64.6 kJ mol^−1^, respectively. The most linear inter­action, C1—H1⋯O1(−*x* + 1, −*y* + 2, −*z* + 1), is responsible for the formation of centrosymmetric dimers with an 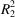
(10) motif (*E*_tot_=–11.9 kJ mol^−1^). This inter­action links the aforementioned chain of rings along the [100] direction. As a result, supra­molecular di-periodic layers are formed, lying parallel to the (001) plane (Fig. 4 ▸*b*). No other direction-specific inter­actions are observed between the layers.

**Figure 5:**
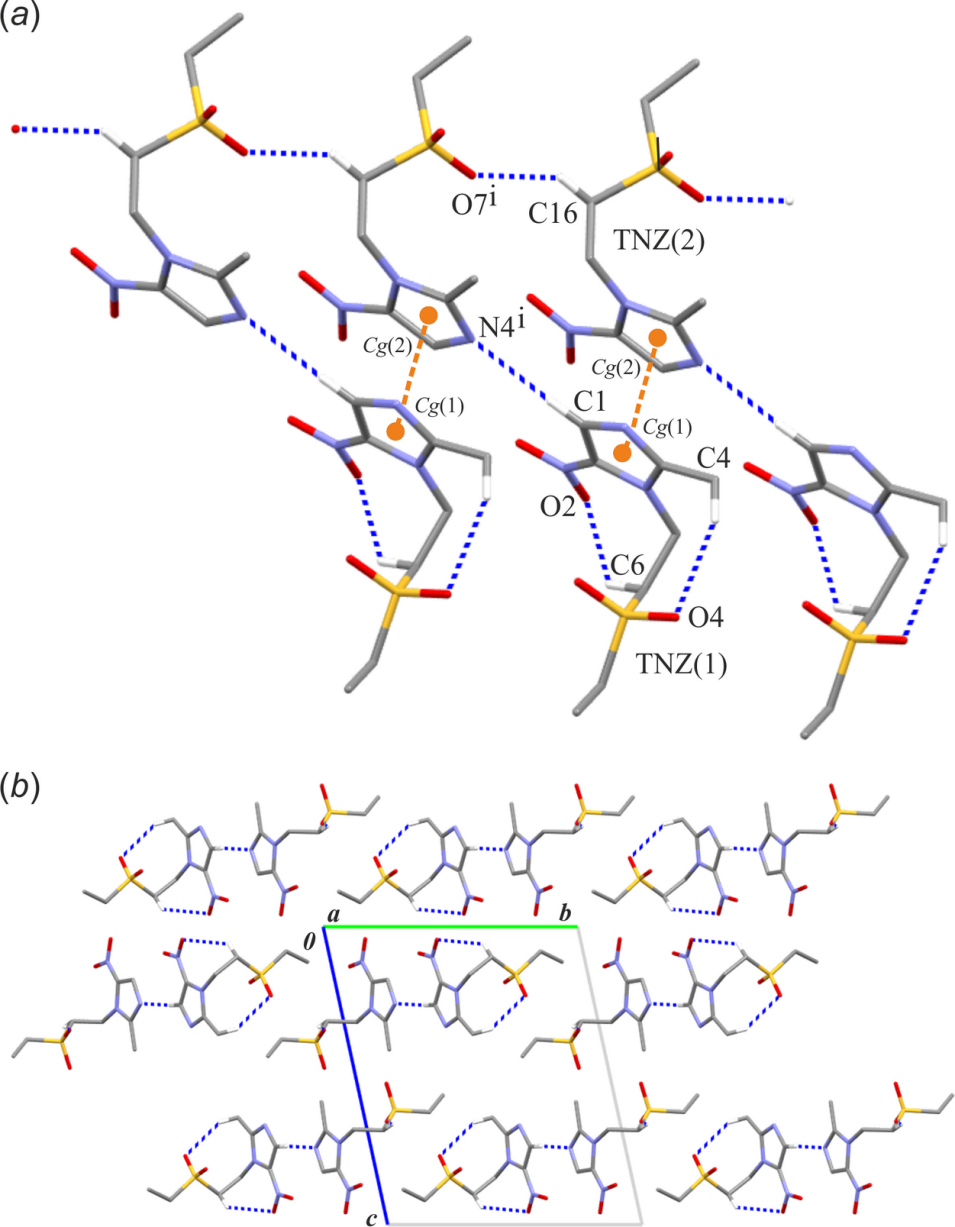
A part of the crystal structure of TNZ-triclinic showing (*a*) a scheme of inter­actions and (*b*) an arrangement of mono-periodic ribbons in a view along the *a* axis. Hydrogen bonds are drawn as blue dashed lines and (C)—H atoms not involved in hydrogen bonds have been omitted. Orange balls correspond to the centre of gravity of the imidazole rings [denoted *Cg*(1) and *Cg*(2)]. Orange dashed lines represent aromatic π–π inter­actions. Symmetry code: (i) *x* + 1, *y*, *z*.

In the crystal structure of TNZ-triclinic, two independent TNZ mol­ecules are present in the asymmetric unit (Fig. 1 ▸*b*). The mol­ecular structure of TNZ(1) mol­ecule features two intra­molecular hydrogen bonds: C4—H4*C*⋯O4 and C6—H6*B*⋯O2 (Table 4 ▸). In addition, there are two inter­molecular hydrogen bonds of the C—H⋯O/N types. The C16—H16*A*⋯O7(*x* + 1, *y*, *z*) inter­action generates a *C*(4) chain motif built from TNZ(2) mol­ecules and running along the [100] direction (*E*_tot_=–28.5 kJ mol^−1^). TNZ(1) mol­ecules bind to this chain *via* the C1—H1⋯N4(*x* + 1, *y*, *z*) inter­action (*E*_tot_=–17.7 kJ mol^−1^), forming finite d-type motifs (Fig. 5 ▸*a*). As a result, supra­molecular mono-periodic ribbons are formed. Within these ribbons, aromatic π–π stacking inter­actions occur between the imidazole rings of the independent tinidazole mol­ecules (1) and (2) from the asymmetric unit (Table S3 in the supporting information). This aromatic inter­action exhibits the total energy of −30.5 kJ mol^−1^, dominated by the highest dispersion contribution of −32.1 kJ mol^−1^. No direction-specific inter­actions are observed between the ribbon assemblies (Fig. 5 ▸*b*).

**Figure 6:**
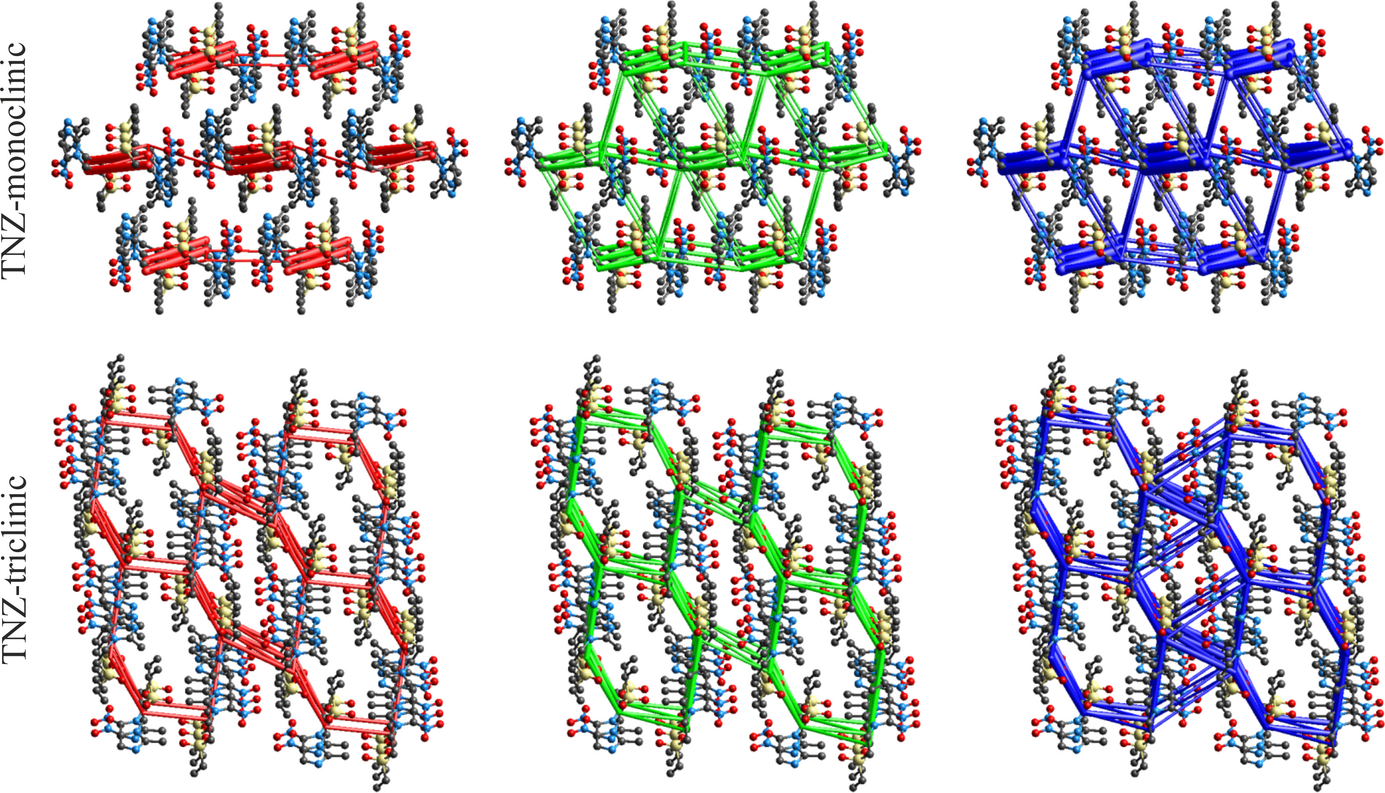
The representative energy framework diagrams for separate electrostatic (red) and dispersion (green) components, and the total inter­action energy (blue) for TNZ-monoclinic (viewed along the *b* axis) and TNZ-triclinic (viewed along the *a* axis). All cylindrical radii are proportional to the relative strength of the corresponding energies and they were adjusted to the same scale factor of 80 with a cut-off value of −10 kJ mol^−1^.

In summary, comparison of the two polymorphs of tinidazole from an energetic perspective shows that the highest total inter­action energies occur in the monoclinic form (up to approximately −60 kJ mol^−1^), compared with less than −40 kJ mol^−1^ in the triclinic form, although both structures feature only C—H⋯O/N hydrogen bonds. Inter­estingly, in both polymorphs, the ratio of electrostatic to dispersion energy contributions summed over mol­ecular pairs within the 3.8 Å cluster is the same, at approximately 40:60 (2:3). To put this result into perspective, in two isavuconazole polymorphs, the electrostatic-to-dispersive contribution ratio differs: being 25:75 for the monoclinic and 42:58 for the ortho­rhom­bic form, respectively (Ben & Chęcińska, 2025 ▸). The ratio 2:3 indicates that both supra­molecular architectures are strongly influenced by non-directional dispersive inter­actions. This trend is reflected in the energetic frameworks, where the tri-periodic pattern of the total inter­action energies corresponds with that found for the dispersion component (Fig. 6 ▸). The variety in the electrostatic component distribution is smaller for TNZ-triclinic, with its tri-periodic motif resembling that of the dispersion distribution, than in TNZ-monoclinic, in which the electrostatic distribution is essentially mono-periodic, being dominated by a single strong directional inter­action.

**Figure 7:**
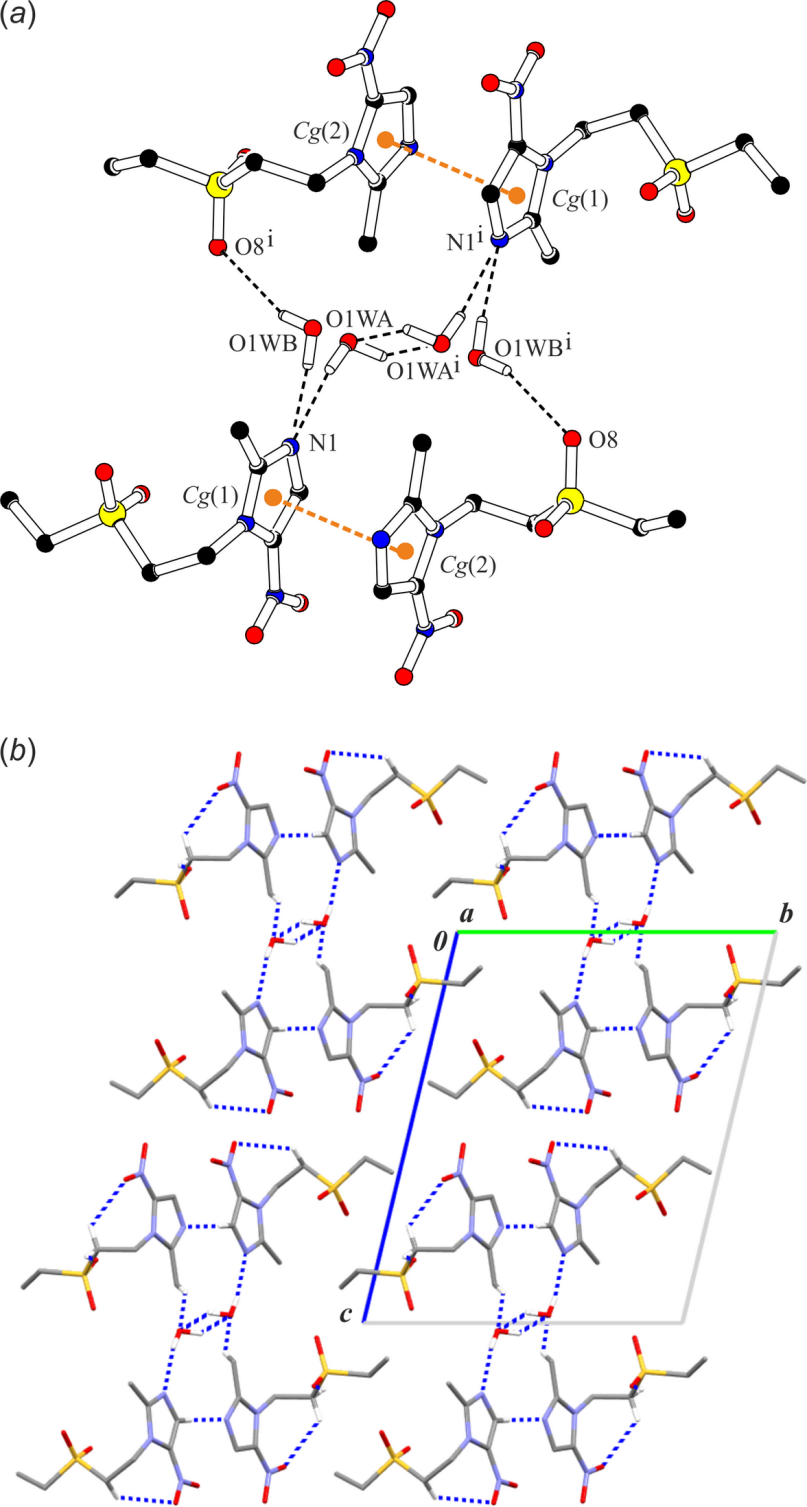
A part of the crystal structure of TNZ-hemihydrate showing (*a*) a scheme of hydrogen bonds to disordered *A* and *B* components of the water mol­ecule and (*b*) a scheme of mono-periodic column-like assemblies in a view along the a axis (for clarity, disorder component *B* of the water mol­ecule has been omitted). Hydrogen bonds are drawn as black dashed lines and (C)—H atoms not involved in hydrogen bonds have been omitted. Orange balls correspond to the centre of gravity of the imidazole rings [denoted *Cg*(1) and *Cg*(2)]. Orange dashed lines represent aromatic π-π- inter­actions. Symmetry code: (i) −*x* + 1, −*y* + 1, −*z*.

In the crystal structure of TNZ-hemihydrate, each independent TNZ mol­ecule features one short intra­molecular hydrogen bond: C6—H6*B*⋯O2 in TNZ(1) and C16—H16*B*⋯O6 in TNZ(2), respectively (Fig. 1 ▸*c*; Table 5 ▸). As in TNZ-triclinic, supra­molecular ribbons are formed through the C16—H16*A*⋯O7(*x* + 1, *y*, *z*) and C1—H1⋯N4(*x* + 1, *y*, *z*) inter­actions. Both components of the disordered water mol­ecule participate in hydrogen bonding with tinidazole mol­ecules *via* O1*WA*—H1*WB*⋯N1 (component *A*), and O1*WB*—H1*WC*⋯N1 and O1*WB*—H1*WD*⋯O8(−*x* + 1, −*y* + 1, −*z*) (component *B*) inter­actions. Two additional C—H⋯O inter­actions: C14—H14*B*⋯O1*WA*(−*x* + 1, −*y* + 1, −*z*) and C15—H15*A*⋯O1*WB*(−*x* + 1, −*y* + 1, −*z*) further stabilize the mol­ecular substructure (Fig. 7 ▸*a*). Finally, the ribbons doubled across the inversion centre form column-like assemblies (Fig. 7 ▸*b*). The aromatic π–π-inter­action, *Cg*(1)⋯*Cg*(2), is preserved within the ribbon structure (Table S3 in the supporting information).

## 4. Hirshfeld surface analysis

**Figure 8:**
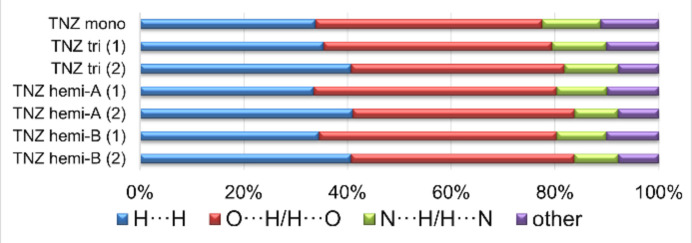
Diagram of percentage contributions of different close contacts to the Hirshfeld surface area of TNZ mol­ecules in three analysed forms, including tinidazole mol­ecules (1) and (2) and two positions of the disordered water mol­ecule (*A* and *B*).

Hirshfeld surface analysis (Spackman & McKinnon, 2002 ▸; Spackman & Jayatilaka, 2009 ▸) was performed using *CrystalExplorer* (Spackman *et al.*, 2021 ▸) to visualize and qu­antify inter­molecular inter­actions in all three (solvato)polymorphs of tinidazole. As shown in the breakdown diagram (Fig. 8 ▸), the major contributions to the Hirshfeld surface in the described three forms arise from H⋯H and O⋯H/H⋯O contacts. These two types of inter­actions complement each other and together sum up approximately to 80% of the surface contributions (Figs. S1 and S2 in the supporting information). In the hemihydrate solvatomorph, the trend observed for the two independent mol­ecules, TNZ(1) and TNZ(2), in the triclinic form is retained, with the proportion of O⋯H/H⋯O contacts increasing by only about 2%. In all three analysed structures, N⋯H/H⋯N contacts represent the third most significant contribution to the Hirshfeld surface of the TNZ mol­ecules, amounting to roughly 10%. In the hemihydrate form, for TNZ(1), some of these contacts are shorter (represented by the longer spikes in Fig. S2 in the supporting information) than in the other forms, due to hydrogen bonding between TNZ(1) and both components of the disordered water mol­ecule.

## 5. Synthesis and crystallization

The tinidazole (purity > 98%) used in this study was purchased from Angene Chemical (India), 4-nitro­benzoic acid (purity > 99%) was purchased from Sigma-Aldrich (USA).

All three forms of tinidazole were obtained during the attempted cocrystallization of the drug with 4-nitro­benzoic acid. For cocrystal synthesis, equimolar qu­anti­ties (0.05 mmol of each) of tinidazole and 4-nitro­benzoic acid were ground together using a mortar and pestle. The resulting fine powder was then dissolved in ethanol and heated to 345 K. The solution was filtered and covered with perforated paraffin film. Finally, it was left to evaporate slowly at room temperature until crystals formed. Although cocrystals of tinidazole and 4-nitro­benzoic acid were not obtained, two new forms of tinidazole were identified: TNZ-hemihydrate and TNZ-triclinic, along with the known monoclinic form.

## 6. Refinement

Crystal data, data collection and structure refinement details are summarized in Table 6 ▸. All (C)—H atoms were placed geometrically and refined as a riding model with *U*_iso_(H) = 1.2*U*_eq_(C) for the methyl­ene and aromatic groups, and 1.5*U*_eq_(C) for the methyl group. During the refinement of TNZ-hemihydrate, the water mol­ecule was found to be disordered and refined with two alternative positions (0.5 site-occupancy factor for both components). The H atoms on the O atoms were constrained using the command AFIX6, with their *U*_iso_ fixed at 1.5*U*_eq_(O).

## 7. Theoretical calculations

A conformational search for the neutral mol­ecules of tinidazole was performed using *Mercury* (Macrae *et al.*, 2020 ▸), considering all rotatable bonds. The 200 conformations suggested by the program were subsequently subjected to DFT calculations using *GAUSSIAN09* (Frisch *et al.*, 2013 ▸) at the B3LYP-GD3BJ/6-311G(d,p) level of theory (Becke, 1993 ▸; Grimme *et al.*, 2011 ▸; Johnson & Becke, 2006 ▸). The optimized geometries were confirmed as stationary points by the absence of imaginary vibrational frequencies. In some cases, a single low-magnitude imaginary frequency (<11*i* cm^−1^) was observed (Table S1 in the supporting information). Final Cartesian coordinates (X, Y, Z in Å) for the optimized TNZ conformers are listed in Tables S6–S59 in the supporting information.

Single-point energy calculations were also performed using the same level of theory for five independent tinidazole mol­ecules extracted from the crystal structures of TNZ-monoclinic, TNZ-triclinic and TNZ-hemihydrate forms, to put the results of conformational analysis into perspective. Prior to DFT calculations, hydrogen-atom positions were normalized according to the values reported by Allen & Bruno (2010 ▸). Cartesian coordinates (X, Y, Z in Å) for the TNZ-mol­ecules taken from the crystal structures of TNZ-monoclinic, TNZ-triclinic and TNZ-hemihydrate are listed in Tables S60–S64 in the supporting information.

## Supplementary Material

Crystal structure: contains datablock(s) TNZ-monoclinic, TNZ-triclinic, TNZ-hemihydrate, global. DOI: 10.1107/S2056989025010126/vm2319sup1.cif

Structure factors: contains datablock(s) TNZ-monoclinic. DOI: 10.1107/S2056989025010126/vm2319TNZ-monoclinicsup2.hkl

Structure factors: contains datablock(s) TNZ-triclinic. DOI: 10.1107/S2056989025010126/vm2319TNZ-triclinicsup3.hkl

Structure factors: contains datablock(s) TNZ-hemihydrate. DOI: 10.1107/S2056989025010126/vm2319TNZ-hemihydratesup4.hkl

Supporting information file. DOI: 10.1107/S2056989025010126/vm2319TNZ-monoclinicsup5.cml

Supporting information file. DOI: 10.1107/S2056989025010126/vm2319TNZ-triclinicsup6.cml

Supporting information file. DOI: 10.1107/S2056989025010126/vm2319TNZ-hemihydratesup7.cml

Additional tables and figures for conformational study, Hirshfeld surface and energy framework analyses. DOI: 10.1107/S2056989025010126/vm2319sup8.docx

CCDC references: 2502468, 2502467, 2502466

Additional supporting information:  crystallographic information; 3D view; checkCIF report

## Figures and Tables

**Table 1:** Selected geometric parameters (Å, °) for TNZ mol­ecules TNZ(1) and TNZ(2) – independent TNZ mol­ecules from the asymmetric unit.

Structure/Refcode	Study temp. (K)	C3—N1—C1/C13—N4—C11	C2—N2—C5—C6/C12—N5—C15—C16	C3—N2—C5—C6/C13—N5—C15—C16	N2—C5—C6—S1/N5—C15—C16—S2
TNZ-triclinic(1)	294	105.66 (17)	–64.0 (2)	109.6 (2)	–56.1 (2)
TNZ-triclinic(2)	294	106.46 (17)	–69.7 (2)	107.0 (2)	–70.36 (19)
TNZ-hemihydrate(1)	294	106.6 (3)	–63.1 (4)	109.5 (4)	–55.9 (4)
TNZ-hemihydrate(2)	294	106.8 (3)	–69.6 (5)	106.1 (4)	–68.8 (4)
TNZ-monoclinic	294	105.83 (12)	–77.93 (16)	97.29 (15)	170.06 (8)
CEPSIZ (TNZ-monoclinic)	295	105.6	–77.5	97.3	170.1
CEPSIZ01 (TNZ-monoclinic)	100	105.9	75.9	–98.3	–169.5
FISLIE	293	109.9	75.3	–108.4	75.7
MUKXIC	173	106.6	81.5	–97.6	–173.0
MUKXOI	173	107.0	67.6	–105.9	66.9
NIJCES	293	109.8	–69.5	104.0	–61.7
NIJCIW	293	106.6	78.9	–98.9	–167.0
NIJCOC	293	107.0	–80.6	99.9	169.8
PUZDEW	100	106.6	–82.4	100.7	173.9
PUZDEW	100	106.2	81.5	–94.6	–165.6

**Table 2:** Inter­action energies (kJ mol^−1^) for selected mol­ecular pairs TNZ(1) and TNZ(2) – independent TNZ mol­ecules from the asymmetric unit. *N* is the number of mol­ecular pairs. *R* is the distance (Å) between mol­ecular centroids. *E*_tot_ is the total energy and its individual components: *E*_ele_ is electrostatic (*k* = 1.057), *E*_pol_ is polarization (*k* = 0.740), *E*_dis_ is dispersion (*k* = 0.871), *E*_rep_ is repulsion (*k* = 0.618).

Structure	Mol­ecular pair	Inter­action	*kE*_ele_	*kE*_pol_	*kE*_dis_	*kE*_rep_	*kE*_tot_
TNZ-monoclinic	TNZ–TNZ	C1—H1⋯O1^i^	–12.8	–1.3	–6.8	9.0	–11.9
	TNZ–TNZ	C6—H6*A*⋯O3^ii^	–11.2	–4.0	–27.5	12.0	–30.6
	TNZ–TNZ	C6—H6*B*⋯O3^iii^	–47.2	–8.7	–37.4	18.8	–64.6
TNZ-triclinic	TNZ(1)–TNZ(1)	C1—H1⋯N4^i^	–20.4	–3.3	–9.5	15.5	–17.7
	TNZ(2)–TNZ(2)	C16—H16*A*⋯O7^i^	–17.3	–4.0	–21.8	14.6	–28.5
	TNZ(1)–TNZ(2)	*Cg*(1)⋯*Cg*(2)	–7.5	–1.9	–32.1	11.0	–30.5

Symmetry codes: TNZ-monoclinic (i) −*x* + 1, −*y* + 2, −*z* + 1; (ii) *x*, *y* + 1, *z*; (iii) −*x*, −*y*, −*z* + 1. TNZ-triclinic: (i) *x* + 1, *y*, *z*.

**Table 3:** Hydrogen-bond geometry (Å, °) for TNZ-monoclinic[Chem scheme1]

*D*—H⋯*A*	*D*—H	H⋯*A*	*D*⋯*A*	*D*—H⋯*A*
C1—H1⋯O1^i^	0.93	2.45	3.374 (2)	171
C6—H6*A*⋯O3^ii^	0.97	2.53	3.3777 (17)	146
C6—H6*B*⋯O3^iii^	0.97	2.53	3.2942 (17)	136

Symmetry codes: (i) 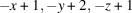; (ii) 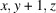; (iii) 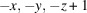.

**Table 4:** Hydrogen-bond geometry (Å, °) for TNZ-triclinic[Chem scheme1]

*D*—H⋯*A*	*D*—H	H⋯*A*	*D*⋯*A*	*D*—H⋯*A*
C1—H1⋯N4^i^	0.93	2.39	3.319 (3)	174
C4—H4*C*⋯O4	0.96	2.51	3.365 (3)	148
C6—H6*B*⋯O2	0.97	2.41	3.057 (3)	124
C16—H16*A*⋯O7^i^	0.97	2.34	3.177 (2)	145

Symmetry code: (i) 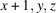.

**Table 5:** Hydrogen-bond geometry (Å, °) for TNZ-hemihydrate[Chem scheme1]

*D*—H⋯*A*	*D*—H	H⋯*A*	*D*⋯*A*	*D*—H⋯*A*
O1*WA*—H1*WB*⋯N1	0.85	2.09	2.908 (5)	162
O1*WB*—H1*WC*⋯N1	0.85	2.06	2.882 (5)	163
O1*WA*—H1*WA*⋯O1WA^i^	0.85	1.44	2.180 (10)	143
O1*WB*—H1*WD*⋯O8^i^	0.85	2.56	3.370 (5)	158
C1—H1⋯N4^ii^	0.93	2.38	3.310 (3)	175
C6—H6*B*⋯O2	0.97	2.42	3.063 (2)	124
C14—H14*B*⋯O1*WA*^i^	0.96	2.49	3.348 (6)	149
C15—H15*A*⋯O1*WB*^i^	0.97	2.46	3.41 (4)	168
C16—H16*B*⋯O6	0.97	2.50	3.100 (3)	120
C16—H16*A*⋯O7^ii^	0.97	2.37	3.183 (2)	141

Symmetry codes: (i) 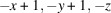; (ii) 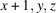.

**Table 6:** Experimental details

	TNZ-monoclinic	TNZ-triclinic	TNZ-hemihydrate
Crystal data			
Chemical formula	C_8_H_13_N_3_O_4_S	C_8_H_13_N_3_O_4_S	C_8_H_13_N_3_O_4_S·0.5H_2_O
*M*_r_	247.27	247.27	256.28
Crystal system, space group	Monoclinic, *P*2_1_/*n*	Triclinic, *P* 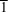	Triclinic, *P* 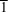
Temperature (K)	294	294	294
*a*, *b*, *c* (Å)	11.9943 (2), 5.5233 (1), 16.8454 (2)	5.7208 (2), 13.2759 (5), 15.5932 (5)	5.7223 (1), 13.1854 (2), 16.2439 (4)
α, β, γ (°)	90, 97.499 (1), 90	77.101 (3), 85.407 (2), 77.923 (3)	102.658 (2), 92.226 (2), 102.484 (2)
*V* (Å^3^)	1106.43 (3)	1128.16 (7)	1162.76 (4)
*Z*	4	4	4
Radiation type	Cu *K*α	Cu *K*α	Cu *K*α
μ (mm^−1^)	2.69	2.64	2.61
Crystal size (mm)	0.27 × 0.06 × 0.04	0.23 × 0.04 × 0.02	0.22 × 0.06 × 0.03

Data collection			
Diffractometer	Rigaku XtaLAB Synergy, Dualflex, HyPix	Rigaku XtaLAB Synergy, Dualflex, HyPix	Rigaku XtaLAB Synergy, Dualflex, HyPix
Absorption correction	Gaussian (*CrysAlis PRO* 1.171.42.88a; Rigaku OD, 2023 ▸)	Gaussian (*CrysAlis PRO* 1.171.42.88a; Rigaku OD, 2023 ▸)	Gaussian (*CrysAlis PRO* 1.171.44.109a; Rigaku OD, 2025 ▸)
*T*_min_, *T*_max_	0.208, 1.000	0.496, 1.000	0.372, 1.000
No. of measured, independent and observed [*I* > 2σ(*I*)] reflections	10724, 2118, 2011	11280, 4260, 3758	11604, 4366, 3775
*R*_int_	0.022	0.032	0.029
(sin θ/λ)_max_ (Å^−1^)	0.617	0.617	0.617

Refinement			
*R*[*F*^2^ > 2σ(*F*^2^)], *wR*(*F*^2^), *S*	0.028, 0.081, 1.08	0.039, 0.103, 1.03	0.037, 0.101, 1.06
No. of reflections	2118	4260	4366
No. of parameters	148	293	311
H-atom treatment	H-atom parameters constrained	H-atom parameters constrained	H-atom parameters constrained
Δρ_max_, Δρ_min_ (e Å^−3^)	0.22, −0.22	0.20, −0.34	0.22, −0.34

Computer programs: *CrysAlis PRO* 1.171.42.88a and 1.171.44.109a (Rigaku OD, 2023 ▸, 2025 ▸), *SHELXT* (Sheldrick, 2015*a* ▸), *SHELXL2019/2* and *SHELXL2019/3* (Sheldrick, 2015*b* ▸), *Mercury* (Macrae *et al.*, 2020 ▸); *PLATON* (Spek, 2020 ▸) and *publCIF* (Westrip, 2010 ▸).

## supplementary crystallographic information

### 1-[2-(Ethanesulfonyl)ethyl]-2-methyl-5-nitro-1H-imidazole (TNZ-monoclinic) Crystal data

**Table:**

C_8_H_13_N_3_O_4_S	*F*(000) = 520
*M**_r_* = 247.27	*D*_x_ = 1.484 Mg m^−^^3^
Monoclinic, *P*2_1_/*n*	Cu *K*α radiation, λ = 1.54184 Å
*a* = 11.9943 (2) Å	Cell parameters from 8802 reflections
*b* = 5.5233 (1) Å	θ = 3.7–76.7°
*c* = 16.8454 (2) Å	µ = 2.69 mm^−^^1^
β = 97.499 (1)°	*T* = 294 K
*V* = 1106.43 (3) Å^3^	Prism, colourless
*Z* = 4	0.27 × 0.06 × 0.04 mm

### 1-[2-(Ethanesulfonyl)ethyl]-2-methyl-5-nitro-1H-imidazole (TNZ-monoclinic) Data collection

**Table:**

Rigaku XtaLAB Synergy, Dualflex, HyPix diffractometer	2118 independent reflections
Radiation source: micro-focus sealed X-ray tube, PhotonJet (Cu) X-ray Source	2011 reflections with *I* > 2σ(*I*)
Mirror monochromator	*R*_int_ = 0.022
Detector resolution: 10.0000 pixels mm^-1^	θ_max_ = 72.1°, θ_min_ = 4.3°
ω scans	*h* = −14→14
Absorption correction: gaussian (CrysAlisPro 1.171.42.88a; Rigaku OD, 2023)	*k* = −6→5
*T*_min_ = 0.208, *T*_max_ = 1.000	*l* = −20→20
10724 measured reflections	

### 1-[2-(Ethanesulfonyl)ethyl]-2-methyl-5-nitro-1H-imidazole (TNZ-monoclinic) Refinement

**Table:**

Refinement on *F*^2^	Hydrogen site location: inferred from neighbouring sites
Least-squares matrix: full	H-atom parameters constrained
*R*[*F*^2^ > 2σ(*F*^2^)] = 0.028	*w* = 1/[σ^2^(*F*_o_^2^) + (0.0448*P*)^2^ + 0.258*P*] where *P* = (*F*_o_^2^ + 2*F*_c_^2^)/3
*wR*(*F*^2^) = 0.081	(Δ/σ)_max_ < 0.001
*S* = 1.08	Δρ_max_ = 0.22 e Å^−^^3^
2118 reflections	Δρ_min_ = −0.22 e Å^−^^3^
148 parameters	Extinction correction: SHELXL-2019/2 (Sheldrick 2015b), Fc^*^=kFc[1+0.001xFc^2^λ^3^/sin(2θ)]^-1/4^
0 restraints	Extinction coefficient: 0.0032 (4)
Primary atom site location: structure-invariant direct methods	

### 1-[2-(Ethanesulfonyl)ethyl]-2-methyl-5-nitro-1H-imidazole (TNZ-monoclinic) Special details

**Table:**

Geometry. All esds (except the esd in the dihedral angle between two l.s. planes) are estimated using the full covariance matrix. The cell esds are taken into account individually in the estimation of esds in distances, angles and torsion angles; correlations between esds in cell parameters are only used when they are defined by crystal symmetry. An approximate (isotropic) treatment of cell esds is used for estimating esds involving l.s. planes.

### 1-[2-(Ethanesulfonyl)ethyl]-2-methyl-5-nitro-1H-imidazole (TNZ-monoclinic) Fractional atomic coordinates and isotropic or equivalent isotropic displacement parameters (Å^2^)

**Table:**

	*x*	*y*	*z*	*U*_iso_*/*U*_eq_	
S1	0.03059 (3)	0.16011 (6)	0.39070 (2)	0.03666 (14)	
O1	0.42652 (13)	0.8226 (2)	0.43028 (8)	0.0750 (4)	
O2	0.32312 (11)	0.5198 (3)	0.38502 (7)	0.0742 (4)	
O3	0.04594 (10)	−0.09214 (19)	0.41130 (6)	0.0530 (3)	
O4	−0.08151 (8)	0.2575 (2)	0.38353 (6)	0.0528 (3)	
N1	0.38083 (10)	0.5737 (2)	0.65305 (7)	0.0446 (3)	
N2	0.29935 (9)	0.3847 (2)	0.54357 (6)	0.0339 (2)	
N3	0.36965 (11)	0.6432 (3)	0.44040 (8)	0.0490 (3)	
C1	0.40961 (12)	0.6894 (3)	0.58822 (9)	0.0456 (3)	
H1	0.456123	0.824475	0.589665	0.055*	
C2	0.36016 (11)	0.5784 (3)	0.52011 (8)	0.0391 (3)	
C3	0.31386 (11)	0.3923 (3)	0.62476 (8)	0.0365 (3)	
C4	0.25958 (14)	0.2226 (3)	0.67572 (9)	0.0480 (4)	
H4A	0.179988	0.251558	0.669057	0.072*	
H4B	0.289754	0.246947	0.730774	0.072*	
H4C	0.273755	0.059203	0.660413	0.072*	
C5	0.22722 (11)	0.2145 (2)	0.49345 (8)	0.0358 (3)	
H5A	0.215666	0.069870	0.523997	0.043*	
H5B	0.263587	0.167511	0.447640	0.043*	
C6	0.11443 (10)	0.3321 (2)	0.46491 (7)	0.0321 (3)	
H6A	0.127442	0.491009	0.443368	0.039*	
H6B	0.073507	0.354111	0.510396	0.039*	
C7	0.08610 (14)	0.2091 (3)	0.29962 (8)	0.0477 (4)	
H7A	0.165436	0.168332	0.307386	0.057*	
H7B	0.048995	0.100205	0.259364	0.057*	
C8	0.07285 (17)	0.4664 (3)	0.26841 (9)	0.0610 (5)	
H8A	0.116133	0.573974	0.305129	0.092*	
H8B	−0.005002	0.511921	0.263308	0.092*	
H8C	0.099019	0.476101	0.217024	0.092*	

### 1-[2-(Ethanesulfonyl)ethyl]-2-methyl-5-nitro-1H-imidazole (TNZ-monoclinic) Atomic displacement parameters (Å^2^)

**Table:**

	*U*^11^	*U*^22^	*U*^33^	*U*^12^	*U*^13^	*U*^23^
S1	0.0409 (2)	0.0338 (2)	0.0333 (2)	−0.00394 (12)	−0.00236 (13)	−0.00431 (11)
O1	0.0962 (10)	0.0667 (9)	0.0654 (8)	−0.0238 (7)	0.0232 (7)	0.0158 (6)
O2	0.0706 (8)	0.1125 (12)	0.0389 (6)	−0.0288 (8)	0.0045 (5)	0.0036 (7)
O3	0.0749 (8)	0.0319 (5)	0.0503 (6)	−0.0078 (5)	0.0012 (5)	−0.0031 (5)
O4	0.0366 (5)	0.0654 (7)	0.0532 (6)	−0.0017 (5)	−0.0059 (4)	−0.0076 (5)
N1	0.0453 (7)	0.0467 (7)	0.0402 (6)	−0.0066 (5)	−0.0003 (5)	−0.0040 (5)
N2	0.0311 (5)	0.0358 (6)	0.0334 (5)	−0.0001 (4)	−0.0006 (4)	−0.0002 (4)
N3	0.0445 (7)	0.0586 (8)	0.0441 (7)	−0.0006 (6)	0.0075 (5)	0.0086 (6)
C1	0.0435 (8)	0.0428 (8)	0.0496 (8)	−0.0086 (6)	0.0031 (6)	−0.0016 (6)
C2	0.0375 (7)	0.0402 (7)	0.0394 (7)	−0.0011 (6)	0.0045 (5)	0.0040 (6)
C3	0.0349 (6)	0.0390 (7)	0.0344 (6)	0.0013 (5)	−0.0003 (5)	−0.0011 (5)
C4	0.0563 (9)	0.0489 (8)	0.0386 (7)	−0.0067 (7)	0.0048 (6)	0.0021 (7)
C5	0.0376 (7)	0.0323 (6)	0.0360 (6)	0.0025 (5)	−0.0008 (5)	−0.0053 (5)
C6	0.0345 (6)	0.0300 (6)	0.0308 (6)	0.0010 (5)	0.0002 (5)	−0.0040 (5)
C7	0.0612 (9)	0.0495 (8)	0.0314 (7)	0.0021 (7)	0.0019 (6)	−0.0068 (6)
C8	0.0859 (13)	0.0590 (11)	0.0367 (8)	−0.0007 (9)	0.0025 (7)	0.0073 (7)

### 1-[2-(Ethanesulfonyl)ethyl]-2-methyl-5-nitro-1H-imidazole (TNZ-monoclinic) Geometric parameters (Å, º)

**Table:**

S1—O4	1.4385 (11)	C4—H4A	0.9600
S1—O3	1.4418 (11)	C4—H4B	0.9600
S1—C7	1.7711 (15)	C4—H4C	0.9600
S1—C6	1.7746 (12)	C5—C6	1.5210 (17)
O1—N3	1.2273 (18)	C5—H5A	0.9700
O2—N3	1.2288 (19)	C5—H5B	0.9700
N1—C3	1.3330 (18)	C6—H6A	0.9700
N1—C1	1.348 (2)	C6—H6B	0.9700
N2—C3	1.3566 (17)	C7—C8	1.516 (2)
N2—C2	1.3813 (18)	C7—H7A	0.9700
N2—C5	1.4678 (16)	C7—H7B	0.9700
N3—C2	1.4086 (18)	C8—H8A	0.9600
C1—C2	1.366 (2)	C8—H8B	0.9600
C1—H1	0.9300	C8—H8C	0.9600
C3—C4	1.478 (2)		
			
O4—S1—O3	118.05 (7)	H4A—C4—H4C	109.5
O4—S1—C7	108.85 (7)	H4B—C4—H4C	109.5
O3—S1—C7	107.79 (7)	N2—C5—C6	109.96 (10)
O4—S1—C6	107.24 (6)	N2—C5—H5A	109.7
O3—S1—C6	107.76 (6)	C6—C5—H5A	109.7
C7—S1—C6	106.62 (7)	N2—C5—H5B	109.7
C3—N1—C1	105.83 (12)	C6—C5—H5B	109.7
C3—N2—C2	105.16 (11)	H5A—C5—H5B	108.2
C3—N2—C5	126.02 (11)	C5—C6—S1	113.13 (9)
C2—N2—C5	128.69 (11)	C5—C6—H6A	109.0
O1—N3—O2	123.21 (14)	S1—C6—H6A	109.0
O1—N3—C2	116.94 (14)	C5—C6—H6B	109.0
O2—N3—C2	119.84 (13)	S1—C6—H6B	109.0
N1—C1—C2	109.79 (13)	H6A—C6—H6B	107.8
N1—C1—H1	125.1	C8—C7—S1	114.16 (11)
C2—C1—H1	125.1	C8—C7—H7A	108.7
C1—C2—N2	107.15 (12)	S1—C7—H7A	108.7
C1—C2—N3	127.33 (14)	C8—C7—H7B	108.7
N2—C2—N3	125.51 (13)	S1—C7—H7B	108.7
N1—C3—N2	112.06 (12)	H7A—C7—H7B	107.6
N1—C3—C4	124.03 (12)	C7—C8—H8A	109.5
N2—C3—C4	123.90 (12)	C7—C8—H8B	109.5
C3—C4—H4A	109.5	H8A—C8—H8B	109.5
C3—C4—H4B	109.5	C7—C8—H8C	109.5
H4A—C4—H4B	109.5	H8A—C8—H8C	109.5
C3—C4—H4C	109.5	H8B—C8—H8C	109.5
			
C3—N1—C1—C2	−0.03 (17)	C2—N2—C3—N1	−1.16 (15)
N1—C1—C2—N2	−0.68 (17)	C5—N2—C3—N1	−177.30 (11)
N1—C1—C2—N3	−179.68 (13)	C2—N2—C3—C4	177.50 (13)
C3—N2—C2—C1	1.08 (15)	C5—N2—C3—C4	1.4 (2)
C5—N2—C2—C1	177.08 (12)	C3—N2—C5—C6	97.29 (15)
C3—N2—C2—N3	−179.89 (13)	C2—N2—C5—C6	−77.93 (16)
C5—N2—C2—N3	−3.9 (2)	N2—C5—C6—S1	170.06 (8)
O1—N3—C2—C1	−1.8 (2)	O4—S1—C6—C5	166.17 (10)
O2—N3—C2—C1	177.36 (16)	O3—S1—C6—C5	38.12 (12)
O1—N3—C2—N2	179.39 (14)	C7—S1—C6—C5	−77.36 (11)
O2—N3—C2—N2	−1.5 (2)	O4—S1—C7—C8	49.01 (14)
C1—N1—C3—N2	0.76 (16)	O3—S1—C7—C8	178.16 (12)
C1—N1—C3—C4	−177.90 (14)	C6—S1—C7—C8	−66.38 (13)

### 1-[2-(Ethanesulfonyl)ethyl]-2-methyl-5-nitro-1H-imidazole (TNZ-monoclinic) Hydrogen-bond geometry (Å, º)

**Table:**

*D*—H···*A*	*D*—H	H···*A*	*D*···*A*	*D*—H···*A*
C1—H1···O1^i^	0.93	2.45	3.374 (2)	171
C6—H6*A*···O3^ii^	0.97	2.53	3.3777 (17)	146
C6—H6*B*···O3^iii^	0.97	2.53	3.2942 (17)	136

Symmetry codes: (i) −*x*+1, −*y*+2, −*z*+1; (ii) *x*, *y*+1, *z*; (iii) −*x*, −*y*, −*z*+1.

### 1-[2-(Ethanesulfonyl)ethyl]-2-methyl-5-nitro-1H-imidazole (TNZ-triclinic) Crystal data

**Table:**

C_8_H_13_N_3_O_4_S	*Z* = 4
*M**_r_* = 247.27	*F*(000) = 520
Triclinic, *P*1	*D*_x_ = 1.456 Mg m^−^^3^
*a* = 5.7208 (2) Å	Cu *K*α radiation, λ = 1.54184 Å
*b* = 13.2759 (5) Å	Cell parameters from 6574 reflections
*c* = 15.5932 (5) Å	θ = 2.9–76.0°
α = 77.101 (3)°	µ = 2.64 mm^−^^1^
β = 85.407 (2)°	*T* = 294 K
γ = 77.923 (3)°	Needle, colourless
*V* = 1128.16 (7) Å^3^	0.23 × 0.04 × 0.02 mm

### 1-[2-(Ethanesulfonyl)ethyl]-2-methyl-5-nitro-1H-imidazole (TNZ-triclinic) Data collection

**Table:**

Rigaku XtaLAB Synergy, Dualflex, HyPix diffractometer	4260 independent reflections
Radiation source: micro-focus sealed X-ray tube, PhotonJet (Cu) X-ray Source	3758 reflections with *I* > 2σ(*I*)
Mirror monochromator	*R*_int_ = 0.032
Detector resolution: 10.0000 pixels mm^-1^	θ_max_ = 72.1°, θ_min_ = 2.9°
ω scans	*h* = −5→6
Absorption correction: gaussian (CrysAlisPro 1.171.42.88a; Rigaku OD, 2023)	*k* = −16→16
*T*_min_ = 0.496, *T*_max_ = 1.000	*l* = −19→19
11280 measured reflections	

### 1-[2-(Ethanesulfonyl)ethyl]-2-methyl-5-nitro-1H-imidazole (TNZ-triclinic) Refinement

**Table:**

Refinement on *F*^2^	Primary atom site location: structure-invariant direct methods
Least-squares matrix: full	Hydrogen site location: inferred from neighbouring sites
*R*[*F*^2^ > 2σ(*F*^2^)] = 0.039	H-atom parameters constrained
*wR*(*F*^2^) = 0.103	*w* = 1/[σ^2^(*F*_o_^2^) + (0.0533*P*)^2^ + 0.3451*P*] where *P* = (*F*_o_^2^ + 2*F*_c_^2^)/3
*S* = 1.03	(Δ/σ)_max_ < 0.001
4260 reflections	Δρ_max_ = 0.20 e Å^−^^3^
293 parameters	Δρ_min_ = −0.34 e Å^−^^3^
0 restraints	

### 1-[2-(Ethanesulfonyl)ethyl]-2-methyl-5-nitro-1H-imidazole (TNZ-triclinic) Special details

**Table:**

Geometry. All esds (except the esd in the dihedral angle between two l.s. planes) are estimated using the full covariance matrix. The cell esds are taken into account individually in the estimation of esds in distances, angles and torsion angles; correlations between esds in cell parameters are only used when they are defined by crystal symmetry. An approximate (isotropic) treatment of cell esds is used for estimating esds involving l.s. planes.

### 1-[2-(Ethanesulfonyl)ethyl]-2-methyl-5-nitro-1H-imidazole (TNZ-triclinic) Fractional atomic coordinates and isotropic or equivalent isotropic displacement parameters (Å^2^)

**Table:**

	*x*	*y*	*z*	*U*_iso_*/*U*_eq_	
S1	0.08813 (8)	0.71568 (4)	0.16126 (3)	0.03887 (13)	
O1	0.6015 (3)	0.35548 (15)	0.08904 (12)	0.0659 (5)	
O2	0.2507 (3)	0.43620 (14)	0.04360 (11)	0.0615 (4)	
O3	0.3279 (3)	0.66741 (13)	0.18638 (12)	0.0643 (5)	
O4	−0.0825 (3)	0.73682 (14)	0.23036 (11)	0.0658 (5)	
N1	0.3266 (3)	0.39747 (15)	0.33339 (12)	0.0525 (4)	
N2	0.1110 (3)	0.46958 (12)	0.21403 (10)	0.0372 (3)	
N3	0.3968 (3)	0.40279 (13)	0.10195 (12)	0.0446 (4)	
C1	0.4581 (4)	0.37273 (17)	0.26206 (15)	0.0480 (5)	
H1	0.612177	0.332307	0.263463	0.058*	
C2	0.3309 (3)	0.41597 (14)	0.18791 (13)	0.0390 (4)	
C3	0.1200 (4)	0.45513 (16)	0.30271 (14)	0.0445 (4)	
C4	−0.0803 (5)	0.4971 (2)	0.35979 (17)	0.0616 (6)	
H4A	−0.035545	0.475548	0.420169	0.092*	
H4B	−0.219289	0.470259	0.352702	0.092*	
H4C	−0.115617	0.572689	0.343391	0.092*	
C5	−0.0842 (3)	0.53641 (15)	0.15968 (14)	0.0413 (4)	
H5A	−0.219792	0.556701	0.197978	0.050*	
H5B	−0.133638	0.495553	0.122507	0.050*	
C6	−0.0180 (4)	0.63580 (15)	0.10156 (13)	0.0421 (4)	
H6A	−0.157448	0.677088	0.069842	0.050*	
H6B	0.104571	0.615625	0.058473	0.050*	
C7	0.0924 (4)	0.83392 (17)	0.08253 (15)	0.0518 (5)	
H7A	0.179742	0.817786	0.029758	0.062*	
H7B	−0.070161	0.868092	0.066767	0.062*	
C8	0.2082 (5)	0.90791 (19)	0.11768 (17)	0.0636 (6)	
H8A	0.126938	0.921067	0.171570	0.095*	
H8B	0.198259	0.973191	0.075238	0.095*	
H8C	0.373046	0.876575	0.128607	0.095*	
S2	0.37980 (7)	−0.12502 (4)	0.38486 (3)	0.03747 (13)	
O5	0.3989 (3)	0.15492 (15)	0.04148 (11)	0.0672 (5)	
O6	0.6738 (3)	0.07162 (15)	0.13344 (11)	0.0662 (5)	
O7	0.1508 (2)	−0.08429 (13)	0.34643 (11)	0.0555 (4)	
O8	0.3992 (3)	−0.11542 (14)	0.47345 (10)	0.0598 (4)	
N4	−0.0068 (3)	0.21724 (14)	0.25810 (13)	0.0500 (4)	
N5	0.3589 (2)	0.11938 (12)	0.27507 (10)	0.0359 (3)	
N6	0.4724 (3)	0.12279 (14)	0.11701 (11)	0.0463 (4)	
C11	0.0886 (4)	0.20747 (17)	0.17801 (15)	0.0479 (5)	
H11	0.012668	0.237178	0.125129	0.057*	
C12	0.3125 (3)	0.14772 (15)	0.18598 (12)	0.0384 (4)	
C13	0.1580 (3)	0.16381 (15)	0.31583 (14)	0.0419 (4)	
C14	0.1216 (4)	0.1555 (2)	0.41149 (15)	0.0580 (6)	
H14A	−0.041359	0.185720	0.424524	0.087*	
H14B	0.226514	0.192617	0.431389	0.087*	
H14C	0.156027	0.082606	0.440929	0.087*	
C15	0.5715 (3)	0.05185 (16)	0.31960 (13)	0.0408 (4)	
H15A	0.562506	0.058683	0.380487	0.049*	
H15B	0.712940	0.076557	0.291887	0.049*	
C16	0.5981 (3)	−0.06392 (16)	0.31728 (14)	0.0409 (4)	
H16A	0.756089	−0.101049	0.336541	0.049*	
H16B	0.585441	−0.070028	0.257059	0.049*	
C17	0.4715 (4)	−0.25926 (17)	0.37811 (16)	0.0545 (5)	
H17A	0.467919	−0.265187	0.317362	0.065*	
H17B	0.634963	−0.284489	0.396862	0.065*	
C18	0.3141 (6)	−0.3267 (2)	0.4341 (2)	0.0834 (9)	
H18A	0.327090	−0.325432	0.494834	0.125*	
H18B	0.362940	−0.397781	0.426043	0.125*	
H18C	0.151149	−0.300131	0.417356	0.125*	

### 1-[2-(Ethanesulfonyl)ethyl]-2-methyl-5-nitro-1H-imidazole (TNZ-triclinic) Atomic displacement parameters (Å^2^)

**Table:**

	*U*^11^	*U*^22^	*U*^33^	*U*^12^	*U*^13^	*U*^23^
S1	0.0419 (3)	0.0380 (2)	0.0388 (2)	−0.01155 (19)	−0.00137 (18)	−0.00918 (18)
O1	0.0476 (9)	0.0757 (11)	0.0699 (11)	0.0028 (8)	0.0091 (8)	−0.0241 (9)
O2	0.0660 (10)	0.0653 (10)	0.0539 (9)	0.0017 (8)	−0.0151 (8)	−0.0235 (8)
O3	0.0525 (9)	0.0580 (10)	0.0845 (12)	−0.0083 (7)	−0.0290 (8)	−0.0122 (8)
O4	0.0839 (12)	0.0631 (10)	0.0604 (10)	−0.0320 (9)	0.0282 (8)	−0.0282 (8)
N1	0.0580 (11)	0.0486 (10)	0.0486 (10)	−0.0066 (8)	−0.0104 (8)	−0.0063 (8)
N2	0.0351 (8)	0.0330 (8)	0.0444 (9)	−0.0081 (6)	−0.0017 (6)	−0.0083 (6)
N3	0.0442 (9)	0.0394 (9)	0.0519 (10)	−0.0074 (7)	−0.0008 (7)	−0.0142 (7)
C1	0.0435 (11)	0.0446 (11)	0.0550 (12)	−0.0042 (9)	−0.0088 (9)	−0.0100 (9)
C2	0.0361 (9)	0.0351 (9)	0.0473 (10)	−0.0080 (7)	−0.0014 (8)	−0.0106 (8)
C3	0.0510 (11)	0.0369 (10)	0.0464 (11)	−0.0127 (8)	0.0007 (9)	−0.0074 (8)
C4	0.0674 (15)	0.0615 (14)	0.0545 (13)	−0.0129 (12)	0.0131 (11)	−0.0146 (11)
C5	0.0318 (9)	0.0400 (10)	0.0542 (11)	−0.0068 (7)	−0.0064 (8)	−0.0126 (9)
C6	0.0425 (10)	0.0398 (10)	0.0448 (10)	−0.0049 (8)	−0.0096 (8)	−0.0109 (8)
C7	0.0632 (13)	0.0452 (11)	0.0484 (12)	−0.0201 (10)	−0.0047 (10)	−0.0031 (9)
C8	0.0848 (17)	0.0508 (13)	0.0629 (15)	−0.0309 (12)	0.0024 (12)	−0.0132 (11)
S2	0.0268 (2)	0.0443 (3)	0.0422 (3)	−0.00891 (17)	0.00138 (17)	−0.01027 (19)
O5	0.0749 (11)	0.0832 (12)	0.0417 (9)	−0.0178 (9)	−0.0001 (8)	−0.0083 (8)
O6	0.0485 (9)	0.0793 (12)	0.0586 (10)	0.0029 (8)	0.0110 (7)	−0.0081 (8)
O7	0.0262 (7)	0.0632 (10)	0.0777 (11)	−0.0106 (6)	−0.0057 (6)	−0.0129 (8)
O8	0.0719 (10)	0.0717 (10)	0.0418 (8)	−0.0265 (8)	0.0025 (7)	−0.0150 (7)
N4	0.0349 (8)	0.0463 (10)	0.0644 (11)	−0.0033 (7)	0.0005 (8)	−0.0080 (8)
N5	0.0291 (7)	0.0379 (8)	0.0409 (8)	−0.0077 (6)	0.0009 (6)	−0.0085 (6)
N6	0.0471 (10)	0.0475 (10)	0.0447 (9)	−0.0150 (8)	0.0048 (7)	−0.0076 (7)
C11	0.0393 (10)	0.0488 (12)	0.0524 (12)	−0.0083 (9)	−0.0065 (9)	−0.0026 (9)
C12	0.0347 (9)	0.0397 (10)	0.0413 (10)	−0.0114 (7)	0.0013 (7)	−0.0070 (8)
C13	0.0348 (9)	0.0392 (10)	0.0519 (11)	−0.0090 (8)	0.0045 (8)	−0.0105 (8)
C14	0.0567 (13)	0.0627 (14)	0.0538 (13)	−0.0071 (11)	0.0126 (10)	−0.0197 (11)
C15	0.0273 (8)	0.0488 (11)	0.0475 (11)	−0.0113 (8)	−0.0046 (7)	−0.0082 (8)
C16	0.0224 (8)	0.0465 (11)	0.0500 (11)	−0.0050 (7)	0.0027 (7)	−0.0057 (8)
C17	0.0534 (12)	0.0468 (12)	0.0616 (14)	−0.0098 (10)	0.0108 (10)	−0.0124 (10)
C18	0.098 (2)	0.0563 (15)	0.098 (2)	−0.0314 (15)	0.0356 (17)	−0.0194 (15)

### 1-[2-(Ethanesulfonyl)ethyl]-2-methyl-5-nitro-1H-imidazole (TNZ-triclinic) Geometric parameters (Å, º)

**Table:**

S1—O4	1.4298 (17)	S2—O8	1.4304 (16)
S1—O3	1.4322 (16)	S2—O7	1.4324 (14)
S1—C7	1.767 (2)	S2—C17	1.774 (2)
S1—C6	1.7777 (19)	S2—C16	1.7777 (18)
O1—N3	1.232 (2)	O5—N6	1.235 (2)
O2—N3	1.229 (2)	O6—N6	1.224 (2)
N1—C3	1.327 (3)	N4—C13	1.332 (3)
N1—C1	1.356 (3)	N4—C11	1.345 (3)
N2—C3	1.357 (3)	N5—C13	1.356 (2)
N2—C2	1.388 (2)	N5—C12	1.388 (2)
N2—C5	1.468 (2)	N5—C15	1.472 (2)
N3—C2	1.402 (3)	N6—C12	1.406 (3)
C1—C2	1.364 (3)	C11—C12	1.357 (3)
C1—H1	0.9300	C11—H11	0.9300
C3—C4	1.486 (3)	C13—C14	1.472 (3)
C4—H4A	0.9600	C14—H14A	0.9600
C4—H4B	0.9600	C14—H14B	0.9600
C4—H4C	0.9600	C14—H14C	0.9600
C5—C6	1.525 (3)	C15—C16	1.521 (3)
C5—H5A	0.9700	C15—H15A	0.9700
C5—H5B	0.9700	C15—H15B	0.9700
C6—H6A	0.9700	C16—H16A	0.9700
C6—H6B	0.9700	C16—H16B	0.9700
C7—C8	1.508 (3)	C17—C18	1.496 (3)
C7—H7A	0.9700	C17—H17A	0.9700
C7—H7B	0.9700	C17—H17B	0.9700
C8—H8A	0.9600	C18—H18A	0.9600
C8—H8B	0.9600	C18—H18B	0.9600
C8—H8C	0.9600	C18—H18C	0.9600
			
O4—S1—O3	116.91 (12)	O8—S2—O7	116.91 (10)
O4—S1—C7	109.09 (11)	O8—S2—C17	109.27 (11)
O3—S1—C7	109.09 (11)	O7—S2—C17	109.04 (11)
O4—S1—C6	108.83 (10)	O8—S2—C16	108.63 (10)
O3—S1—C6	108.82 (10)	O7—S2—C16	109.06 (9)
C7—S1—C6	103.23 (10)	C17—S2—C16	103.02 (10)
C3—N1—C1	105.66 (17)	C13—N4—C11	106.46 (17)
C3—N2—C2	104.93 (16)	C13—N5—C12	105.21 (15)
C3—N2—C5	125.70 (16)	C13—N5—C15	125.44 (16)
C2—N2—C5	129.15 (16)	C12—N5—C15	129.29 (15)
O2—N3—O1	122.79 (19)	O6—N6—O5	123.20 (19)
O2—N3—C2	119.86 (17)	O6—N6—C12	119.88 (17)
O1—N3—C2	117.33 (17)	O5—N6—C12	116.93 (18)
N1—C1—C2	109.84 (18)	N4—C11—C12	109.74 (18)
N1—C1—H1	125.1	N4—C11—H11	125.1
C2—C1—H1	125.1	C12—C11—H11	125.1
C1—C2—N2	107.05 (18)	C11—C12—N5	107.15 (17)
C1—C2—N3	127.48 (18)	C11—C12—N6	126.68 (18)
N2—C2—N3	125.28 (17)	N5—C12—N6	126.12 (16)
N1—C3—N2	112.52 (18)	N4—C13—N5	111.44 (18)
N1—C3—C4	123.4 (2)	N4—C13—C14	123.19 (18)
N2—C3—C4	124.04 (19)	N5—C13—C14	125.37 (18)
C3—C4—H4A	109.5	C13—C14—H14A	109.5
C3—C4—H4B	109.5	C13—C14—H14B	109.5
H4A—C4—H4B	109.5	H14A—C14—H14B	109.5
C3—C4—H4C	109.5	C13—C14—H14C	109.5
H4A—C4—H4C	109.5	H14A—C14—H14C	109.5
H4B—C4—H4C	109.5	H14B—C14—H14C	109.5
N2—C5—C6	113.80 (14)	N5—C15—C16	113.50 (14)
N2—C5—H5A	108.8	N5—C15—H15A	108.9
C6—C5—H5A	108.8	C16—C15—H15A	108.9
N2—C5—H5B	108.8	N5—C15—H15B	108.9
C6—C5—H5B	108.8	C16—C15—H15B	108.9
H5A—C5—H5B	107.7	H15A—C15—H15B	107.7
C5—C6—S1	113.50 (14)	C15—C16—S2	112.98 (13)
C5—C6—H6A	108.9	C15—C16—H16A	109.0
S1—C6—H6A	108.9	S2—C16—H16A	109.0
C5—C6—H6B	108.9	C15—C16—H16B	109.0
S1—C6—H6B	108.9	S2—C16—H16B	109.0
H6A—C6—H6B	107.7	H16A—C16—H16B	107.8
C8—C7—S1	111.26 (16)	C18—C17—S2	111.82 (18)
C8—C7—H7A	109.4	C18—C17—H17A	109.3
S1—C7—H7A	109.4	S2—C17—H17A	109.3
C8—C7—H7B	109.4	C18—C17—H17B	109.3
S1—C7—H7B	109.4	S2—C17—H17B	109.3
H7A—C7—H7B	108.0	H17A—C17—H17B	107.9
C7—C8—H8A	109.5	C17—C18—H18A	109.5
C7—C8—H8B	109.5	C17—C18—H18B	109.5
H8A—C8—H8B	109.5	H18A—C18—H18B	109.5
C7—C8—H8C	109.5	C17—C18—H18C	109.5
H8A—C8—H8C	109.5	H18A—C18—H18C	109.5
H8B—C8—H8C	109.5	H18B—C18—H18C	109.5
			
C3—N1—C1—C2	0.2 (2)	C13—N4—C11—C12	0.2 (2)
N1—C1—C2—N2	−0.2 (2)	N4—C11—C12—N5	−0.3 (2)
N1—C1—C2—N3	−175.24 (18)	N4—C11—C12—N6	−177.69 (18)
C3—N2—C2—C1	0.0 (2)	C13—N5—C12—C11	0.3 (2)
C5—N2—C2—C1	174.68 (17)	C15—N5—C12—C11	177.49 (17)
C3—N2—C2—N3	175.23 (17)	C13—N5—C12—N6	177.69 (17)
C5—N2—C2—N3	−10.1 (3)	C15—N5—C12—N6	−5.1 (3)
O2—N3—C2—C1	170.95 (19)	O6—N6—C12—C11	176.8 (2)
O1—N3—C2—C1	−7.7 (3)	O5—N6—C12—C11	−3.1 (3)
O2—N3—C2—N2	−3.3 (3)	O6—N6—C12—N5	−0.2 (3)
O1—N3—C2—N2	178.06 (18)	O5—N6—C12—N5	179.98 (18)
C1—N1—C3—N2	−0.2 (2)	C11—N4—C13—N5	0.0 (2)
C1—N1—C3—C4	178.6 (2)	C11—N4—C13—C14	180.0 (2)
C2—N2—C3—N1	0.2 (2)	C12—N5—C13—N4	−0.2 (2)
C5—N2—C3—N1	−174.77 (16)	C15—N5—C13—N4	−177.54 (16)
C2—N2—C3—C4	−178.68 (19)	C12—N5—C13—C14	179.9 (2)
C5—N2—C3—C4	6.4 (3)	C15—N5—C13—C14	2.5 (3)
C3—N2—C5—C6	109.6 (2)	C13—N5—C15—C16	107.0 (2)
C2—N2—C5—C6	−64.0 (2)	C12—N5—C15—C16	−69.7 (2)
N2—C5—C6—S1	−56.1 (2)	N5—C15—C16—S2	−70.36 (19)
O4—S1—C6—C5	−52.97 (17)	O8—S2—C16—C15	−58.53 (16)
O3—S1—C6—C5	75.45 (17)	O7—S2—C16—C15	69.93 (16)
C7—S1—C6—C5	−168.77 (15)	C17—S2—C16—C15	−174.34 (14)
O4—S1—C7—C8	71.6 (2)	O8—S2—C17—C18	62.5 (2)
O3—S1—C7—C8	−57.2 (2)	O7—S2—C17—C18	−66.4 (2)
C6—S1—C7—C8	−172.76 (18)	C16—S2—C17—C18	177.8 (2)

### 1-[2-(Ethanesulfonyl)ethyl]-2-methyl-5-nitro-1H-imidazole (TNZ-triclinic) Hydrogen-bond geometry (Å, º)

**Table:**

*D*—H···*A*	*D*—H	H···*A*	*D*···*A*	*D*—H···*A*
C1—H1···N4^i^	0.93	2.39	3.319 (3)	174
C4—H4*C*···O4	0.96	2.51	3.365 (3)	148
C6—H6*B*···O2	0.97	2.41	3.057 (3)	124
C16—H16*A*···O7^i^	0.97	2.34	3.177 (2)	145

Symmetry code: (i) *x*+1, *y*, *z*.

### 1-[2-(Ethanesulfonyl)ethyl]-2-methyl-5-nitro-1H-imidazole hemihydrate (TNZ-hemihydrate) Crystal data

**Table:**

C_8_H_13_N_3_O_4_S·0.5H_2_O	*Z* = 4
*M**_r_* = 256.28	*F*(000) = 540
Triclinic, *P*1	*D*_x_ = 1.464 Mg m^−^^3^
*a* = 5.7223 (1) Å	Cu *K*α radiation, λ = 1.54184 Å
*b* = 13.1854 (2) Å	Cell parameters from 6288 reflections
*c* = 16.2439 (4) Å	θ = 2.8–76.8°
α = 102.658 (2)°	µ = 2.61 mm^−^^1^
β = 92.226 (2)°	*T* = 294 K
γ = 102.484 (2)°	Plate, colourless
*V* = 1162.76 (4) Å^3^	0.22 × 0.06 × 0.03 mm

### 1-[2-(Ethanesulfonyl)ethyl]-2-methyl-5-nitro-1H-imidazole hemihydrate (TNZ-hemihydrate) Data collection

**Table:**

Rigaku XtaLAB Synergy, Dualflex, HyPix diffractometer	4366 independent reflections
Radiation source: micro-focus sealed X-ray tube, PhotonJet (Cu) X-ray Source	3775 reflections with *I* > 2σ(*I*)
Mirror monochromator	*R*_int_ = 0.029
Detector resolution: 10.0000 pixels mm^-1^	θ_max_ = 72.1°, θ_min_ = 2.8°
ω scans	*h* = −6→6
Absorption correction: gaussian (CrysAlisPro 1.171.44.109a; Rigaku OD, 2025)	*k* = −15→16
*T*_min_ = 0.372, *T*_max_ = 1.000	*l* = −19→19
11604 measured reflections	

### 1-[2-(Ethanesulfonyl)ethyl]-2-methyl-5-nitro-1H-imidazole hemihydrate (TNZ-hemihydrate) Refinement

**Table:**

Refinement on *F*^2^	Primary atom site location: structure-invariant direct methods
Least-squares matrix: full	Hydrogen site location: mixed
*R*[*F*^2^ > 2σ(*F*^2^)] = 0.037	H-atom parameters constrained
*wR*(*F*^2^) = 0.101	*w* = 1/[σ^2^(*F*_o_^2^) + (0.0477*P*)^2^ + 0.3462*P*] where *P* = (*F*_o_^2^ + 2*F*_c_^2^)/3
*S* = 1.06	(Δ/σ)_max_ < 0.001
4366 reflections	Δρ_max_ = 0.22 e Å^−^^3^
311 parameters	Δρ_min_ = −0.34 e Å^−^^3^
0 restraints	

### 1-[2-(Ethanesulfonyl)ethyl]-2-methyl-5-nitro-1H-imidazole hemihydrate (TNZ-hemihydrate) Special details

**Table:**

Geometry. All esds (except the esd in the dihedral angle between two l.s. planes) are estimated using the full covariance matrix. The cell esds are taken into account individually in the estimation of esds in distances, angles and torsion angles; correlations between esds in cell parameters are only used when they are defined by crystal symmetry. An approximate (isotropic) treatment of cell esds is used for estimating esds involving l.s. planes.

### 1-[2-(Ethanesulfonyl)ethyl]-2-methyl-5-nitro-1H-imidazole hemihydrate (TNZ-hemihydrate) Fractional atomic coordinates and isotropic or equivalent isotropic displacement parameters (Å^2^)

**Table:**

	*x*	*y*	*z*	*U*_iso_*/*U*_eq_	Occ. (<1)
S1	0.06312 (8)	0.19701 (3)	0.34587 (3)	0.03670 (13)	
O1	0.5927 (3)	0.59801 (13)	0.41560 (10)	0.0598 (4)	
O2	0.2463 (3)	0.54181 (12)	0.45934 (10)	0.0567 (4)	
O3	0.2978 (3)	0.23351 (12)	0.32065 (11)	0.0600 (4)	
O4	−0.1210 (3)	0.13782 (12)	0.28019 (10)	0.0610 (4)	
N1	0.2776 (3)	0.42349 (13)	0.18121 (11)	0.0445 (4)	
N2	0.0788 (2)	0.41595 (11)	0.29565 (9)	0.0322 (3)	
N3	0.3841 (3)	0.54354 (12)	0.40339 (11)	0.0406 (4)	
C1	0.4220 (3)	0.48625 (15)	0.24956 (13)	0.0414 (4)	
H1	0.577359	0.525338	0.248263	0.050*	
C2	0.3041 (3)	0.48326 (13)	0.32058 (12)	0.0345 (4)	
C3	0.0728 (3)	0.38224 (14)	0.21032 (12)	0.0385 (4)	
C4	−0.1373 (4)	0.31084 (18)	0.15518 (15)	0.0536 (5)	
H4A	−0.097973	0.297138	0.097469	0.080*	
H4B	−0.270779	0.344428	0.159738	0.080*	
H4C	−0.179255	0.244658	0.172555	0.080*	
C5	−0.1095 (3)	0.37753 (14)	0.34807 (13)	0.0374 (4)	
H5A	−0.251378	0.336346	0.311261	0.045*	
H5B	−0.152533	0.438612	0.384004	0.045*	
C6	−0.0342 (3)	0.30867 (14)	0.40362 (12)	0.0389 (4)	
H6A	−0.169055	0.283435	0.434042	0.047*	
H6B	0.094910	0.352426	0.445184	0.047*	
C7	0.0842 (4)	0.12037 (17)	0.42135 (14)	0.0505 (5)	
H7A	0.180273	0.165394	0.471992	0.061*	
H7B	−0.075006	0.092879	0.436917	0.061*	
C8	0.1977 (5)	0.02831 (19)	0.38607 (17)	0.0630 (6)	
H8A	0.105881	−0.014768	0.334811	0.094*	
H8B	0.199980	−0.014396	0.426835	0.094*	
H8C	0.359157	0.055656	0.374176	0.094*	
S2	0.32313 (8)	0.91695 (4)	0.12417 (3)	0.03969 (14)	
O5	0.3930 (3)	0.82214 (15)	0.45473 (11)	0.0687 (5)	
O6	0.6570 (3)	0.85724 (15)	0.36754 (13)	0.0730 (5)	
O7	0.1009 (2)	0.89156 (12)	0.16127 (11)	0.0557 (4)	
O8	0.3327 (3)	0.86236 (13)	0.03818 (11)	0.0689 (5)	
N4	−0.0398 (3)	0.63948 (14)	0.24541 (13)	0.0508 (4)	
N5	0.3248 (3)	0.73073 (12)	0.22998 (11)	0.0392 (4)	
N6	0.4578 (3)	0.81414 (14)	0.38273 (13)	0.0502 (4)	
C11	0.0651 (4)	0.69415 (17)	0.32269 (15)	0.0481 (5)	
H11	−0.004702	0.693030	0.373339	0.058*	
C12	0.2881 (3)	0.75113 (15)	0.31547 (13)	0.0406 (4)	
C13	0.1176 (3)	0.66266 (15)	0.19065 (14)	0.0433 (4)	
C14	0.0703 (5)	0.6189 (2)	0.09837 (16)	0.0618 (6)	
H14A	−0.079136	0.566290	0.086391	0.093*	
H14B	0.198078	0.586382	0.077814	0.093*	
H14C	0.061139	0.675508	0.070828	0.093*	
C15	0.5312 (3)	0.77637 (17)	0.18787 (15)	0.0475 (5)	
H15A	0.516369	0.735990	0.129565	0.057*	
H15B	0.677263	0.768569	0.215659	0.057*	
C16	0.5539 (3)	0.89331 (16)	0.18868 (15)	0.0451 (5)	
H16A	0.708104	0.920972	0.169478	0.054*	
H16B	0.551612	0.932516	0.246506	0.054*	
C17	0.4085 (4)	1.05667 (17)	0.13349 (15)	0.0501 (5)	
H17A	0.436096	1.092856	0.193017	0.060*	
H17B	0.558170	1.073550	0.107963	0.060*	
C18	0.2209 (5)	1.0974 (2)	0.09156 (19)	0.0675 (7)	
H18A	0.197008	1.063625	0.032202	0.101*	
H18B	0.273240	1.173282	0.098854	0.101*	
H18C	0.072540	1.081162	0.116810	0.101*	
O1WA	0.4788 (10)	0.4248 (4)	0.0197 (3)	0.0949 (15)	0.5
H1WA	0.519771	0.492071	0.024906	0.142*	0.5
H1WB	0.391343	0.417101	0.059977	0.142*	0.5
O1WB	0.4306 (11)	0.3534 (4)	0.0155 (3)	0.0893 (15)	0.5
H1WC	0.416298	0.380212	0.067167	0.134*	0.5
H1WD	0.486427	0.298840	0.016674	0.134*	0.5

### 1-[2-(Ethanesulfonyl)ethyl]-2-methyl-5-nitro-1H-imidazole hemihydrate (TNZ-hemihydrate) Atomic displacement parameters (Å^2^)

**Table:**

	*U*^11^	*U*^22^	*U*^33^	*U*^12^	*U*^13^	*U*^23^
S1	0.0392 (2)	0.0357 (2)	0.0367 (2)	0.01152 (18)	0.00552 (18)	0.00834 (18)
O1	0.0452 (8)	0.0623 (10)	0.0577 (10)	−0.0061 (7)	−0.0044 (7)	0.0039 (8)
O2	0.0656 (10)	0.0526 (9)	0.0419 (8)	0.0033 (7)	0.0177 (7)	−0.0022 (7)
O3	0.0532 (9)	0.0593 (9)	0.0772 (11)	0.0204 (7)	0.0325 (8)	0.0246 (8)
O4	0.0734 (11)	0.0485 (8)	0.0548 (9)	0.0189 (8)	−0.0203 (8)	−0.0014 (7)
N1	0.0508 (9)	0.0448 (9)	0.0395 (9)	0.0113 (7)	0.0115 (8)	0.0114 (7)
N2	0.0322 (7)	0.0300 (7)	0.0351 (8)	0.0082 (6)	0.0058 (6)	0.0072 (6)
N3	0.0441 (9)	0.0341 (8)	0.0430 (9)	0.0095 (7)	0.0047 (7)	0.0073 (7)
C1	0.0376 (10)	0.0394 (10)	0.0473 (11)	0.0066 (8)	0.0095 (9)	0.0117 (9)
C2	0.0337 (9)	0.0310 (8)	0.0390 (10)	0.0082 (7)	0.0046 (7)	0.0076 (7)
C3	0.0443 (10)	0.0336 (9)	0.0394 (10)	0.0117 (8)	0.0034 (8)	0.0100 (8)
C4	0.0555 (13)	0.0510 (12)	0.0475 (12)	0.0057 (10)	−0.0068 (10)	0.0058 (10)
C5	0.0313 (9)	0.0357 (9)	0.0464 (11)	0.0091 (7)	0.0114 (8)	0.0093 (8)
C6	0.0418 (10)	0.0362 (9)	0.0398 (10)	0.0088 (8)	0.0138 (8)	0.0093 (8)
C7	0.0609 (13)	0.0509 (12)	0.0479 (12)	0.0220 (10)	0.0096 (10)	0.0187 (10)
C8	0.0820 (17)	0.0505 (13)	0.0653 (16)	0.0301 (12)	0.0048 (13)	0.0179 (12)
S2	0.0318 (2)	0.0425 (3)	0.0425 (3)	0.00384 (18)	0.00391 (18)	0.0095 (2)
O5	0.0777 (12)	0.0781 (12)	0.0484 (10)	0.0182 (9)	0.0013 (9)	0.0111 (9)
O6	0.0500 (10)	0.0825 (12)	0.0740 (12)	−0.0103 (8)	−0.0102 (8)	0.0200 (10)
O7	0.0271 (7)	0.0615 (9)	0.0823 (11)	0.0072 (6)	0.0107 (7)	0.0265 (8)
O8	0.0940 (13)	0.0596 (10)	0.0440 (9)	0.0077 (9)	0.0080 (9)	0.0026 (8)
N4	0.0366 (9)	0.0479 (10)	0.0684 (13)	0.0039 (7)	0.0043 (8)	0.0202 (9)
N5	0.0314 (8)	0.0354 (8)	0.0519 (10)	0.0088 (6)	0.0046 (7)	0.0110 (7)
N6	0.0462 (10)	0.0457 (9)	0.0601 (12)	0.0116 (8)	−0.0031 (9)	0.0160 (9)
C11	0.0417 (11)	0.0494 (11)	0.0570 (13)	0.0106 (9)	0.0097 (10)	0.0194 (10)
C12	0.0386 (10)	0.0367 (9)	0.0490 (11)	0.0124 (8)	0.0033 (8)	0.0119 (8)
C13	0.0364 (10)	0.0366 (10)	0.0570 (13)	0.0093 (8)	0.0004 (9)	0.0108 (9)
C14	0.0629 (14)	0.0535 (13)	0.0597 (15)	0.0039 (11)	−0.0033 (12)	0.0047 (11)
C15	0.0313 (10)	0.0517 (12)	0.0633 (14)	0.0137 (8)	0.0114 (9)	0.0163 (10)
C16	0.0265 (9)	0.0499 (11)	0.0591 (13)	0.0032 (8)	0.0056 (8)	0.0185 (10)
C17	0.0466 (11)	0.0441 (11)	0.0556 (13)	0.0024 (9)	0.0001 (10)	0.0121 (10)
C18	0.0635 (15)	0.0605 (14)	0.0835 (19)	0.0179 (12)	0.0003 (13)	0.0251 (14)
O1WA	0.097 (4)	0.125 (4)	0.072 (3)	0.039 (4)	0.032 (3)	0.025 (4)
O1WB	0.125 (4)	0.112 (4)	0.055 (2)	0.056 (4)	0.032 (3)	0.036 (3)

### 1-[2-(Ethanesulfonyl)ethyl]-2-methyl-5-nitro-1H-imidazole hemihydrate (TNZ-hemihydrate) Geometric parameters (Å, º)

**Table:**

S1—O4	1.4316 (16)	S2—C17	1.772 (2)
S1—O3	1.4328 (15)	S2—C16	1.776 (2)
S1—C7	1.767 (2)	O5—N6	1.230 (3)
S1—C6	1.7742 (18)	O6—N6	1.220 (2)
O1—N3	1.237 (2)	N4—C13	1.326 (3)
O2—N3	1.227 (2)	N4—C11	1.347 (3)
N1—C3	1.332 (2)	N5—C13	1.360 (2)
N1—C1	1.353 (3)	N5—C12	1.389 (3)
N2—C3	1.356 (2)	N5—C15	1.470 (2)
N2—C2	1.386 (2)	N6—C12	1.413 (3)
N2—C5	1.472 (2)	C11—C12	1.357 (3)
N3—C2	1.406 (2)	C11—H11	0.9300
C1—C2	1.363 (3)	C13—C14	1.475 (3)
C1—H1	0.9300	C14—H14A	0.9600
C3—C4	1.480 (3)	C14—H14B	0.9600
C4—H4A	0.9600	C14—H14C	0.9600
C4—H4B	0.9600	C15—C16	1.516 (3)
C4—H4C	0.9600	C15—H15A	0.9700
C5—C6	1.524 (3)	C15—H15B	0.9700
C5—H5A	0.9700	C16—H16A	0.9700
C5—H5B	0.9700	C16—H16B	0.9700
C6—H6A	0.9700	C17—C18	1.502 (3)
C6—H6B	0.9700	C17—H17A	0.9700
C7—C8	1.513 (3)	C17—H17B	0.9700
C7—H7A	0.9700	C18—H18A	0.9600
C7—H7B	0.9700	C18—H18B	0.9600
C8—H8A	0.9600	C18—H18C	0.9600
C8—H8B	0.9600	O1WA—H1WA	0.8507
C8—H8C	0.9600	O1WA—H1WB	0.8501
S2—O8	1.4334 (17)	O1WB—H1WC	0.8506
S2—O7	1.4341 (14)	O1WB—H1WD	0.8506
			
O4—S1—O3	117.14 (11)	O8—S2—C17	109.38 (11)
O4—S1—C7	109.10 (11)	O7—S2—C17	109.33 (10)
O3—S1—C7	109.08 (10)	O8—S2—C16	108.76 (11)
O4—S1—C6	108.40 (9)	O7—S2—C16	108.19 (9)
O3—S1—C6	108.77 (9)	C17—S2—C16	102.94 (10)
C7—S1—C6	103.48 (9)	C13—N4—C11	106.33 (17)
C3—N1—C1	106.27 (16)	C13—N5—C12	104.78 (16)
C3—N2—C2	105.13 (15)	C13—N5—C15	125.91 (18)
C3—N2—C5	125.28 (15)	C12—N5—C15	129.19 (17)
C2—N2—C5	129.30 (15)	O6—N6—O5	123.5 (2)
O2—N3—O1	123.05 (18)	O6—N6—C12	119.7 (2)
O2—N3—C2	119.69 (16)	O5—N6—C12	116.80 (18)
O1—N3—C2	117.24 (16)	N4—C11—C12	109.71 (19)
N1—C1—C2	109.36 (17)	N4—C11—H11	125.1
N1—C1—H1	125.3	C12—C11—H11	125.1
C2—C1—H1	125.3	C11—C12—N5	107.30 (18)
C1—C2—N2	107.41 (16)	C11—C12—N6	126.5 (2)
C1—C2—N3	127.15 (17)	N5—C12—N6	126.14 (17)
N2—C2—N3	125.23 (16)	N4—C13—N5	111.88 (19)
N1—C3—N2	111.83 (17)	N4—C13—C14	123.31 (19)
N1—C3—C4	123.55 (18)	N5—C13—C14	124.81 (19)
N2—C3—C4	124.60 (18)	C13—C14—H14A	109.5
C3—C4—H4A	109.5	C13—C14—H14B	109.5
C3—C4—H4B	109.5	H14A—C14—H14B	109.5
H4A—C4—H4B	109.5	C13—C14—H14C	109.5
C3—C4—H4C	109.5	H14A—C14—H14C	109.5
H4A—C4—H4C	109.5	H14B—C14—H14C	109.5
H4B—C4—H4C	109.5	N5—C15—C16	113.65 (15)
N2—C5—C6	113.47 (14)	N5—C15—H15A	108.8
N2—C5—H5A	108.9	C16—C15—H15A	108.8
C6—C5—H5A	108.9	N5—C15—H15B	108.8
N2—C5—H5B	108.9	C16—C15—H15B	108.8
C6—C5—H5B	108.9	H15A—C15—H15B	107.7
H5A—C5—H5B	107.7	C15—C16—S2	113.79 (15)
C5—C6—S1	113.48 (13)	C15—C16—H16A	108.8
C5—C6—H6A	108.9	S2—C16—H16A	108.8
S1—C6—H6A	108.9	C15—C16—H16B	108.8
C5—C6—H6B	108.9	S2—C16—H16B	108.8
S1—C6—H6B	108.9	H16A—C16—H16B	107.7
H6A—C6—H6B	107.7	C18—C17—S2	112.26 (16)
C8—C7—S1	110.75 (16)	C18—C17—H17A	109.2
C8—C7—H7A	109.5	S2—C17—H17A	109.2
S1—C7—H7A	109.5	C18—C17—H17B	109.2
C8—C7—H7B	109.5	S2—C17—H17B	109.2
S1—C7—H7B	109.5	H17A—C17—H17B	107.9
H7A—C7—H7B	108.1	C17—C18—H18A	109.5
C7—C8—H8A	109.5	C17—C18—H18B	109.5
C7—C8—H8B	109.5	H18A—C18—H18B	109.5
H8A—C8—H8B	109.5	C17—C18—H18C	109.5
C7—C8—H8C	109.5	H18A—C18—H18C	109.5
H8A—C8—H8C	109.5	H18B—C18—H18C	109.5
H8B—C8—H8C	109.5	H1WA—O1WA—H1WB	104.4
O8—S2—O7	117.27 (11)	H1WC—O1WB—H1WD	104.6
			
C3—N1—C1—C2	−0.1 (2)	C13—N4—C11—C12	−0.1 (2)
N1—C1—C2—N2	0.2 (2)	N4—C11—C12—N5	−0.3 (2)
N1—C1—C2—N3	−174.74 (17)	N4—C11—C12—N6	−176.66 (17)
C3—N2—C2—C1	−0.28 (18)	C13—N5—C12—C11	0.60 (19)
C5—N2—C2—C1	173.68 (16)	C15—N5—C12—C11	176.68 (17)
C3—N2—C2—N3	174.81 (16)	C13—N5—C12—N6	176.92 (17)
C5—N2—C2—N3	−11.2 (3)	C15—N5—C12—N6	−7.0 (3)
O2—N3—C2—C1	171.23 (18)	O6—N6—C12—C11	176.4 (2)
O1—N3—C2—C1	−7.5 (3)	O5—N6—C12—C11	−3.6 (3)
O2—N3—C2—N2	−2.9 (3)	O6—N6—C12—N5	0.8 (3)
O1—N3—C2—N2	178.41 (16)	O5—N6—C12—N5	−179.24 (18)
C1—N1—C3—N2	−0.1 (2)	C11—N4—C13—N5	0.5 (2)
C1—N1—C3—C4	178.22 (18)	C11—N4—C13—C14	−179.61 (19)
C2—N2—C3—N1	0.24 (19)	C12—N5—C13—N4	−0.7 (2)
C5—N2—C3—N1	−174.04 (15)	C15—N5—C13—N4	−176.92 (17)
C2—N2—C3—C4	−178.06 (17)	C12—N5—C13—C14	179.41 (19)
C5—N2—C3—C4	7.7 (3)	C15—N5—C13—C14	3.2 (3)
C3—N2—C5—C6	109.45 (19)	C13—N5—C15—C16	105.9 (2)
C2—N2—C5—C6	−63.4 (2)	C12—N5—C15—C16	−69.4 (3)
N2—C5—C6—S1	−55.76 (19)	N5—C15—C16—S2	−68.9 (2)
O4—S1—C6—C5	−53.86 (16)	O8—S2—C16—C15	−60.75 (17)
O3—S1—C6—C5	74.51 (16)	O7—S2—C16—C15	67.64 (17)
C7—S1—C6—C5	−169.61 (14)	C17—S2—C16—C15	−176.70 (15)
O4—S1—C7—C8	72.3 (2)	O8—S2—C17—C18	71.3 (2)
O3—S1—C7—C8	−56.8 (2)	O7—S2—C17—C18	−58.4 (2)
C6—S1—C7—C8	−172.42 (17)	C16—S2—C17—C18	−173.24 (19)

### 1-[2-(Ethanesulfonyl)ethyl]-2-methyl-5-nitro-1H-imidazole hemihydrate (TNZ-hemihydrate) Hydrogen-bond geometry (Å, º)

**Table:**

*D*—H···*A*	*D*—H	H···*A*	*D*···*A*	*D*—H···*A*
O1*WA*—H1*WB*···N1	0.85	2.09	2.908 (5)	162
O1*WB*—H1*WC*···N1	0.85	2.06	2.882 (5)	163
O1*WA*—H1*WA*···O1*WA*^i^	0.85	1.44	2.180 (10)	143
O1*WB*—H1*WD*···O8^i^	0.85	2.56	3.370 (5)	158
C1—H1···N4^ii^	0.93	2.38	3.310 (3)	175
C6—H6*B*···O2	0.97	2.42	3.063 (2)	124
C14—H14*B*···O1*WA*^i^	0.96	2.49	3.348 (6)	149
C15—H15*A*···O1*WB*^i^	0.97	2.46	3.41 (4)	168
C16—H16*B*···O6	0.97	2.50	3.100 (3)	120
C16—H16*A*···O7^ii^	0.97	2.37	3.183 (2)	141

Symmetry codes: (i) −*x*+1, −*y*+1, −*z*; (ii) *x*+1, *y*, *z*.

## References

[bb1] Alfaro-Fuentes, I., López-Sandoval, H., Mijangos, E., Duarte-Hernández, A. M., Rodriguez-López, G., Bernal-Uruchurtu, M. I., Contreras, R., Flores-Parra, A. & Barba-Behrens, N. (2014). *Polyhedron* **67**, 373–380.

[bb2] Allen, F. H. & Bruno, I. J. (2010). *Acta Cryst.* B**66**, 380–386. 10.1107/S0108768110012048 20484809

[bb3] Ang, C. W., Jarrad, A. M., Cooper, M. A. & Blaskovich, A. T. (2017). *J. Med. Chem.* **60**, 7636–7657. 10.1021/acs.jmedchem.7b00143 28463485

[bb4] Becke, A. D. (1993). *J. Chem. Phys.* **98**, 5648–5652.

[bb5] Ben, A. & Chęcińska, L. (2025). *Acta Cryst.* E**81**, 1018–1022. 10.1107/S2056989025008886 PMC12589828 41209644

[bb6] Bernstein, J., Davis, R. E., Shimoni, L. & Chang, N. L. (1995). *Angew. Chem. Int. Ed. Engl.* **34**, 1555–1573.

[bb7] Chasseaud, L. F., Henrick, K., Matthews, R. W., Scott, P. W. & Wood, S. G. (1984). *J. Chem. Soc. Chem. Commun.* pp. 491–492.

[bb8] Crowell, A. L., Sanders-Lewis, K. A. & Secor, W. E. (2003). *Antimicrob. Agents Chemother.* **47**, 1407–1409. 10.1128/AAC.47.4.1407-1409.2003 PMC152533 12654679

[bb9] Etter, M. C. (1990). *Acc. Chem. Res.* **23**, 120–126.

[bb10] Etter, M. C., MacDonald, J. C. & Bernstein, J. (1990). *Acta Cryst.* B**46**, 256–262. 10.1107/s0108768189012929 2344397

[bb11] Fandiño, O. E., Reviglio, L., Linck, Y. G., Monti, G. A., Marcos Valdez, M. M., Faudone, S. N., Caira, M. R. & Sperandeo, N. R. (2020). *Cryst. Growth Des.* **20**, 2930–2942.

[bb12] Frisch, M. J., Trucks, G. W., Schlegel, H. B., Scuseria, G. E., Robb, M. A., Cheeseman, J. R., Scalmani, G., Barone, V., Mennucci, B., Petersson, G. A., Nakatsuji, H., Caricato, M., Li, X., Hratchian, H. P., Izmaylov, A. F., Bloino, J., Zheng, G., Sonnenberg, J. L., Hada, M., Ehara, M., Toyota, K., Fukuda, R., Hasegawa, J., Ishida, M., Nakajima, T., Honda, Y., Kitao, O., Nakai, H., Vreven, T., Montgomery, J. A. Jr, Peralta, J. E., Ogliaro, F., Bearpark, M., Heyd, J. J., Brothers, E., Kudin, K. N., Staroverov, V. N., Keith, T., Kobayashi, R., Normand, J., Raghavachari, K., Rendell, A., Burant, J. C., Iyengar, S. S., Tomasi, J., Cossi, M., Rega, N., Millam, J. M., Klene, M., Knox, J. E., Cross, J. B., Bakken, V., Adamo, C., Jaramillo, J., Gomperts, R., Stratmann, R. E., Yazyev, O., Austin, A. J., Cammi, R., Pomelli, C., Ochterski, J. W., Martin, R. L., Morokuma, K., Zakrzewski, V. G., Voth, G. A., Salvador, P., Dannenberg, J. J., Dapprich, S., Daniels, A. D., Farkas, O., Foresman, J. B., Ortiz, J. V., Cioslowski, J. & Fox, D. J. (2013). *GAUSSIAN09*. Revision D. 01. Gaussian Inc., Wallingford CT, USA. https://gaussian.com/.

[bb13] Fung, H. B. & Doan, T. L. (2005). *Clin. Ther.* **27**, 1859–1884. 10.1016/j.clinthera.2005.12.012 16507373

[bb14] Gardner, T. B. & Hill, D. R. (2001). *Clin. Microbiol. Rev.* **14**, 114–128. 10.1128/CMR.14.1.114-128.2001 PMC88965 11148005

[bb15] Grimme, S., Ehrlich, S. & Goerigk, L. (2011). *J. Comput. Chem.* **32**, 1456–1465. 10.1002/jcc.21759 21370243

[bb16] Groom, C. R., Bruno, I. J., Lightfoot, M. P. & Ward, S. C. (2016). *Acta Cryst.* B**72**, 171–179. 10.1107/S2052520616003954 PMC4822653 27048719

[bb17] Johnson, E. R. & Becke, A. D. (2006). *J. Chem. Phys.* **124**, 174104. 10.1063/1.2190220 16689564

[bb18] Li, N., Chen, R., Zhang, M., Wu, T. & Liu, K. (2023). *CSD Communication* (refcode NIJCOC). CCDC, Cambridge, England.

[bb19] Mackenzie, C. F., Spackman, P. R., Jayatilaka, D. & Spackman, M. A. (2017). *IUCrJ* **4**, 575–587. 10.1107/S205225251700848X PMC5600021 28932404

[bb20] Macrae, C. F., Sovago, I., Cottrell, S. J., Galek, P. T. A., McCabe, P., Pidcock, E., Platings, M., Shields, G. P., Stevens, J. S., Towler, M. & Wood, P. A. (2020). *J. Appl. Cryst.* **53**, 226–235. 10.1107/S1600576719014092 PMC6998782 32047413

[bb21] Nakamura, S. (1955). *Pharm. Bull.* **3**, 379–383. 10.1248/cpb1953.3.379 13289298

[bb22] Rigaku OD (2023). *CrysAlis PRO*. Rigaku Oxford Diffraction, Yarnton, England.

[bb23] Rigaku OD (2025). *CrysAlis PRO*. Rigaku Oxford Diffraction, Yarnton, England.

[bb24] Sawyer, P. R., Brogden, R. N., Pinder, R. M., Speight, T. M. & Avery, G. S. (1976). *Drugs* **11**, 423–440. 10.2165/00003495-197611060-00003 954609

[bb25] Sheldrick, G. M. (2015*a*). *Acta Cryst.* A**71**, 3–8.

[bb26] Sheldrick, G. M. (2015*b*). *Acta Cryst.* C**71**, 3–8.

[bb27] Spackman, M. A. & Jayatilaka, D. (2009). *CrystEngComm* **11**, 19–32.

[bb28] Spackman, M. A. & McKinnon, J. J. (2002). *CrystEngComm* **4**, 378–392.

[bb29] Spackman, P. R., Turner, M. J., McKinnon, J. J., Wolff, S. K., Grimwood, D. J., Jayatilaka, D. & Spackman, M. A. (2021). *J. Appl. Cryst.* **54**, 1006–1011. 10.1107/S1600576721002910 PMC8202033 34188619

[bb30] Spek, A. L. (2020). *Acta Cryst.* E**76**, 1–11. 10.1107/S2056989019016244 PMC6944088 31921444

[bb31] Turner, M. J., Grabowsky, S., Jayatilaka, D. & Spackman, M. A. (2014). *J. Phys. Chem. Lett.* **5**, 4249–4255. 10.1021/jz502271c 26273970

[bb32] Turner, M. J., Thomas, S. P., Shi, M. W., Jayatilaka, D. & Spackman, M. A. (2015). *Chem. Commun.* **51**, 3735–3738. 10.1039/c4cc09074h 25525647

[bb33] Westrip, S. P. (2010). *J. Appl. Cryst.* **43**, 920–925.

[bb34] Wood, B. A. & Monro, A. M. (1975). *Br. J. Vener. Dis*. **51**, 51–53. https://doi.org/10.1136/sti.51.1.51 10.1136/sti.51.1.51 PMC1045111 1092424

[bb35] Zheng, K., Xie, C., Li, X., Wu, W., Li, A., Qian, S. & Pang, Q. (2020). *Acta Cryst.* C**76**, 389–397. 10.1107/S2053229620004180 32367818

