# Supplementary material for: Crystal structures and conformational features of new forms of tinidazole
Source: Acta Crystallogr E Crystallogr Commun. 2025 Nov 21;81(Pt 12):1170–7. doi: 10.1107/S2056989025010126 (PMC12810273; doi:10.1107/S2056989025010126)
Supplement: Supplementary file 8 [file e-81-01170-sup8.docx]

**SUPPORTING INFORMATION**

Crystal structures and conformational features
of new forms of tinidazole

Valeryia Hushcha,^a,b^ Justyna Dominikowska,^b^ Lilianna Chęcińska^b*^

^a^ University of Lodz Doctoral School of Exact and Natural Sciences, Narutowicza 68, 90-136 Łódź, Poland

^b^ University of Lodz, Faculty of Chemistry, Pomorska 163/165, 90-236 Łódź, Poland

* Corresponding author

Email address: [lilianna.checinska@chemia.uni.lodz.pl](mailto:lilianna.checinska@chemia.uni.lodz.pl); <https://orcid.org/0000-0002-3546-920X>

**Table of contents**

|  | **Figures and tables** | **Page** |
| --- | --- | --- |
| Table S1 | The results of conformational analysis of tinidazole molecule: total electronic energy (E_h_), relative energy of conformers (Δ*E*), torsion angle and imaginary frequencies | 2-3 |
| Figure S2 | Relative energy values (Δ*E*), calculated solely for conformers of TNZ in the crystal structures | 3 |
| Figure S3 | Geometric parameters of aromatic π-π interactions for TNZ-hemihydrate and TNZ-triclinic | 4 |
| Table S4 | Interaction energies for TNZ-monoclinic | 4 |
| Table S5 | Interaction energies for TNZ-triclinic | 5 |
| Figure S1 | Comparison of Hirshfeld surfaces and the corresponding 2D fingerprint plots of the most dominant contacts for TNZ-monoclinic and TNZ-triclinic (molecules: 1 and 2) | 6 |
| Figure S2 | Comparison of Hirshfeld surfaces and the corresponding 2D fingerprint plots of the most dominant contacts for TNZ-hemihydrate (molecules: 1 and 2 and disorder components of water: A and B) | 7 |
| Table S6-S59 | Final Cartesian coordinates for the optimized conformers of TNZ | 8-34 |
| Table S60-S64 | Final Cartesian coordinates for the TNZ molecules taken from the crystal structures of TNZ-monoclinic, TNZ-triclinic and TNZ-hemihydrate (single-point calculations) | 35-37 |

**Table S1**. The results of conformational analysis of tinidazole molecule: total electronic energy (*E*_h_ in au), relative energy of conformers (Δ*E* in kJ mol^-1^), torsion angle (°) and imaginary frequencies (cm^-1^)

| Name/number of conformation | *E_h_* | Δ*E* | Torsion angle  N2−C5−C6−S1 | Imaginary frequency |
| --- | --- | --- | --- | --- |
| 43 | -1176.1631156 | 0.000 | -63.84 |  |
| 187 | -1176.1631156 | 0.000 | 63.80 |  |
| 15 | -1176.1615018 | 4.238 | 64.68 |  |
| 16 | -1176.1615018 | 4.238 | -64.68 |  |
| 31 | -1176.1599960 | 8.191 | -61.64 |  |
| 157 | -1176.1598704 | 8.521 | 77.49 |  |
| 29 | -1176.1597280 | 8.895 | -76.87 |  |
| 12 | -1176.1596996 | 8.970 | 61.65 |  |
| 13 | -1176.1596996 | 8.970 | -61.66 |  |
| 30 | -1176.1594393 | 9.653 | -77.29 |  |
| 46 | -1176.1593809 | 9.807 | 62.35 |  |
| 56 | -1176.1593150 | 9.980 | 62.13 |  |
| 191 | -1176.1593150 | 9.980 | -62.12 |  |
| 1 | -1176.1591072 | 10.525 | 62.34 |  |
| 2 | -1176.1591071 | 10.526 | -62.34 |  |
| 138 | -1176.1586569 | 11.708 | -176.91 | -10.1774*i* |
| 192 | -1176.1586568 | 11.708 | 176.92 | -10.1233*i* |
| 74 | -1176.1573346 | 15.180 | 173.32 |  |
| 123 | -1176.1573346 | 15.180 | -173.31 |  |
| 4 | -1176.1569820 | 16.106 | -174.65 |  |
| 5 | -1176.1569820 | 16.106 | 174.64 |  |
| 143 | -1176.1558313 | 19.127 | -64.83 |  |
| 54 | -1176.1558312 | 19.127 | 64.81 |  |
| 50 | -1176.1556677 | 19.557 | 144.45 | -4.7271*i* |
| 79 | -1176.1556677 | 19.557 | -144.44 | -5.1011*i* |
| 35 | -1176.1556025 | 19.728 | 64.26 |  |
| 93 | -1176.1556025 | 19.728 | -64.25 |  |
| 57 | -1176.1549601 | 21.415 | 75.89 | -5.4492*i* |
| 111 | -1176.1549601 | 21.415 | -75.90 | -5.3270*i* |
| 37 | -1176.1545366 | 22.527 | 74.12 |  |
| 68 | -1176.1545366 | 22.527 | -74.11 |  |
| 65 | -1176.1531321 | 26.215 | -62.46 |  |
| 55 | -1176.1531320 | 26.215 | 62.46 |  |
| 36 | -1176.1529755 | 26.626 | 62.55 |  |
| 125 | -1176.1529754 | 26.626 | -62.53 |  |
| 173 | -1176.1529632 | 26.658 | 62.42 |  |
| 17 | -1176.1525613 | 27.713 | 167.57 |  |
| 24 | -1176.1525613 | 27.713 | -167.60 |  |
| 58 | -1176.1520640 | 29.019 | 57.10 |  |
| 144 | -1176.1520637 | 29.020 | -67.02 |  |
| 48 | -1176.1517951 | 29.725 | 170.14 |  |
| 73 | -1176.1517951 | 29.725 | -170.15 |  |
| 85 | -1176.1513796 | 30.816 | 177.20 |  |
| 88 | -1176.1513796 | 30.816 | -177.20 |  |
| 200 | -1176.1505342 | 33.036 | 47.50 |  |
| 185 | -1176.1504050 | 33.375 | 47.64 |  |
| 199 | -1176.1500551 | 34.294 | -47.57 |  |
| 148 | -1176.1500550 | 34.295 | 47.57 |  |
| 26 | -1176.1497281 | 35.153 | 76.89 |  |
| 120 | -1176.1497280 | 35.153 | -76.86 |  |
| 25 | -1176.1494394 | 35.911 | 77.29 |  |
| 121 | -1176.1494393 | 35.911 | -77.29 |  |
| 160 | -1176.1491393 | 36.699 | -78.01 |  |
| 167 | -1176.1491392 | 36.699 | 78.02 |  |
| TNZ-mono-sp | -1176.1489602 | 37.169 | -170.053 |  |
| TNZ-tri-mol1-sp | -1176.1494888 | 35.781 | -56.062 |  |
| TNZ-tri-mol2-sp | -1176.1484969 | 38.386 | -70.348 |  |
| TNZ-hemi-mol1-sp | -1176.1495396 | 35.648 | -55.766 |  |
| TNZ-hemi-mol2-sp | -1176.1489482 | 37.201 | -68.905 |  |

sp – single point calculations

**Table S2**. Relative energy values (Δ*E* in kJ mol^-1^), calculated solely for conformers of TNZ in the crystal structures

| TNZ-mono-sp | 1.521 |
| --- | --- |
| TNZ-tri-mol1-sp | 0.133 |
| TNZ-tri-mol2-sp | 2.738 |
| TNZ-hemi-mol1-sp | 0.000 |
| TNZ-hemi-mol2-sp | 1.553 |

sp – single point calculations

**Table S3**. Geometric parameters (Å, °) of aromatic π-π interactions for TNZ-triclinic and TNZ-hemihydrate

| Structure | Interaction | *Cg*(I)⋅⋅⋅*Cg*(J) | α | *Cg*(I)_perp_ | *Cg*(J)_perp_ | Slippage |
| --- | --- | --- | --- | --- | --- | --- |
| TNZ-triclinic | *Cg*(1)···*Cg*(2) | 3.5428(12) | 2.30(12) | 3.4013(9) | 3.3766(9) | 1.072 |
| TNZ-hemihydrate | *Cg*(1)···*Cg*(2) | 3.4929(12) | 1.79(12) | 3.3505(8) | 3.3550(9) | 0.972 |

*Cg*(1) and *Cg*(2) – a centre-of-gravity of imidazole ring.

*Cg*(I)⋅⋅⋅*Cg*(J) – distance between ring centroids; α - dihedral angle between planes I and J; *Cg*(I)_perp_ and *Cg*(J)_perp_ - (interplanar spacing) perpendicular distance of *Cg*(I) on ring J and *Cg*(J) on ring I, respectively; slippage - distance between *Cg*(I) and perpendicular projection of *Cg*(J) on ring I.

**Table S4**. Interaction energies (kJ mol^−1^) for TNZ-monoclinic

| *N* | Symmetry | *R* | *kE*_ele_ | *kE*_pol_ | *kE*_dis_ | *kE*_rep_ | *E*_tot_ |
| --- | --- | --- | --- | --- | --- | --- | --- |
| 1 | −*x*, −*y*, −*z* | 6.75 | -47.2 | -8.7 | -37.4 | 18.8 | -64.6 |
| 2 | *x*+1/2, −*y*+1/2, *z*+1/2 | 9.78 | -9.6 | -1.8 | -11.8 | 6.1 | -17.1 |
| 2 | *x*, *y*, *z* | 5.52 | -11.2 | -4.0 | -27.5 | 12.1 | -30.6 |
| 1 | −*x*, −*y*, −*z* | 5.48 | -16.2 | -6.4 | -28.0 | 12.1 | -38.4 |
| 2 | *x*+1/2, −*y*+1/2, *z*+1/2 | 10.52 | -1.5 | -0.3 | -5.0 | 1.4 | -4.6 |
| 2 | −*x*+1/2, *y*+1/2, −*z*+1/2 | 9.98 | -1.7 | -0.8 | -7.5 | 2.6 | -6.6 |
| 2 | −*x*+1/2, *y*+1/2, −*z*+1/2 | 7.87 | -13.7 | -1.4 | -10.1 | 4.6 | -13.8 |
| 1 | −*x*, −*y*, −*z* | 7.07 | -5.0 | -1.3 | -24.9 | 8.2 | -22.9 |
| 1 | −*x*, −*y*, −*z* | 9.77 | -12.8 | -1.3 | -6.8 | 9.0 | -11.9 |

*N* is the number of molecular pairs. *R* is the distance (Å, °) between molecular centroids*. E*_tot_ is the total energy and its individual components: *E*_ele_ is electrostatic (*k*=1.057), *E*_pol_ is polarization (*k*=0.740), *E*_dis_ is dispersion (*k*=0.871), *E*_rep_ is repulsion (*k*=0.618).

**Table S5**. Interaction energies (kJ mol^−1^) for TNZ-triclinic

| *N* | Symmetry | *R* | *kE*_ele_ | *kE*_pol_ | *kE*_dis_ | *kE*_rep_ | *E*_tot_ |
| --- | --- | --- | --- | --- | --- | --- | --- |
| 1 | −*x*, −*y*, −*z* | 6.84 | -26.5 | -4.2 | -16.2 | 10.3 | -36.6 |
| 1 | − | 8.54 | -9.4 | -1.8 | -9.3 | 4.3 | -16.3 |
| 2 | *x*, *y*, *z* | 5.72 | -5.2 | -2.8 | -21.9 | 6.8 | -23.3 |
| 1 | − | 7.08 | -7.5 | -1.9 | -32.1 | 11.0 | -30.5 |
| 1 | − | 7.22 | -16.1 | -3.6 | -20.9 | 9.1 | -31.5 |
| 1 | − | 10.66 | 1.4 | -0.2 | -4.8 | 1.5 | -2.1 |
| 1 | −*x*, −*y*, −*z* | 11.41 | 5.9 | -1.0 | -5.8 | 1.7 | 0.8 |
| 1 | −*x*, −*y*, −*z* | 6.80 | -17.8 | -4.0 | -13.3 | 4.8 | -30.3 |
| 1 | − | 8.80 | -8.6 | -2.2 | -9.2 | 5.0 | -15.0 |
| 1 | −*x*, −*y*, −*z* | 9.90 | -1.4 | -0.1 | -2.9 | 0.6 | -3.9 |
| 1 | − | 6.79 | -18.5 | -3.3 | -26.8 | 11.1 | -37.6 |
| 1 | − | 8.82 | -20.4 | -3.3 | -9.5 | 15.5 | -17.7 |
| 1 | − | 10.03 | -1.8 | -0.7 | -3.7 | 0.4 | -5.9 |
| 1 | −*x*, −*y*, −*z* | 7.27 | -5.6 | -1.6 | -17.9 | 8.8 | -16.3 |
| 2 | *x*, *y*, *z* | 5.72 | -17.3 | -4.0 | -21.8 | 14.6 | -28.5 |
| 1 | −*x*, −*y*, −*z* | 6.72 | -22.5 | -3.8 | -20.8 | 14.8 | -32.5 |
| 1 | −*x*, −*y*, −*z* | 13.48 | 1.5 | -0.2 | -1.6 | 0 | -0.3 |

*N* is the number of molecular pairs. *R* is the distance (Å, °) between molecular centroids*. E*_tot_ is the total energy and its individual components: *E*_ele_ is electrostatic (*k*=1.057), *E*_pol_ is polarization (*k*=0.740), *E*_dis_ is dispersion (*k*=0.871), *E*_rep_ is repulsion (*k*=0.618).

| All | H···H | O···H/H···O | N···H/H···N |
| --- | --- | --- | --- |
| TNZ-monoclinic | | | |
| 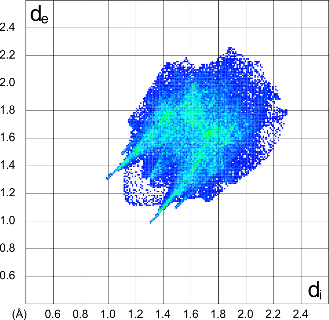 | 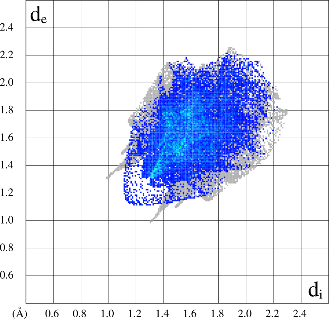 | 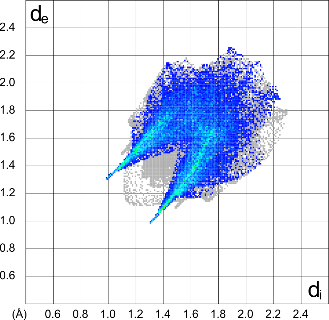 | 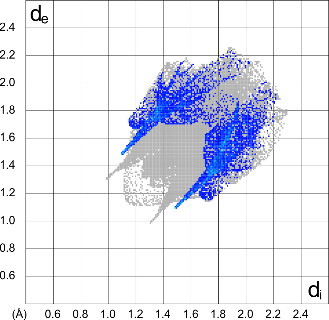 |
| 100% | 33.8% | 43.7% | 11.3% |
| TNZ-triclinic (molecule 1) | | | |
| 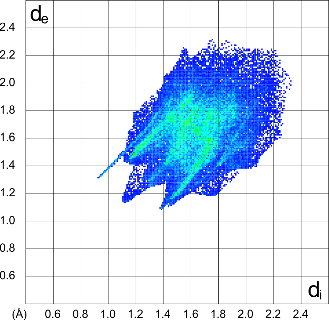 | 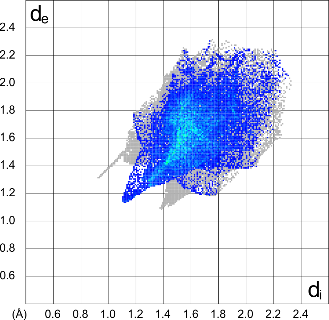 | 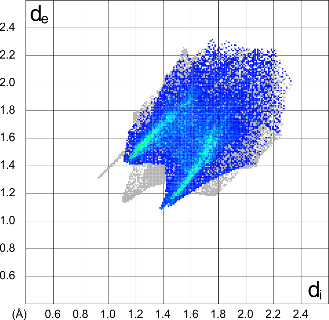 | 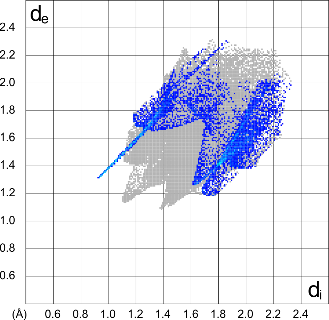 |
| 100% | 35.4% | 44.0% | 10.5% |
| TNZ-triclinic (molecule 2) | | | |
| 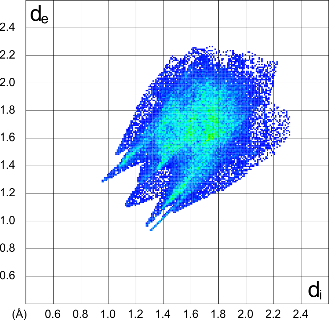 | 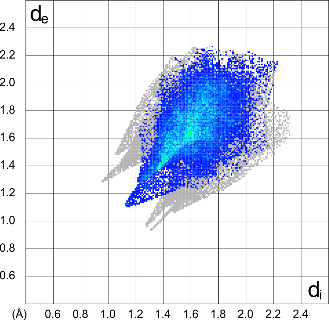 | 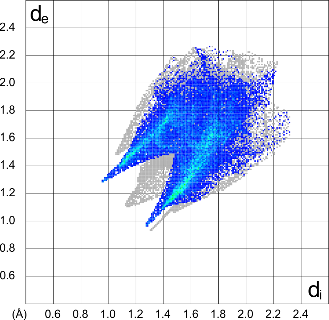 | 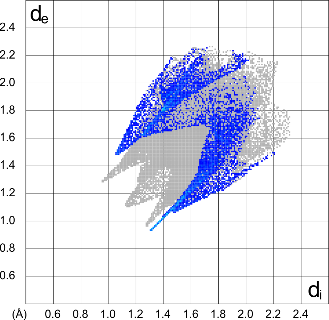 |
| 100% | 40.7% | 41.1% | 10.4% |

**Figure S1.** Comparison of Hirshfeld surfaces and the corresponding two-dimensional fingerprint plots of the most dominant contacts for TNZ-monoclinic and TNZ-triclinic (molecules: 1 and 2). The *d*_i_ and *d*_e_ values are the closest internal and external distances (in Å) from given points on the Hirshfeld surface.

| All | H···H | O···H/H···O | N···H/H···N |
| --- | --- | --- | --- |
| TNZ-hemihydrate (TNZ molecule 1 and water molecule A) | | | |
| 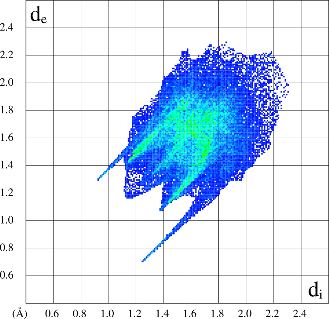 | 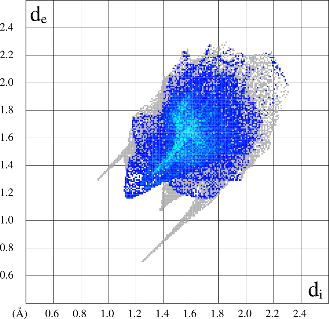 | 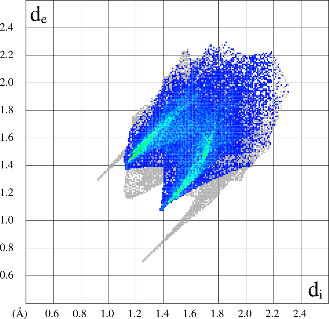 | 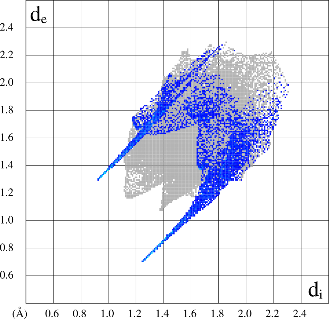 |
| 100% | 33.5% | 46.8% | 9.7% |
| TNZ-hemihydrate (TNZ molecule 2 and water molecule A) | | | |
| 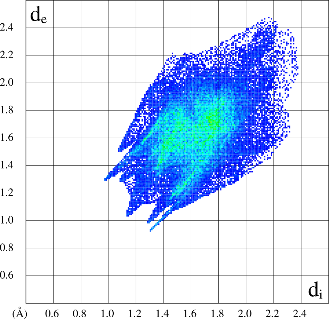 | 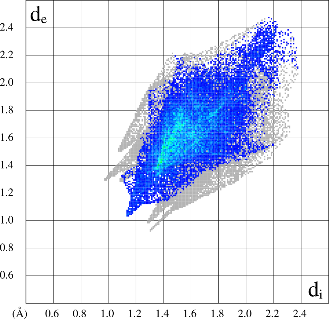 | 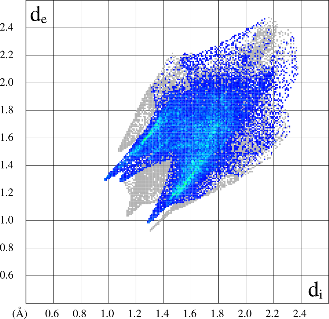 | 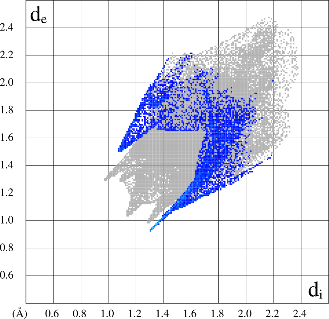 |
| 100% | 41.0% | 42.8% | 8.3% |
| TNZ-hemihydrate (TNZ molecule 1 and water molecule B) | | | |
| 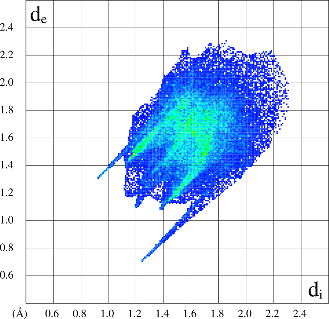 | 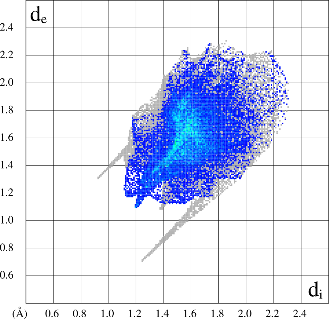 | 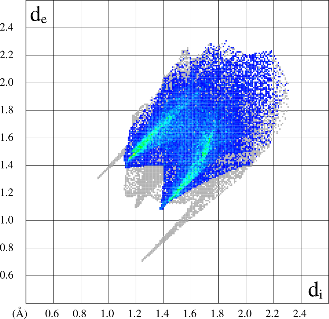 | 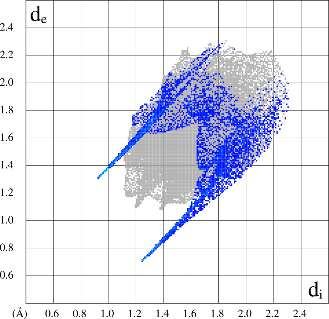 |
| 100% | 34.5% | 45.8% | 9.6% |
| TNZ-hemihydrate (TNZ molecule 2 and water molecule B) | | | |
| 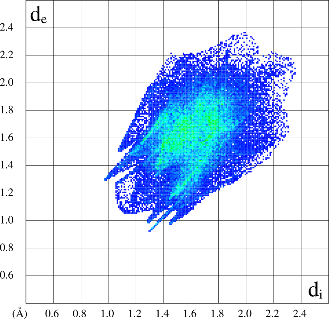 | 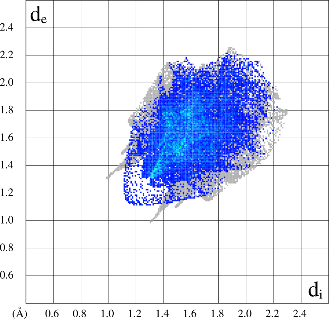 | 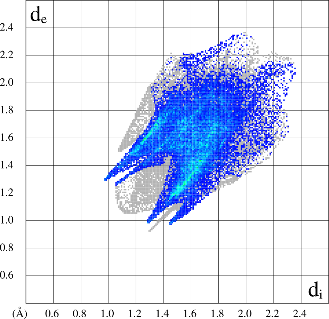 | 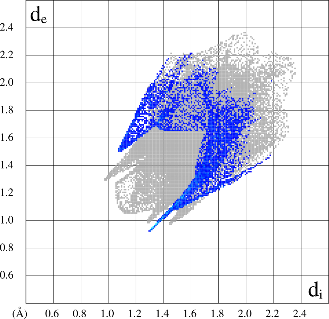 |
| 100% | 40.7% | 43.0% | 8.5% |

**Figure S2.** Comparison of Hirshfeld surfaces and the corresponding two-dimensional fingerprint plots of the most dominant contacts for TNZ-hemihydrate (molecules: 1 and 2 and disorder components of water: A and B). The *d*_i_ and *d*_e_ values are the closest internal and external distances (in Å) from given points on the Hirshfeld surface.

**Table S6**. Final Cartesian coordinates (X, Y, Z in Å) for the optimized TNZ molecule (conformer no. 43)

**Number Atom X Y Z**

1 S 2.173179 -0.230802 -0.033343

2 O -1.801440 -1.657190 -1.547560

3 O -2.982133 -1.947713 0.262437

4 O 2.499488 1.202172 -0.104843

5 O 3.229793 -1.240431 -0.135845

6 N -1.765399 1.892287 1.203196

7 N -0.907172 0.828069 -0.563787

8 N -2.232013 -1.288335 -0.448701

9 C -2.349274 0.670947 1.119691

10 H -3.106610 0.329446 1.805954

11 C -1.841554 -0.012405 0.036932

12 C -0.904955 1.973291 0.189728

13 C -0.056889 3.161613 -0.094395

14 H -0.259625 3.902479 0.676458

15 H -0.299388 3.597332 -1.068294

16 H 1.003902 2.903380 -0.083753

17 C 0.004119 0.532555 -1.673871

18 H 0.543522 1.451674 -1.888871

19 H -0.583635 0.249218 -2.545042

20 C 0.994915 -0.586131 -1.375892

21 H 1.650885 -0.737352 -2.235257

22 H 0.505051 -1.532242 -1.157569

23 C 1.289735 -0.514175 1.529398

24 H 2.040698 -0.221023 2.266872

25 H 0.473716 0.205372 1.575438

26 C 0.840470 -1.955403 1.720557

27 H 0.071373 -2.246469 1.003311

28 H 0.412297 -2.070203 2.718252

29 H 1.683969 -2.639383 1.624655

**Table S7**. Final Cartesian coordinates (X, Y, Z in Å) for the optimized TNZ molecule (conformer no. 187)

**Number Atom X Y Z**

1 S -2.173173 -0.231244 -0.032888

2 O 1.800405 -1.658007 -1.546509

3 O 2.983828 -1.946402 0.261987

4 O -3.229734 -1.241058 -0.134614

5 O -2.499837 1.201679 -0.104480

6 N 1.765413 1.893258 1.202087

7 N 0.906445 0.827834 -0.563814

8 N 2.232283 -1.287999 -0.448530

9 C 2.349704 0.672156 1.118797

10 H 3.107533 0.331180 1.804793

11 C 1.841841 -0.011874 0.036574

12 C 0.904067 1.973339 0.189315

13 C 0.055595 3.161399 -0.094634

14 H -1.005195 2.903268 -0.082900

15 H 0.297022 3.596393 -1.069157

16 H 0.259113 3.902877 0.675402

17 C -0.004695 0.532146 -1.673973

18 H 0.583124 0.248647 -2.545054

19 H -0.544062 1.451228 -1.889137

20 C -0.995701 -0.586399 -1.375926

21 H -0.505986 -1.532693 -1.158097

22 H -1.652019 -0.737230 -2.235109

23 C -1.289120 -0.514373 1.529574

24 H -0.473935 0.206083 1.576100

25 H -2.040493 -0.222695 2.267227

26 C -0.838398 -1.955275 1.720046

27 H -1.681605 -2.639790 1.625576

28 H -0.408511 -2.069615 2.717057

29 H -0.070260 -2.245821 1.001592

**Table S8**. Final Cartesian coordinates (X, Y, Z in Å) for the optimized TNZ molecule (conformer no. 15)

**Number Atom X Y Z**

1 S -2.064717 -0.481860 -0.242602

2 O 2.103290 -2.013133 -0.863948

3 O 3.223229 -1.704898 0.980828

4 O -3.090780 -1.527486 -0.233759

5 O -2.401120 0.878696 -0.693955

6 N 1.670727 2.122373 0.844273

7 N 1.044301 0.592731 -0.658990

8 N 2.457757 -1.327032 0.102110

9 C 2.319726 0.964500 1.122487

10 H 3.019346 0.859804 1.935309

11 C 1.956288 -0.001997 0.211167

12 C 0.915026 1.887619 -0.226681

13 C 0.059154 2.910575 -0.885424

14 H -0.984339 2.592787 -0.924713

15 H 0.399981 3.111845 -1.905713

16 H 0.140716 3.828878 -0.307220

17 C 0.282297 -0.024436 -1.748726

18 H 0.979123 -0.481434 -2.449100

19 H -0.252935 0.778880 -2.249052

20 C -0.705725 -1.090628 -1.291698

21 H -0.221540 -1.913632 -0.769632

22 H -1.231575 -1.499922 -2.156556

23 C -1.370662 -0.367382 1.434657

24 H -0.984738 -1.360668 1.669944

25 H -0.548302 0.345499 1.393942

26 C -2.457060 0.073716 2.409056

27 H -2.846524 1.054745 2.134016

28 H -2.033556 0.139460 3.412867

29 H -3.280895 -0.640209 2.423441

**Table S9**. Final Cartesian coordinates (X, Y, Z in Å) for the optimized TNZ molecule (conformer no. 16)

**Number Atom X Y Z**

1 S 2.064728 -0.481830 -0.242607

2 O -2.103203 -2.013175 -0.863956

3 O -3.223253 -1.704937 0.980747

4 O 2.401050 0.878792 -0.693825

5 O 3.090857 -1.527394 -0.233866

6 N -1.670773 2.122336 0.844311

7 N -1.044287 0.592737 -0.658970

8 N -2.457727 -1.327070 0.102078

9 C -2.319738 0.964438 1.122502

10 H -3.019361 0.859710 1.935318

11 C -1.956278 -0.002028 0.211159

12 C -0.915062 1.887625 -0.226645

13 C -0.059237 2.910628 -0.885375

14 H -0.140873 3.828929 -0.307179

15 H -0.400043 3.111862 -1.905677

16 H 0.984281 2.592918 -0.924641

17 C -0.282273 -0.024373 -1.748733

18 H 0.252955 0.778977 -2.249012

19 H -0.979087 -0.481340 -2.449139

20 C 0.705763 -1.090570 -1.291751

21 H 1.231621 -1.499818 -2.156625

22 H 0.221588 -1.913609 -0.769730

23 C 1.370678 -0.367547 1.434667

24 H 0.548207 0.345206 1.394000

25 H 0.984919 -1.360907 1.669914

26 C 2.457020 0.073677 2.409072

27 H 3.280982 -0.640104 2.423387

28 H 2.033531 0.139273 3.412898

29 H 2.846305 1.054792 2.134088

**Table S10**. Final Cartesian coordinates (X, Y, Z in Å) for the optimized TNZ molecule (conformer no. 31)

**Number Atom X Y Z**

1 S -1.917941 0.349021 -0.128506

2 O 1.477268 -2.183058 1.141561

3 O 2.891519 -2.427267 -0.498904

4 O -1.199136 0.204899 -1.398885

5 O -2.246500 1.689135 0.383706

6 N 2.349460 1.628817 -1.078817

7 N 1.215200 0.551405 0.514990

8 N 2.135481 -1.746576 0.184459

9 C 2.703233 0.322038 -1.117320

10 H 3.422190 -0.075520 -1.814375

11 C 2.021632 -0.374580 -0.143920

12 C 1.454965 1.750067 -0.102562

13 C 0.808649 3.033324 0.282679

14 H 1.079717 3.326926 1.301885

15 H -0.280051 2.973101 0.221386

16 H 1.166640 3.798366 -0.403317

17 C 0.253429 0.322845 1.591798

18 H -0.062742 1.299991 1.951043

19 H 0.749212 -0.210062 2.400941

20 C -0.967141 -0.495232 1.171768

21 H -1.634699 -0.606535 2.028136

22 H -0.673561 -1.473916 0.800476

23 C -3.463690 -0.597336 -0.233345

24 H -3.909306 -0.565601 0.762385

25 H -4.073365 0.017182 -0.899622

26 C -3.263707 -2.007591 -0.770900

27 H -2.762110 -1.980526 -1.738536

28 H -4.234267 -2.491743 -0.896977

29 H -2.671014 -2.622393 -0.090762

**Table S11**. Final Cartesian coordinates (X, Y, Z in Å) for the optimized TNZ molecule (conformer no. 157)

**Number Atom X Y Z**

1 S -2.358172 -0.367630 -0.033525

2 O 1.787400 -2.178133 -0.885546

3 O 3.317627 -1.829442 0.627505

4 O -3.516738 -1.225763 -0.294858

5 O -2.417792 1.070249 -0.343411

6 N 1.986734 2.077363 0.556008

7 N 0.962186 0.515366 -0.670445

8 N 2.393477 -1.454718 -0.084791

9 C 2.611256 0.894871 0.771702

10 H 3.457619 0.783799 1.429295

11 C 2.006936 -0.093137 0.025008

12 C 1.007170 1.837696 -0.313775

13 C 0.102874 2.886419 -0.855191

14 H -0.945432 2.637474 -0.686053

15 H 0.254775 3.019386 -1.931080

16 H 0.348522 3.821725 -0.356033

17 C 0.007261 -0.095469 -1.597750

18 H 0.568818 -0.619175 -2.369469

19 H -0.551267 0.717114 -2.056461

20 C -0.959588 -1.094350 -0.970537

21 H -0.457172 -1.820277 -0.334828

22 H -1.470576 -1.643461 -1.762762

23 C -1.958426 -0.552250 1.730291

24 H -2.907091 -0.288472 2.202903

25 H -1.790181 -1.619764 1.883187

26 C -0.811864 0.319150 2.214630

27 H 0.145685 0.001805 1.803006

28 H -0.739657 0.249331 3.301895

29 H -0.977766 1.363645 1.951617

**Table S12**. Final Cartesian coordinates (X, Y, Z in Å) for the optimized TNZ molecule (conformer no. 29)

**Number Atom X Y Z**

1 S 1.903787 -0.443123 0.146504

2 O -1.035507 -2.261634 0.960593

3 O -2.026400 -2.615398 -0.944567

4 O 0.998975 -0.797463 -0.952147

5 O 2.389338 -1.456446 1.084017

6 N -2.665885 1.467466 -0.951585

7 N -1.313292 0.530942 0.559816

8 N -1.603210 -1.879606 -0.064041

9 C -2.655143 0.120229 -1.155833

10 H -3.235521 -0.364501 -1.922569

11 C -1.830904 -0.481951 -0.238648

12 C -1.854819 1.692800 0.070045

13 C -1.583341 3.040135 0.644550

14 H -0.548029 3.359796 0.492194

15 H -1.788009 3.073877 1.718662

16 H -2.236377 3.751318 0.142328

17 C -0.296415 0.458705 1.607907

18 H -0.288742 -0.552446 2.003852

19 H -0.597180 1.137971 2.407176

20 C 1.099175 0.869787 1.129333

21 H 1.751658 1.039355 1.986522

22 H 1.062616 1.766449 0.511622

23 C 3.350999 0.389922 -0.573275

24 H 3.895456 0.836222 0.261375

25 H 3.940931 -0.448404 -0.950349

26 C 2.991290 1.379513 -1.672115

27 H 2.400452 0.890594 -2.446553

28 H 3.903412 1.774015 -2.124826

29 H 2.419143 2.226796 -1.287774

**Table S13**. Final Cartesian coordinates (X, Y, Z in Å) for the optimized TNZ molecule (conformer no. 12)

**Number Atom X Y Z**

1 S -1.918297 0.348825 0.128180

2 O 1.478272 -2.182669 -1.141994

3 O 2.891839 -2.426884 0.499034

4 O -2.247589 1.688584 -0.384505

5 O -1.199259 0.205546 1.398548

6 N 2.349319 1.629215 1.078772

7 N 1.215026 0.551539 -0.514816

8 N 2.135999 -1.746208 -0.184565

9 C 2.703345 0.322499 1.117265

10 H 3.422470 -0.074903 1.814239

11 C 2.021817 -0.374239 0.143904

12 C 1.454548 1.750253 0.102771

13 C 0.807689 3.033315 -0.282162

14 H -0.280975 2.972829 -0.220412

15 H 1.078143 3.326954 -1.301530

16 H 1.165827 3.798494 0.403604

17 C 0.253377 0.322892 -1.591689

18 H 0.749302 -0.209789 -2.400895

19 H -0.063076 1.299997 -1.950831

20 C -0.967049 -0.495428 -1.171784

21 H -0.673402 -1.473990 -0.800188

22 H -1.634383 -0.607121 -2.028278

23 C -3.463479 -0.598427 0.233535

24 H -4.073454 0.016041 0.899591

25 H -3.909208 -0.567420 -0.762169

26 C -3.262550 -2.008273 0.771820

27 H -2.669948 -2.623199 0.091725

28 H -4.232818 -2.492782 0.898746

29 H -2.760427 -1.980308 1.739154

**Table S14**. Final Cartesian coordinates (X, Y, Z in Å) for the optimized TNZ molecule (conformer no. 13)

**Number Atom X Y Z**

1 S 1.918805 0.348477 0.127817

2 O -1.478528 -2.182710 -1.141123

3 O -2.894047 -2.425671 0.498375

4 O 1.199862 0.206660 1.398410

5 O 2.248906 1.687681 -0.385857

6 N -2.348881 1.629973 1.078676

7 N -1.214750 0.551670 -0.514596

8 N -2.137055 -1.745595 -0.184546

9 C -2.703841 0.323529 1.116913

10 H -3.423439 -0.073465 1.813631

11 C -2.022456 -0.373608 0.143708

12 C -1.453685 1.750482 0.102949

13 C -0.806177 3.033219 -0.281914

14 H -1.077264 3.327451 -1.300969

15 H 0.282500 2.972037 -0.221052

16 H -1.163308 3.798346 0.404418

17 C -0.253302 0.322471 -1.591585

18 H 0.063252 1.299361 -1.951200

19 H -0.749537 -0.210440 -2.400437

20 C 0.966964 -0.496045 -1.171508

21 H 1.634209 -0.608360 -2.027997

22 H 0.673011 -1.474337 -0.799484

23 C 3.463343 -0.599771 0.233701

24 H 3.908992 -0.569768 -0.762069

25 H 4.073841 0.014707 0.899266

26 C 3.261369 -2.009089 0.773013

27 H 2.759382 -1.980000 1.740383

28 H 4.231269 -2.494273 0.900137

29 H 2.668176 -2.624020 0.093441

**Table S15**. Final Cartesian coordinates (X, Y, Z in Å) for the optimized TNZ molecule (conformer no. 30)

**Number Atom X Y Z**

1 O -8.126006 1.482033 -0.116375

2 O -8.474350 -0.716470 0.113017

3 O -1.746823 -1.956251 0.092166

4 O -1.277608 0.237118 -0.096228

5 N -5.812303 -1.206452 -0.082936

6 C -6.227582 0.068685 0.004201

7 C -5.350926 1.138731 0.093694

8 H -5.728898 2.150292 0.175974

9 C -3.983969 0.901783 0.076909

10 H -3.279281 1.721763 0.139653

11 C -3.537898 -0.411729 -0.022037

12 C -4.494279 -1.421143 -0.092873

13 H -4.146788 -2.444640 -0.160641

14 C -7.731476 0.318202 0.004648

15 C -2.091588 -0.794729 -0.005691

16 H -0.296797 -0.020729 -0.007638

17 O 1.559699 1.855403 -0.113094

18 O 1.211356 -0.343100 0.116298

19 O 7.938882 -1.582880 0.095447

20 O 8.408098 0.610489 -0.092946

21 N 3.873403 -0.833082 -0.079655

22 C 3.458124 0.442055 0.007483

23 C 4.334780 1.512101 0.096975

24 H 3.956807 2.523662 0.179255

25 C 5.701737 1.275153 0.080190

26 H 6.406424 2.095133 0.142934

27 C 6.147808 -0.038358 -0.018756

28 C 5.191426 -1.047772 -0.089592

29 H 5.538918 -2.071269 -0.157360

30 C 1.954230 0.691572 0.007929

31 C 7.594118 -0.421358 -0.002410

32 H 9.388909 0.352641 -0.004357

**Table S16**. Final Cartesian coordinates (X, Y, Z in Å) for the optimized TNZ molecule (conformer no. 46)

**Number Atom X Y Z**

1 S 1.865420 -0.050878 0.008285

2 O -1.939973 -2.140023 0.931624

3 O -3.305179 -2.051536 -0.765293

4 O 2.338123 1.194652 0.636964

5 O 1.225008 -0.001614 -1.310440

6 N -2.126389 1.900109 -1.034630

7 N -1.244276 0.559204 0.518352

8 N -2.483794 -1.543749 -0.010722

9 C -2.671104 0.668587 -1.179861

10 H -3.406954 0.432256 -1.930523

11 C -2.149375 -0.186551 -0.234537

12 C -1.271568 1.818795 -0.019497

13 C -0.461631 2.961046 0.482533

14 H 0.606852 2.736423 0.471036

15 H -0.744928 3.231456 1.504879

16 H -0.659304 3.811762 -0.166522

17 C -0.384439 0.114806 1.613809

18 H -0.994107 -0.394271 2.357548

19 H 0.051681 1.006059 2.060521

20 C 0.725067 -0.842535 1.180723

21 H 0.317934 -1.732272 0.705687

22 H 1.316277 -1.124126 2.054138

23 C 3.263416 -1.210295 -0.074831

24 H 3.678432 -1.261728 0.933048

25 H 2.839644 -2.178011 -0.350523

26 C 4.279517 -0.714504 -1.097601

27 H 3.824651 -0.638517 -2.085687

28 H 5.113659 -1.416473 -1.150502

29 H 4.667520 0.264612 -0.813933

**Table S17**. Final Cartesian coordinates (X, Y, Z in Å) for the optimized TNZ molecule (conformer no. 56)

**Number Atom X Y Z**

1 S 1.825586 0.100009 -0.435200

2 O -1.667341 -2.271272 0.884412

3 O -3.264369 -2.160952 -0.594917

4 O 2.290447 1.350662 0.189190

5 O 1.000151 0.146529 -1.645256

6 N -2.382024 1.875961 -0.698333

7 N -1.194881 0.489582 0.588594

8 N -2.374859 -1.650762 0.075994

9 C -2.867086 0.622437 -0.866412

10 H -3.693367 0.390891 -1.517918

11 C -2.157854 -0.260990 -0.083213

12 C -1.380026 1.780875 0.170328

13 C -0.575109 2.937898 0.646691

14 H 0.490644 2.795701 0.455939

15 H -0.713447 3.104161 1.719943

16 H -0.923478 3.820200 0.113548

17 C -0.152418 0.030559 1.504424

18 H -0.611010 -0.559131 2.295971

19 H 0.302805 0.917857 1.939523

20 C 0.924281 -0.827938 0.843750

21 H 0.498535 -1.713013 0.376459

22 H 1.654064 -1.122178 1.599561

23 C 3.279775 -0.929006 -0.793597

24 H 2.892012 -1.918409 -1.044190

25 H 3.667202 -0.475912 -1.708848

26 C 4.309593 -0.940497 0.326388

27 H 4.609091 0.075990 0.581771

28 H 5.193975 -1.493565 0.003114

29 H 3.927913 -1.425973 1.226944

**Table S18**. Final Cartesian coordinates (X, Y, Z in Å) for the optimized TNZ molecule (conformer no. 191)

**Number Atom X Y Z**

1 S -1.825483 0.099988 -0.435230

2 O 3.264186 -2.160987 -0.594998

3 O 1.667421 -2.271207 0.884623

4 O -0.999992 0.146367 -1.645215

5 O -2.290361 1.350715 0.189018

6 N 2.381813 1.875937 -0.698504

7 N 1.194904 0.489605 0.588707

8 N 2.374776 -1.650750 0.076019

9 C 2.866825 0.622421 -0.866619

10 H 3.693009 0.390850 -1.518242

11 C 2.157725 -0.261005 -0.083276

12 C 1.379964 1.780867 0.170353

13 C 0.575170 2.937940 0.646815

14 H 0.713773 3.104279 1.720019

15 H -0.490622 2.795750 0.456289

16 H 0.923456 3.820184 0.113520

17 C 0.152408 0.030594 1.504524

18 H -0.302790 0.917905 1.939620

19 H 0.610973 -0.559115 2.296071

20 C -0.924283 -0.827891 0.843836

21 H -1.654095 -1.122079 1.599641

22 H -0.498549 -1.713010 0.376611

23 C -3.279691 -0.928971 -0.793619

24 H -3.667150 -0.475802 -1.708824

25 H -2.891962 -1.918363 -1.044308

26 C -4.309483 -0.940517 0.326395

27 H -3.927848 -1.426267 1.226824

28 H -5.193960 -1.493366 0.003013

29 H -4.608792 0.075969 0.581996

**Table S19**. Final Cartesian coordinates (X, Y, Z in Å) for the optimized TNZ molecule (conformer no. 1)

**Number Atom X Y Z**

1 S -1.865232 -0.050994 -0.008210

2 O 1.940122 -2.139872 -0.931849

3 O 3.304923 -2.051569 0.765399

4 O -2.337962 1.194632 -0.636679

5 O -1.224722 -0.001943 1.310486

6 N 2.126086 1.900083 1.034800

7 N 1.244310 0.559290 -0.518459

8 N 2.483697 -1.543710 0.010706

9 C 2.670721 0.668525 1.180106

10 H 3.406421 0.432168 1.930904

11 C 2.149161 -0.186544 0.234629

12 C 1.271482 1.818847 0.019480

13 C 0.461637 2.961107 -0.482650

14 H -0.606868 2.736527 -0.471017

15 H 0.744838 3.231392 -1.505058

16 H 0.659409 3.811880 0.166298

17 C 0.384456 0.114930 -1.613933

18 H 0.994125 -0.394100 -2.357702

19 H -0.051691 1.006206 -2.060566

20 C -0.725004 -0.842484 -1.180877

21 H -0.317797 -1.732303 -0.706081

22 H -1.316291 -1.123894 -2.054305

23 C -3.263260 -1.210349 0.074870

24 H -3.678093 -1.262016 -0.933073

25 H -2.839529 -2.177995 0.350864

26 C -4.279567 -0.714338 1.097337

27 H -3.824838 -0.637961 2.085459

28 H -5.113605 -1.416422 1.150384

29 H -4.667668 0.264624 0.813286

**Table S20**. Final Cartesian coordinates (X, Y, Z in Å) for the optimized TNZ molecule (conformer no. 2)

**Number Atom X Y Z**

1 S 1.865324 -0.050801 -0.008521

2 O -1.940382 -2.139726 -0.932088

3 O -3.304714 -2.051670 0.765541

4 O 1.224758 -0.001127 1.310099

5 O 2.338352 1.194515 -0.637392

6 N -2.125989 1.900047 1.034944

7 N -1.244289 0.559293 -0.518395

8 N -2.483709 -1.543687 0.010689

9 C -2.670639 0.668504 1.180235

10 H -3.406303 0.432149 1.931067

11 C -2.149087 -0.186565 0.234743

12 C -1.271402 1.818821 0.019621

13 C -0.461396 2.961030 -0.482377

14 H -0.744918 3.231770 -1.504576

15 H 0.607060 2.736227 -0.471187

16 H -0.658701 3.811628 0.166949

17 C -0.384549 0.114922 -1.613947

18 H 0.051536 1.006203 -2.060639

19 H -0.994271 -0.394092 -2.357681

20 C 0.724979 -0.842424 -1.181025

21 H 1.316188 -1.123825 -2.054508

22 H 0.317901 -1.732253 -0.706125

23 C 3.263041 -1.210513 0.075026

24 H 2.838904 -2.178135 0.350488

25 H 3.678389 -1.261975 -0.932711

26 C 4.278896 -0.715097 1.098223

27 H 4.667367 0.263889 0.814746

28 H 5.112767 -1.417368 1.151452

29 H 3.823635 -0.638980 2.086118

**Table S21**. Final Cartesian coordinates (X, Y, Z in Å) for the optimized TNZ molecule (conformer no. 138)

**Number Atom X Y Z**

1 S -2.542312 -0.648863 -0.105303

2 O 2.448027 2.907237 -0.069611

3 O 0.482290 2.082011 0.384216

4 O -2.630297 -1.709619 0.907636

5 O -3.323112 -0.725294 -1.342249

6 N 3.617176 -1.054813 -0.362351

7 N 1.531323 -0.521066 0.228279

8 N 1.687309 1.966792 0.114697

9 C 3.512054 0.296897 -0.354825

10 H 4.329229 0.954168 -0.602168

11 C 2.231360 0.660741 0.003052

12 C 2.423728 -1.526909 -0.012224

13 C 2.093380 -2.972685 0.115817

14 H 1.251421 -3.258574 -0.520217

15 H 1.839250 -3.239097 1.145883

16 H 2.969858 -3.543436 -0.184316

17 C 0.130151 -0.694337 0.614117

18 H -0.100076 0.031797 1.387939

19 H 0.017190 -1.690236 1.035048

20 C -0.794726 -0.525682 -0.587031

21 H -0.666559 -1.323943 -1.319927

22 H -0.656793 0.436792 -1.075196

23 C -2.918428 0.929606 0.712804

24 H -3.917495 0.746841 1.115384

25 H -2.226201 1.020548 1.551119

26 C -2.878673 2.126740 -0.225971

27 H -1.861457 2.345936 -0.551940

28 H -3.248580 3.008657 0.301407

29 H -3.511791 1.955057 -1.096920

**Table S22**. Final Cartesian coordinates (X, Y, Z in Å) for the optimized TNZ molecule (conformer no. 192)

**Number Atom X Y Z**

1 S 2.542503 0.648566 0.105249

2 O -0.482787 -2.082295 -0.383046

3 O -2.449180 -2.906848 0.069072

4 O 3.323291 0.724793 1.342239

5 O 2.630771 1.709321 -0.907688

6 N -3.616791 1.055623 0.362415

7 N -1.531141 0.521158 -0.228267

8 N -1.687984 -1.966658 -0.114527

9 C -3.512201 -0.296129 0.354635

10 H -4.329618 -0.953133 0.601892

11 C -2.231662 -0.660432 -0.003311

12 C -2.423150 1.527300 0.012350

13 C -2.092251 2.972938 -0.115720

14 H -2.968007 3.544106 0.185720

15 H -1.839433 3.239462 -1.146092

16 H -1.249240 3.258259 0.519162

17 C -0.129950 0.693956 -0.614349

18 H -0.016834 1.689588 -1.035897

19 H 0.100042 -0.032666 -1.387771

20 C 0.794917 0.525788 0.586879

21 H 0.656829 -0.436416 1.075549

22 H 0.666753 1.324470 1.319313

23 C 2.918224 -0.930002 -0.712837

24 H 2.226067 -1.020680 -1.551239

25 H 3.917417 -0.747615 -1.115266

26 C 2.877951 -2.127126 0.225910

27 H 3.510820 -1.955549 1.097073

28 H 3.247922 -3.009099 -0.301330

29 H 1.860616 -2.346176 0.551586

**Table S23**. Final Cartesian coordinates (X, Y, Z in Å) for the optimized TNZ molecule (conformer no. 74)

**Number Atom X Y Z**

1 S -2.601298 -0.498191 -0.311575

2 O 0.739753 2.191195 0.012521

3 O 2.822272 2.715893 -0.357272

4 O -2.728498 -1.248033 0.946875

5 O -3.244268 -0.980060 -1.536272

6 N 3.487272 -1.365202 -0.140249

7 N 1.459896 -0.516457 0.254350

8 N 1.935758 1.901277 -0.137724

9 C 3.553887 -0.019831 -0.294190

10 H 4.458111 0.499557 -0.565416

11 C 2.314046 0.535947 -0.060879

12 C 2.228536 -1.644059 0.187951

13 C 1.713146 -3.012979 0.464618

14 H 0.866002 -3.270321 -0.176663

15 H 1.388896 -3.119794 1.503915

16 H 2.521400 -3.717601 0.279411

17 C 0.034507 -0.477551 0.585637

18 H -0.141015 0.352315 1.261415

19 H -0.211619 -1.397921 1.109014

20 C -0.817443 -0.352892 -0.674007

21 H -0.628691 -1.172496 -1.368987

22 H -0.654431 0.596535 -1.181888

23 C -3.182889 1.200333 -0.030049

24 H -2.998397 1.733527 -0.963958

25 H -4.260760 1.058079 0.075863

26 C -2.566992 1.879734 1.184576

27 H -2.674149 1.252847 2.071038

28 H -3.086481 2.822082 1.370930

29 H -1.513453 2.112749 1.027126

**Table S24**. Final Cartesian coordinates (X, Y, Z in Å) for the optimized TNZ molecule (conformer no. 123)

**Number Atom X Y Z**

1 S -2.601361 0.498116 -0.311484

2 O 0.739863 -2.191054 0.012182

3 O 2.822431 -2.715785 -0.357448

4 O -3.244294 0.980087 -1.536190

5 O -2.728497 1.247938 0.946992

6 N 3.487367 1.365286 -0.139888

7 N 1.459898 0.516443 0.254159

8 N 1.935891 -1.901189 -0.137869

9 C 3.554096 0.019875 -0.293635

10 H 4.458428 -0.499546 -0.564438

11 C 2.314222 -0.535912 -0.060611

12 C 2.228516 1.644105 0.187836

13 C 1.712887 3.012994 0.464171

14 H 2.520966 3.717734 0.278664

15 H 1.388780 3.120113 1.503484

16 H 0.865613 3.269965 -0.177090

17 C 0.034472 0.477534 0.585544

18 H -0.211522 1.397977 1.108826

19 H -0.140937 -0.352252 1.261477

20 C -0.817594 0.352687 -0.673992

21 H -0.654557 -0.596866 -1.181634

22 H -0.628966 1.172109 -1.369208

23 C -3.183133 -1.200273 -0.029895

24 H -4.260930 -1.057825 0.076529

25 H -2.999187 -1.733448 -0.963926

26 C -2.566883 -1.879845 1.184458

27 H -1.513268 -2.112418 1.026925

28 H -3.086026 -2.822404 1.370657

29 H -2.674228 -1.253255 2.071127

**Table S25**. Final Cartesian coordinates (X, Y, Z in Å) for the optimized TNZ molecule (conformer no. 4)

**Number Atom X Y Z**

1 S 2.437913 -0.554278 0.296978

2 O -0.562429 2.139340 -0.146678

3 O -2.564900 2.933905 0.183995

4 O 2.609837 -1.444371 -0.860612

5 O 3.166591 -0.806710 1.542457

6 N -3.728213 -1.040871 0.158445

7 N -1.610322 -0.468141 -0.253955

8 N -1.784693 2.007050 0.010552

9 C -3.629655 0.308962 0.245333

10 H -4.464840 0.948065 0.479693

11 C -2.329895 0.695971 -0.001000

12 C -2.511987 -1.488227 -0.140896

13 C -2.166539 -2.921925 -0.343105

14 H -1.367970 -3.249434 0.327864

15 H -1.842670 -3.118366 -1.369312

16 H -3.058273 -3.512129 -0.141797

17 C -0.188158 -0.611959 -0.567101

18 H 0.084314 0.168778 -1.270791

19 H -0.045172 -1.572694 -1.055150

20 C 0.664975 -0.533510 0.696039

21 H 0.522203 -1.398823 1.345058

22 H 0.473278 0.379081 1.258187

23 C 2.780845 1.150182 -0.233935

24 H 2.049124 1.397215 -1.004029

25 H 2.599447 1.776411 0.640365

26 C 4.214396 1.254402 -0.741166

27 H 4.924615 0.987827 0.042669

28 H 4.413371 2.281292 -1.053183

29 H 4.371879 0.594277 -1.594825

**Table S26**. Final Cartesian coordinates (X, Y, Z in Å) for the optimized TNZ molecule (conformer no. 5)

**Number Atom X Y Z**

1 S -2.437926 -0.554351 0.297027

2 O 0.562205 2.139256 -0.147025

3 O 2.564474 2.934009 0.184349

4 O -3.166645 -0.806725 1.542480

5 O -2.609781 -1.444494 -0.860527

6 N 3.728425 -1.040577 0.158167

7 N 1.610347 -0.468168 -0.253770

8 N 1.784433 2.007064 0.010587

9 C 3.629648 0.309227 0.245172

10 H 4.464748 0.948471 0.479443

11 C 2.329784 0.696059 -0.000916

12 C 2.512195 -1.488102 -0.140974

13 C 2.166954 -2.921864 -0.343078

14 H 1.842660 -3.118369 -1.369135

15 H 1.368766 -3.249547 0.328262

16 H 3.058903 -3.511892 -0.142201

17 C 0.188177 -0.612151 -0.566934

18 H 0.045302 -1.572971 -1.054829

19 H -0.084345 0.168463 -1.270736

20 C -0.664987 -0.533586 0.696165

21 H -0.473304 0.379046 1.258251

22 H -0.522264 -1.398839 1.345275

23 C -2.780827 1.150136 -0.233983

24 H -2.599583 1.776368 0.640344

25 H -2.048976 1.397163 -1.003952

26 C -4.214281 1.254293 -0.741440

27 H -4.371572 0.594310 -1.595245

28 H -4.413324 2.281223 -1.053300

29 H -4.924632 0.987512 0.042212

**Table S27**. Final Cartesian coordinates (X, Y, Z in Å) for the optimized TNZ molecule (conformer no. 143)

**Number Atom X Y Z**

1 S 1.743393 0.435603 -0.718546

2 O -0.100566 -2.084801 1.067834

3 O -1.415107 -3.030253 -0.384691

4 O 2.687095 1.349436 -1.370353

5 O 0.703835 -0.245833 -1.496214

6 N -3.227458 0.669962 -0.636480

7 N -1.369653 0.399423 0.575231

8 N -1.030494 -2.057522 0.246257

9 C -2.859884 -0.635758 -0.702263

10 H -3.412343 -1.377350 -1.254408

11 C -1.717472 -0.835089 0.036958

12 C -2.321789 1.273523 0.121490

13 C -2.343578 2.723846 0.456296

14 H -1.522939 3.267456 -0.020620

15 H -2.280922 2.896584 1.534458

16 H -3.281925 3.135168 0.089471

17 C -0.197488 0.771667 1.366669

18 H 0.154674 -0.119806 1.873466

19 H -0.530689 1.481794 2.126223

20 C 0.916322 1.440216 0.561108

21 H 1.701113 1.800973 1.227220

22 H 0.553722 2.291633 -0.016233

23 C 2.695627 -0.830917 0.162494

24 H 1.970050 -1.448315 0.691335

25 H 3.099575 -1.420788 -0.663844

26 C 3.796215 -0.257325 1.042887

27 H 4.445541 0.406554 0.471205

28 H 4.401649 -1.072771 1.443842

29 H 3.391670 0.296806 1.893114

**Table S28**. Final Cartesian coordinates (X, Y, Z in Å) for the optimized TNZ molecule (conformer no. 54)

**Number Atom X Y Z**

1 S 1.743432 0.435443 0.718480

2 O -0.100607 -2.085055 -1.066893

3 O -1.416676 -3.030227 0.384393

4 O 0.703666 -0.245619 1.496173

5 O 2.687117 1.349368 1.370186

6 N -3.227239 0.670785 0.636375

7 N -1.369421 0.399574 -0.575161

8 N -1.031195 -2.057531 -0.246097

9 C -2.860304 -0.635111 0.701952

10 H -3.413131 -1.376528 1.253968

11 C -1.717903 -0.834884 -0.037177

12 C -2.321220 1.274035 -0.121445

13 C -2.342415 2.724382 -0.456174

14 H -3.280405 3.136133 -0.088932

15 H -2.280142 2.897105 -1.534364

16 H -1.521316 3.267594 0.020391

17 C -0.197297 0.771300 -1.366916

18 H -0.530439 1.481270 -2.126650

19 H 0.154609 -0.120415 -1.873471

20 C 0.916795 1.439792 -0.561707

21 H 0.554601 2.291656 0.015218

22 H 1.701726 1.799886 -1.228021

23 C 2.695762 -0.831406 -0.161967

24 H 3.099850 -1.420831 0.664619

25 H 1.970217 -1.449085 -0.690512

26 C 3.796210 -0.258050 -1.042701

27 H 3.391493 0.295398 -1.893288

28 H 4.401938 -1.073549 -1.443101

29 H 4.445313 0.406396 -0.471423

**Table S29**. Final Cartesian coordinates (X, Y, Z in Å) for the optimized TNZ molecule (conformer no. 50)

**Number Atom X Y Z**

1 S -2.463354 0.375360 -0.180097

2 O 1.353820 -2.295636 -0.576492

3 O 3.416712 -2.473691 0.106318

4 O -2.859021 -0.100246 -1.510302

5 O -2.392444 1.817351 0.096879

6 N 3.161954 1.627932 0.655669

7 N 1.481910 0.488739 -0.272200

8 N 2.416502 -1.818175 -0.159360

9 C 3.512995 0.318303 0.604354

10 H 4.455752 -0.054986 0.968807

11 C 2.491498 -0.414685 0.040292

12 C 1.943717 1.712136 0.128116

13 C 1.167979 2.973858 -0.024530

14 H 0.147320 2.882831 0.355799

15 H 1.101138 3.273760 -1.075281

16 H 1.691230 3.756588 0.521270

17 C 0.168735 0.240868 -0.861868

18 H 0.265859 -0.460118 -1.683816

19 H -0.176785 1.194604 -1.256573

20 C -0.816794 -0.296452 0.185675

21 H -0.557951 0.062608 1.182088

22 H -0.849815 -1.383574 0.173700

23 C -3.553532 -0.378056 1.060455

24 H -3.117684 -0.136465 2.031698

25 H -4.475524 0.197116 0.949664

26 C -3.771696 -1.868427 0.840873

27 H -4.157358 -2.055254 -0.161422

28 H -4.495975 -2.241975 1.567668

29 H -2.849036 -2.437867 0.967763

**Table S30**. Final Cartesian coordinates (X, Y, Z in Å) for the optimized TNZ molecule (conformer no. 79)

**Number Atom X Y Z**

1 S 2.463312 0.375526 -0.179851

2 O -1.353762 -2.295692 -0.576218

3 O -3.416655 -2.473815 0.106593

4 O 2.392273 1.817378 0.097877

5 O 2.858862 -0.099329 -1.510339

6 N -3.162209 1.627972 0.655151

7 N -1.481888 0.488623 -0.272007

8 N -2.416504 -1.818245 -0.159185

9 C -3.513265 0.318366 0.603890

10 H -4.456118 -0.054869 0.968151

11 C -2.491605 -0.414750 0.040251

12 C -1.943786 1.712069 0.127957

13 C -1.167955 2.973744 -0.024544

14 H -1.691484 3.756572 0.520846

15 H -1.100580 3.273473 -1.075317

16 H -0.147474 2.882742 0.356277

17 C -0.168723 0.240778 -0.861736

18 H 0.176740 1.194550 -1.256406

19 H -0.265875 -0.460149 -1.683725

20 C 0.816821 -0.296591 0.185755

21 H 0.849969 -1.383706 0.173662

22 H 0.557944 0.062299 1.182216

23 C 3.553743 -0.378393 1.060189

24 H 4.475618 0.197003 0.949579

25 H 3.117982 -0.137379 2.031613

26 C 3.772203 -1.868592 0.839812

27 H 2.849648 -2.438306 0.966266

28 H 4.496444 -2.242395 1.566520

29 H 4.158042 -2.054805 -0.162528

**Table S31**. Final Cartesian coordinates (X, Y, Z in Å) for the optimized TNZ molecule (conformer no. 35)

**Number Atom X Y Z**

1 S 1.695506 -0.842690 -0.390976

2 O 0.156487 1.934540 1.242635

3 O -0.752686 3.125901 -0.333450

4 O 0.902961 0.048248 -1.245970

5 O 2.524119 -1.894966 -0.987298

6 N -3.194543 -0.147998 -0.928263

7 N -1.489911 -0.254588 0.512439

8 N -0.647951 2.093542 0.311531

9 C -2.580644 1.063335 -0.900767

10 H -2.906375 1.903505 -1.490709

11 C -1.525839 1.030346 -0.019286

12 C -2.524436 -0.923494 -0.086740

13 C -2.863345 -2.346056 0.191167

14 H -3.795082 -2.572000 -0.323624

15 H -2.998361 -2.533372 1.260400

16 H -2.094250 -3.031192 -0.176614

17 C -0.520212 -0.855087 1.427510

18 H -1.074090 -1.506281 2.106673

19 H -0.080974 -0.055654 2.013543

20 C 0.550078 -1.703147 0.739366

21 H 0.117913 -2.470664 0.095761

22 H 1.173803 -2.202054 1.483370

23 C 2.745330 0.177026 0.683008

24 H 2.074523 0.830696 1.239218

25 H 3.257184 -0.516900 1.352201

26 C 3.718358 0.979764 -0.174837

27 H 4.350390 0.321994 -0.772685

28 H 4.356156 1.581513 0.475102

29 H 3.175109 1.650843 -0.840822

**Table S32**. Final Cartesian coordinates (X, Y, Z in Å) for the optimized TNZ molecule (conformer no. 93)

**Number Atom X Y Z**

1 S -1.695501 -0.842671 -0.390862

2 O -0.156485 1.934625 1.242464

3 O 0.753255 3.126046 -0.333255

4 O -2.524096 -1.895041 -0.987057

5 O -0.902826 0.048040 -1.245945

6 N 3.194342 -0.148327 -0.928411

7 N 1.489838 -0.254630 0.512457

8 N 0.648197 2.093616 0.311563

9 C 2.580692 1.063108 -0.900821

10 H 2.906524 1.903226 -1.490786

11 C 1.525972 1.030322 -0.019211

12 C 2.524153 -0.923720 -0.086830

13 C 2.862851 -2.346347 0.190995

14 H 2.093488 -3.031343 -0.176492

15 H 2.998210 -2.533664 1.260184

16 H 3.794347 -2.572459 -0.324154

17 C 0.520178 -0.854935 1.427710

18 H 0.081053 -0.055380 2.013660

19 H 1.074069 -1.506096 2.106893

20 C -0.550263 -1.702975 0.739778

21 H -1.174077 -2.201558 1.483929

22 H -0.118270 -2.470788 0.096410

23 C -2.745404 0.177307 0.682777

24 H -3.257466 -0.516478 1.351956

25 H -2.074615 0.830942 1.239053

26 C -3.718156 0.980081 -0.175344

27 H -3.174674 1.650877 -0.841421

28 H -4.355866 1.582129 0.474402

29 H -4.350278 0.322323 -0.773108

**Table S33**. Final Cartesian coordinates (X, Y, Z in Å) for the optimized TNZ molecule (conformer no. 57)

**Number Atom X Y Z**

1 S -1.987560 -0.216172 0.704765

2 O 1.876894 -2.228116 -0.748716

3 O 3.689104 -1.849366 0.402029

4 O -1.296499 0.922183 1.322921

5 O -2.614606 -1.250864 1.529091

6 N 2.394543 2.075361 0.444636

7 N 1.160233 0.487600 -0.525474

8 N 2.642490 -1.484407 -0.118791

9 C 3.033342 0.890257 0.599471

10 H 3.978800 0.789972 1.106317

11 C 2.299851 -0.113921 0.008096

12 C 1.276650 1.817961 -0.226341

13 C 0.281617 2.856201 -0.605560

14 H -0.647582 2.710008 -0.051894

15 H 0.067482 2.849248 -1.678363

16 H 0.701650 3.825195 -0.342968

17 C 0.063273 -0.146387 -1.252168

18 H 0.488066 -0.752103 -2.050124

19 H -0.517726 0.653212 -1.709128

20 C -0.818273 -1.058437 -0.398825

21 H -0.224785 -1.689592 0.259996

22 H -1.411555 -1.717877 -1.033313

23 C -3.263926 0.473626 -0.394513

24 H -3.833623 1.112837 0.283503

25 H -2.750749 1.120457 -1.108889

26 C -4.135773 -0.585655 -1.052409

27 H -3.571916 -1.198995 -1.758705

28 H -4.945350 -0.104707 -1.605426

29 H -4.572314 -1.241347 -0.298940

**Table S34**. Final Cartesian coordinates (X, Y, Z in Å) for the optimized TNZ molecule (conformer no. 111)

**Number Atom X Y Z**

1 S 1.987698 -0.215985 0.704900

2 8 -1.876689 -2.228157 -0.748809

3 8 -3.688804 -1.849661 0.402183

4 8 2.614645 -1.250629 1.529387

5 8 1.296712 0.922505 1.322883

6 7 -2.394926 2.075281 0.444363

7 7 -1.160221 0.487590 -0.525375

8 7 -2.642290 -1.484576 -0.118747

9 6 -3.033571 0.890094 0.599225

10 1 -3.979094 0.789713 1.105933

11 6 -2.299829 -0.114037 0.008085

12 6 -1.276892 1.817963 -0.226419

13 6 -0.281884 2.856263 -0.605528

14 1 -0.067669 2.849360 -1.678317

15 1 0.647277 2.710064 -0.051801

16 1 -0.701950 3.825243 -0.342940

17 6 -0.063139 -0.146319 -1.251982

18 1 0.517886 0.653335 -1.708804

19 1 -0.487818 -0.751986 -2.050036

20 6 0.818346 -1.058341 -0.398548

21 1 1.411573 -1.717929 -1.032923

22 1 0.224867 -1.689376 0.260396

23 6 3.264156 0.473543 -0.394390

24 1 2.751223 1.121196 -1.108194

25 1 3.834516 1.111917 0.283862

26 6 4.135152 -0.585901 -1.053175

27 1 4.571469 -1.242329 -0.300212

28 1 4.944924 -0.105145 -1.606070

29 1 3.570744 -1.198425 -1.759734

**Table S35**. Final Cartesian coordinates (X, Y, Z in Å) for the optimized TNZ molecule (conformer no. 37)

**Number Atom X Y Z**

1 S 1.940250 -0.645223 -0.412625

2 O -2.264669 -2.019068 0.780748

3 O -3.902800 -1.453733 -0.541665

4 O 1.452197 0.521870 -1.159528

5 O 2.488660 -1.803534 -1.120010

6 N -2.050693 2.240135 -0.642330

7 N -1.148582 0.552880 0.508668

8 N -2.860911 -1.211063 0.054424

9 C -2.840822 1.150383 -0.800791

10 H -3.744042 1.155321 -1.388120

11 C -2.314728 0.088798 -0.099168

12 C -1.043467 1.866372 0.139540

13 C 0.058262 2.772679 0.559880

14 H 1.000373 2.457193 0.107891

15 H 0.172366 2.806438 1.647383

16 H -0.185961 3.772614 0.206691

17 C -0.222849 -0.183301 1.365928

18 H -0.799780 -0.681083 2.142837

19 H 0.420042 0.553667 1.844536

20 C 0.596924 -1.253465 0.645368

21 H -0.024741 -1.856903 -0.013877

22 H 1.060514 -1.927942 1.367410

23 C 3.202921 -0.057412 0.760793

24 H 2.730381 0.703887 1.384579

25 H 3.475055 -0.917287 1.375212

26 C 4.396677 0.499848 -0.007187

27 H 4.840035 -0.270105 -0.639515

28 H 5.153643 0.849632 0.697138

29 H 4.094806 1.336783 -0.638043

**Table S36**. Final Cartesian coordinates (X, Y, Z in Å) for the optimized TNZ molecule (conformer no. 68)

**Number Atom X Y Z**

1 S -1.939876 -0.646093 -0.412606

2 O 2.265101 -2.018563 0.781160

3 O 3.902608 -1.453020 -0.541943

4 O -2.487592 -1.804603 -1.120169

5 O -1.452007 0.521251 -1.159245

6 N 2.049515 2.240319 -0.642798

7 N 1.148365 0.553141 0.509088

8 N 2.860857 -1.210517 0.054460

9 C 2.839737 1.150689 -0.801497

10 H 3.742652 1.155728 -1.389279

11 C 2.314192 0.089101 -0.099433

12 C 1.042758 1.866523 0.139659

13 C -0.058937 2.772732 0.560315

14 H -0.172616 2.806718 1.647850

15 H -1.001174 2.457005 0.108737

16 H 0.185011 3.772614 0.206777

17 C 0.222743 -0.183189 1.366335

18 H -0.420411 0.553704 1.844700

19 H 0.799678 -0.680589 2.143469

20 C -0.596595 -1.253722 0.645851

21 H -1.060165 -1.928154 1.367953

22 H 0.025377 -1.857145 -0.013097

23 C -3.203142 -0.058819 0.760508

24 H -3.477220 -0.919656 1.372696

25 H -2.730323 0.700401 1.386615

26 C -4.395117 0.501847 -0.007749

27 H -4.091325 1.339748 -0.636393

28 H -5.152506 0.851072 0.696410

29 H -4.838825 -0.266047 -0.642362

**Table S37**. Final Cartesian coordinates (X, Y, Z in Å) for the optimized TNZ molecule (conformer no. 65)

**Number Atom X Y Z**

1 S 1.797765 0.897106 -0.036879

2 O -2.113949 1.946455 -0.981373

3 O -2.580547 1.940073 1.146101

4 O 0.848885 2.012202 0.039937

5 O 3.237872 1.131993 -0.176856

6 N -1.678237 -2.101127 0.867969

7 N -1.000019 -0.655813 -0.693628

8 N -2.159975 1.414899 0.124865

9 C -2.153862 -0.863883 1.188585

10 H -2.780253 -0.676863 2.044801

11 C -1.748913 0.046144 0.242752

12 C -0.987921 -1.956419 -0.253940

13 C -0.298774 -3.068564 -0.965265

14 H 0.781219 -3.066259 -0.792139

15 H -0.466459 -3.027200 -2.044174

16 H -0.696194 -4.006526 -0.581445

17 C -0.180021 -0.108437 -1.778205

18 H -0.518353 0.907763 -1.952695

19 H -0.370506 -0.692605 -2.680992

20 C 1.314052 -0.125461 -1.474601

21 H 1.863847 0.338882 -2.295286

22 H 1.724781 -1.121918 -1.318179

23 C 1.551213 -0.133623 1.438771

24 H 1.722731 0.584831 2.243818

25 H 0.499891 -0.415319 1.463146

26 C 2.502259 -1.317370 1.522588

27 H 2.322538 -2.044123 0.727476

28 H 2.354752 -1.833298 2.473430

29 H 3.539095 -0.986873 1.461273

**Table S38**. Final Cartesian coordinates (X, Y, Z in Å) for the optimized TNZ molecule (conformer no. 55)

**Number Atom X Y Z**

1 S 1.797621 -0.897355 -0.036858

2 O -2.114498 -1.946201 -0.981262

3 O -2.580981 -1.939563 1.146227

4 O 3.237659 -1.132577 -0.176884

5 O 0.848494 -2.012222 0.040134

6 N -1.677887 2.101427 0.867877

7 N -1.000030 0.655921 -0.693706

8 N -2.160359 -1.414538 0.124945

9 C -2.153844 0.864321 1.188504

10 H -2.780338 0.677499 2.044690

11 C -1.749061 -0.045842 0.242731

12 C -0.987563 1.956520 -0.254004

13 C -0.297979 3.068453 -0.965219

14 H -0.694649 4.006504 -0.580850

15 H -0.466114 3.027602 -2.044087

16 H 0.782083 3.065386 -0.792531

17 C -0.180072 0.108326 -1.778212

18 H -0.370433 0.692414 -2.681076

19 H -0.518545 -0.907842 -1.952566

20 C 1.314024 0.125162 -1.474616

21 H 1.724874 1.121581 -1.318279

22 H 1.863698 -0.339270 -2.295329

23 C 1.551374 0.133590 1.438662

24 H 0.500181 0.415761 1.462931

25 H 1.722507 -0.584847 2.243806

26 C 2.502904 1.316961 1.522451

27 H 3.539611 0.986006 1.461519

28 H 2.355301 1.833191 2.473114

29 H 2.323737 2.043580 0.727089

**Table S39**. Final Cartesian coordinates (X, Y, Z in Å) for the optimized TNZ molecule (conformer no. 36)

**Number Atom X Y Z**

1 S 1.978971 0.070817 -0.246468

2 O -1.255562 -2.010046 -1.685173

3 O -1.388669 -2.840152 0.324757

4 O 3.400915 0.400627 -0.375811

5 O 1.484839 -1.265929 -0.595664

6 N -2.284957 1.085122 1.359994

7 N -1.223331 0.596014 -0.543084

8 N -1.361508 -1.907442 -0.466795

9 C -2.186196 -0.271888 1.255906

10 H -2.604975 -0.950214 1.980447

11 C -1.533081 -0.598938 0.093662

12 C -1.697732 1.585697 0.282756

13 C -1.563185 3.041032 -0.002901

14 H -2.254031 3.574449 0.647603

15 H -1.801675 3.276630 -1.042861

16 H -0.553949 3.409738 0.203498

17 C -0.329517 0.791955 -1.687837

18 H -0.783294 1.528228 -2.354066

19 H -0.271120 -0.155414 -2.214663

20 C 1.066079 1.264987 -1.289886

21 H 1.078377 2.222646 -0.769037

22 H 1.696025 1.352671 -2.176920

23 C 1.468562 0.441371 1.464309

24 H 2.021578 1.339474 1.742543

25 H 0.401682 0.657667 1.452103

26 C 1.777823 -0.748129 2.365726

27 H 1.224392 -1.628305 2.038177

28 H 1.479897 -0.507526 3.388057

29 H 2.843438 -0.981487 2.362535

**Table S40**. Final Cartesian coordinates (X, Y, Z in Å) for the optimized TNZ molecule (conformer no. 125)

**Number Atom X Y Z**

1 S 1.978993 -0.070733 -0.246392

2 O -1.255498 2.010051 -1.685192

3 O -1.388689 2.840134 0.324745

4 O 1.484877 1.266061 -0.595402

5 O 3.400936 -0.400521 -0.375699

6 N -2.284834 -1.085180 1.359936

7 N -1.223277 -0.596026 -0.543160

8 N -1.361455 1.907411 -0.466811

9 C -2.186057 0.271822 1.255905

10 H -2.604859 0.950128 1.980453

11 C -1.533024 0.598913 0.093615

12 C -1.697627 -1.585733 0.282680

13 C -1.563160 -3.041053 -0.003075

14 H -0.553691 -3.409642 0.202381

15 H -1.802584 -3.276678 -1.042814

16 H -2.253360 -3.574524 0.648069

17 C -0.329476 -0.791926 -1.687922

18 H -0.271248 0.155403 -2.214836

19 H -0.783160 -1.528347 -2.354053

20 C 1.066203 -1.264764 -1.290053

21 H 1.696132 -1.352102 -2.177136

22 H 1.078716 -2.222554 -0.769449

23 C 1.468511 -0.441574 1.464324

24 H 0.401720 -0.658315 1.451974

25 H 2.021844 -1.339483 1.742536

26 C 1.777178 0.747951 2.365916

27 H 2.842766 0.981408 2.363295

28 H 1.478679 0.507315 3.388073

29 H 1.223828 1.628072 2.038074

**Table S41**. Final Cartesian coordinates (X, Y, Z in Å) for the optimized TNZ molecule (conformer no. 173)

**Number Atom X Y Z**

1 S 1.977390 0.049778 -0.254610

2 O -1.431910 -2.827788 0.345206

3 O -1.286116 -2.010797 -1.669076

4 O 3.401796 0.364328 -0.394619

5 O 1.468683 -1.286120 -0.585632

6 N -2.268370 1.114598 1.362039

7 N -1.220464 0.601395 -0.542299

8 N -1.390878 -1.899878 -0.451228

9 C -2.187365 -0.244094 1.265219

10 H -2.611838 -0.912921 1.995246

11 C -1.543042 -0.585997 0.102296

12 C -1.679077 1.601574 0.279746

13 C -1.527455 3.053489 -0.014475

14 H -2.214309 3.598605 0.630584

15 H -1.759889 3.285414 -1.056584

16 H -0.514866 3.412277 0.193278

17 C -0.330670 0.779210 -1.693045

18 H -0.780545 1.514194 -2.363371

19 H -0.284173 -0.173082 -2.212012

20 C 1.071539 1.241919 -1.306285

21 H 1.096003 2.204613 -0.795254

22 H 1.698147 1.314288 -2.197037

23 C 1.480142 0.443890 1.454231

24 H 2.026466 1.351839 1.713260

25 H 0.410954 0.648748 1.448841

26 C 1.811053 -0.727845 2.371275

27 H 1.263569 -1.618743 2.063222

28 H 1.520888 -0.474327 3.392689

29 H 2.878973 -0.950021 2.360525

**Table S42**. Final Cartesian coordinates (X, Y, Z in Å) for the optimized TNZ molecule (conformer no. 17)

**Number Atom X Y Z**

1 S 2.500665 -0.774751 0.064257

2 O -1.666379 -2.261107 0.153342

3 O -3.761157 -2.006093 -0.394880

4 O 3.169938 -1.385402 -1.085559

5 O 2.467607 -1.438297 1.371249

6 N -2.898060 2.041840 -0.257231

7 N -1.362961 0.525417 0.319641

8 N -2.656285 -1.568111 -0.101465

9 C -3.441689 0.806009 -0.407218

10 H -4.448742 0.641768 -0.752800

11 C -2.515421 -0.152283 -0.062836

12 C -1.657447 1.853674 0.176397

13 C -0.698134 2.951404 0.482248

14 H 0.224124 2.864998 -0.099367

15 H -0.428201 2.972189 1.542436

16 H -1.180514 3.894026 0.231368

17 C -0.074468 -0.010266 0.763409

18 H -0.239230 -0.850968 1.429038

19 H 0.410285 0.782402 1.331041

20 C 0.780950 -0.450021 -0.423047

21 H 0.801299 0.290346 -1.223588

22 H 0.426470 -1.399111 -0.824712

23 C 3.230144 0.873737 0.318091

24 H 2.624182 1.375492 1.074608

25 H 4.194436 0.633979 0.771324

26 C 3.385291 1.674007 -0.966715

27 H 3.947516 1.103368 -1.705963

28 H 3.923611 2.601628 -0.761016

29 H 2.419837 1.941500 -1.402301

**Table S43**. Final Cartesian coordinates (X, Y, Z in Å) for the optimized TNZ molecule (conformer no. 24)

**Number Atom X Y Z**

1 S 2.500668 -0.774876 -0.063927

2 O -1.665919 -2.261052 -0.152628

3 O -3.761194 -2.006306 0.393774

4 O 2.467367 -1.439475 -1.370384

5 O 3.170070 -1.384663 1.086295

6 N -2.898274 2.041724 0.257229

7 N -1.362917 0.525466 -0.319413

8 N -2.656150 -1.568191 0.101246

9 C -3.441897 0.805823 0.406812

10 H -4.449046 0.641477 0.752061

11 C -2.515471 -0.152341 0.062539

12 C -1.657539 1.853700 -0.176046

13 C -0.698168 2.951493 -0.481471

14 H -0.428005 2.972518 -1.541592

15 H 0.223965 2.864964 0.100328

16 H -1.180606 3.894055 -0.230483

17 C -0.074338 -0.010029 -0.763115

18 H 0.410345 0.782729 -1.330703

19 H -0.238902 -0.850758 -1.428762

20 C 0.781078 -0.449647 0.423385

21 H 0.426424 -1.398579 0.825269

22 H 0.801575 0.290932 1.223720

23 C 3.230300 0.873301 -0.319137

24 H 4.194777 0.633061 -0.771723

25 H 2.624682 1.374336 -1.076402

26 C 3.384938 1.674793 0.964981

27 H 2.419291 1.942589 1.399951

28 H 3.923264 2.602261 0.758644

29 H 3.946914 1.104854 1.704957

**Table S44**. Final Cartesian coordinates (X, Y, Z in Å) for the optimized TNZ molecule (conformer no. 58)

**Number Atom X Y Z**

1 S 1.813900 -0.663140 -0.639412

2 O -0.193809 2.246615 0.972547

3 O -1.335271 2.977075 -0.728023

4 O 0.941333 0.349095 -1.241229

5 O 2.338934 -1.771134 -1.442003

6 N -3.137241 -0.737050 -0.663583

7 N -1.322061 -0.336001 0.575334

8 N -1.012322 2.097388 0.056669

9 C -2.774796 0.561726 -0.839701

10 H -3.317408 1.244825 -1.471291

11 C -1.660211 0.839539 -0.086112

12 C -2.254145 -1.259076 0.175215

13 C -2.276887 -2.675026 0.635500

14 H -3.251128 -3.090354 0.384242

15 H -2.122878 -2.761574 1.714351

16 H -1.514723 -3.279584 0.134699

17 C -0.166500 -0.609805 1.428535

18 H -0.521178 -1.161226 2.302538

19 H 0.213359 0.346739 1.767530

20 C 0.917375 -1.448617 0.751944

21 H 0.516319 -2.351937 0.293185

22 H 1.674159 -1.752054 1.478222

23 C 3.244090 0.177635 0.103349

24 H 3.859950 -0.614681 0.532697

25 H 3.757902 0.566757 -0.778652

26 C 2.892544 1.279561 1.091588

27 H 2.179603 1.985790 0.667061

28 H 3.800750 1.822082 1.362522

29 H 2.465929 0.876264 2.012315

**Table S45**. Final Cartesian coordinates (X, Y, Z in Å) for the optimized TNZ molecule (conformer no. 144)

**Number Atom X Y Z**

1 S -1.813505 -0.663295 -0.639235

2 O 0.193618 2.246764 0.972214

3 O 1.335803 2.977015 -0.728024

4 O -2.338226 -1.771872 -1.441183

5 O -0.940568 0.348330 -1.241423

6 N 3.136820 -0.737502 -0.663557

7 N 1.321779 -0.335993 0.575374

8 N 1.012544 2.097418 0.056657

9 C 2.774989 0.561439 -0.839361

10 H 3.317856 1.244498 -1.470777

11 C 1.660549 0.839632 -0.085644

12 C 2.253443 -1.259346 0.175132

13 C 2.275600 -2.675473 0.634885

14 H 1.513238 -3.279505 0.133746

15 H 2.121494 -2.762461 1.713700

16 H 3.249729 -3.091014 0.383543

17 C 0.166562 -0.609348 1.429240

18 H -0.212762 0.347360 1.768389

19 H 0.521555 -1.160905 2.303029

20 C -0.917930 -1.447852 0.753359

21 H -1.675161 -1.749559 1.479892

22 H -0.517674 -2.352195 0.295906

23 C -3.244010 0.178206 0.102266

24 H -3.757355 0.566861 -0.780210

25 H -3.860159 -0.613805 0.531757

26 C -2.892690 1.280531 1.090062

27 H -2.466039 0.877620 2.010955

28 H -3.800986 1.823027 1.360791

29 H -2.179754 1.986703 0.665427

**Table S46**. Final Cartesian coordinates (X, Y, Z in Å) for the optimized TNZ molecule (conformer no. 48)

**Number Atom X Y Z**

1 S -1.865207 -0.050833 0.008393

2 O 1.940218 -2.139705 0.932222

3 O 3.304459 -2.051830 -0.765500

4 O -1.224595 -0.001689 -1.310249

5 O -2.338059 1.194724 0.636862

6 N 2.125962 1.899919 -1.035063

7 N 1.244326 0.559328 0.518457

8 N 2.483517 -1.543772 -0.010638

9 C 2.670470 0.668300 -1.180356

10 H 3.406021 0.431820 -1.931261

11 C 2.148955 -0.186648 -0.234748

12 C 1.271512 1.818837 -0.019617

13 C 0.461717 2.961175 0.482431

14 H 0.659649 3.811951 -0.166459

15 H 0.744804 3.231397 1.504892

16 H -0.606817 2.736733 0.470689

17 C 0.384522 0.115127 1.614014

18 H -0.051637 1.006466 2.060507

19 H 0.994161 -0.393793 2.357873

20 C -0.725001 -0.842300 1.181123

21 H -1.316287 -1.123570 2.054590

22 H -0.317900 -1.732195 0.706367

23 C -3.263095 -1.210360 -0.074813

24 H -2.839182 -2.178087 -0.350240

25 H -3.678303 -1.261663 0.932998

26 C -4.279039 -0.714859 -1.097874

27 H -4.667350 0.264186 -0.814382

28 H -5.113001 -1.417030 -1.150961

29 H -3.823926 -0.638834 -2.085845

**Table S47**. Final Cartesian coordinates (X, Y, Z in Å) for the optimized TNZ molecule (conformer no. 73)

**Number Atom X Y Z**

1 S -2.421895 -0.645356 -0.138116

2 O 1.635559 -2.223593 0.327237

3 O 3.777602 -2.108326 -0.062368

4 O -2.476227 -1.128809 1.246448

5 O -3.018661 -1.415747 -1.230310

6 N 3.108527 1.968756 -0.345867

7 N 1.462016 0.578153 0.240592

8 N 2.676262 -1.598868 0.099521

9 C 3.599250 0.702173 -0.347075

10 H 4.618935 0.464063 -0.600332

11 C 2.604059 -0.180358 0.006990

12 C 1.832045 1.874260 0.007000

13 C 0.909667 3.035536 0.145492

14 H 1.454262 3.929935 -0.150576

15 H 0.569089 3.163425 1.177452

16 H 0.026565 2.936566 -0.492138

17 C 0.123726 0.139365 0.641380

18 H -0.358453 0.996425 1.108660

19 H 0.206737 -0.647314 1.383890

20 C -0.675261 -0.367432 -0.557126

21 H -0.310829 -1.343082 -0.878407

22 H -0.652132 0.321976 -1.402846

23 C -3.152280 1.023885 -0.173092

24 H -2.582698 1.635934 0.529047

25 H -3.009590 1.397715 -1.188475

26 C -4.625927 0.946867 0.210162

27 H -5.173220 0.314927 -0.490080

28 H -5.062363 1.947243 0.189100

29 H -4.741484 0.537607 1.214386

**Table S48**. Final Cartesian coordinates (X, Y, Z in Å) for the optimized TNZ molecule (conformer no. 85)

**Number Atom X Y Z**

1 S 2.511676 -0.768552 -0.112894

2 O -3.604857 -2.190807 -0.008503

3 O -1.456693 -2.237957 0.360867

4 O 2.953097 -1.823407 -1.025359

5 O 2.549522 -0.954934 1.342978

6 N -3.058492 1.894446 -0.389487

7 N -1.363394 0.567020 0.206910

8 N -2.516374 -1.647544 0.129570

9 C -3.510248 0.614040 -0.359235

10 H -4.524854 0.339881 -0.595797

11 C -2.485018 -0.230076 0.002444

12 C -1.776027 1.846117 -0.048421

13 C -0.891628 3.039877 0.054399

14 H -0.042569 2.979848 -0.632632

15 H -0.499153 3.172416 1.066722

16 H -1.484380 3.915012 -0.204444

17 C -0.011677 0.174818 0.615085

18 H -0.074215 -0.593992 1.377955

19 H 0.453240 1.052140 1.058147

20 C 0.792695 -0.347158 -0.573516

21 H 0.836364 0.363605 -1.400717

22 H 0.377134 -1.289206 -0.929395

23 C 3.491347 0.713455 -0.508423

24 H 4.514120 0.362437 -0.355763

25 H 3.350776 0.893628 -1.575496

26 C 3.167659 1.918117 0.362354

27 H 2.169335 2.310241 0.159385

28 H 3.881344 2.720035 0.162657

29 H 3.232691 1.655024 1.418736

**Table S59**. Final Cartesian coordinates (X, Y, Z in Å) for the optimized TNZ molecule (conformer no. 88)

**Number Atom X Y Z**

1 S -2.511757 -0.768574 -0.112906

2 O 1.456919 -2.238168 0.359646

3 O 3.605402 -2.190425 -0.007676

4 O -2.549388 -0.955415 1.342930

5 O -2.953418 -1.823142 -1.025605

6 N 3.058085 1.894656 -0.389551

7 N 1.363243 0.566910 0.206789

8 N 2.516689 -1.647441 0.129559

9 C 3.510162 0.614371 -0.359048

10 H 4.524846 0.340461 -0.595564

11 C 2.485081 -0.229950 0.002597

12 C 1.775615 1.846078 -0.048572

13 C 0.890948 3.039642 0.054213

14 H 0.497385 3.171340 1.066220

15 H 0.042611 2.980029 -0.633750

16 H 1.483852 3.915046 -0.203378

17 C 0.011665 0.174392 0.615018

18 H -0.453296 1.051387 1.058715

19 H 0.074424 -0.594865 1.377411

20 C -0.792911 -0.347037 -0.573662

21 H -0.377420 -1.288920 -0.930088

22 H -0.836645 0.364056 -1.400586

23 C -3.491513 0.713488 -0.507605

24 H -3.352183 0.893391 -1.574884

25 H -4.514205 0.362788 -0.353616

26 C -3.166478 1.918228 0.362570

27 H -3.230262 1.655205 1.419057

28 H -3.880308 2.720222 0.163736

29 H -2.168353 2.310102 0.158214

**Table S50**. Final Cartesian coordinates (X, Y, Z in Å) for the optimized TNZ molecule (conformer no. 200)

**Number Atom X Y Z**

1 S -1.631513 0.129219 -0.482636

2 O 2.399295 -2.578054 -0.707392

3 O 1.354783 -2.299580 1.180349

4 O -1.143287 -1.250611 -0.573518

5 O -1.184143 1.151604 -1.434630

6 N 2.519608 1.547673 -0.836614

7 N 1.201051 0.500603 0.630506

8 N 1.867725 -1.874319 0.141881

9 C 2.696889 0.207016 -0.980675

10 H 3.389650 -0.223987 -1.683846

11 C 1.898143 -0.467822 -0.089747

12 C 1.613668 1.703169 0.115911

13 C 1.067104 3.018883 0.541709

14 H 1.781135 3.790679 0.259206

15 H 0.894771 3.070325 1.619641

16 H 0.125809 3.221750 0.021864

17 C 0.155469 0.293349 1.633519

18 H 0.427659 0.833459 2.543542

19 H 0.149730 -0.767729 1.856927

20 C -1.237878 0.746067 1.191968

21 H -1.350695 1.827708 1.145257

22 H -1.974736 0.333969 1.883230

23 C -3.448727 0.092993 -0.526007

24 H -3.779189 1.086768 -0.216414

25 H -3.657542 -0.015067 -1.592596

26 C -4.047220 -1.037343 0.297789

27 H -3.640238 -1.997561 -0.018527

28 H -5.130828 -1.054481 0.163427

29 H -3.847875 -0.915775 1.364781

**Table S51**. Final Cartesian coordinates (X, Y, Z in Å) for the optimized TNZ molecule (conformer no. 185)

**Number Atom X Y Z**

1 S -1.571325 -0.538086 -0.334868

2 O 2.110493 -1.919493 1.191697

3 O 3.100653 -1.931905 -0.745309

4 O -0.728132 -1.732788 -0.426685

5 O -1.466517 0.537777 -1.328122

6 N 1.951097 2.028003 -0.957439

7 N 1.083948 0.681639 0.599181

8 N 2.419956 -1.394423 0.118934

9 C 2.521858 0.800408 -1.085566

10 H 3.279701 0.575740 -1.817211

11 C 2.008810 -0.053904 -0.139953

12 C 1.086230 1.934126 0.040306

13 C 0.182967 3.035220 0.467715

14 H 0.612357 3.977051 0.130266

15 H 0.051961 3.071682 1.552106

16 H -0.796897 2.920317 -0.005356

17 C 0.202703 0.202743 1.665119

18 H 0.353220 0.823031 2.552080

19 H 0.522663 -0.806506 1.898781

20 C -1.285181 0.214847 1.306582

21 H -1.710432 1.215229 1.262765

22 H -1.828454 -0.379244 2.043593

23 C -3.303535 -1.083145 -0.275929

24 H -3.415784 -1.625364 -1.217375

25 H -3.366786 -1.803112 0.541683

26 C -4.287972 0.071489 -0.155114

27 H -4.177815 0.601803 0.793430

28 H -5.309850 -0.310074 -0.204284

29 H -4.144928 0.782859 -0.968926

**Table S52**. Final Cartesian coordinates (X, Y, Z in Å) for the optimized TNZ molecule (conformer no. 199)

**Number Atom X Y Z**

1 S -1.673661 0.079571 -0.039409

2 O 1.775245 2.098305 1.268956

3 O 2.503600 2.484148 -0.744295

4 O -1.467942 -0.860579 -1.147996

5 O -1.104683 1.430179 -0.090541

6 N 2.207192 -1.604026 -1.246585

7 N 1.262106 -0.610323 0.515963

8 N 2.060025 1.741914 0.122593

9 C 2.484401 -0.273995 -1.304218

10 H 3.086040 0.169935 -2.079502

11 C 1.915993 0.368215 -0.231166

12 C 1.465077 -1.783192 -0.165194

13 C 0.873584 -3.087786 0.233377

14 H -0.154755 -3.160261 -0.133477

15 H 0.875538 -3.235183 1.316225

16 H 1.454688 -3.881191 -0.233705

17 C 0.425611 -0.423015 1.702204

18 H 0.554072 0.609175 2.008550

19 H 0.798532 -1.066621 2.502564

20 C -1.058341 -0.725395 1.480561

21 H -1.628002 -0.324092 2.320709

22 H -1.275213 -1.786258 1.368967

23 C -3.463734 0.225283 0.249138

24 H -3.819384 -0.779867 0.485063

25 H -3.580517 0.873921 1.118825

26 C -4.136223 0.796797 -0.994311

27 H -3.743851 1.788350 -1.223262

28 H -5.210594 0.880418 -0.820686

29 H -3.971207 0.147677 -1.854864

**Table S53**. Final Cartesian coordinates (X, Y, Z in Å) for the optimized TNZ molecule (conformer no. 148)

**Number Atom X Y Z**

1 S -1.673660 -0.079829 -0.039387

2 O 2.503904 -2.483768 -0.744597

3 O 1.775997 -2.098117 1.268868

4 O -1.104358 -1.430291 -0.090782

5 O -1.468297 0.860530 -1.147854

6 N 2.206866 1.604372 -1.246536

7 N 1.262039 0.610375 0.515977

8 N 2.060418 -1.741672 0.122456

9 C 2.484343 0.274408 -1.304247

10 H 3.086045 -0.169393 -2.079557

11 C 1.916078 -0.367976 -0.231225

12 C 1.464707 1.783329 -0.165124

13 C 0.873036 3.087819 0.233561

14 H 1.453396 3.881222 -0.234445

15 H 0.876092 3.235629 1.316356

16 H -0.155696 3.159843 -0.132254

17 C 0.425594 0.422793 1.702236

18 H 0.798507 1.066319 2.502659

19 H 0.554255 -0.609422 2.008422

20 C -1.058394 0.724950 1.480686

21 H -1.275507 1.785798 1.369420

22 H -1.627982 0.323350 2.320750

23 C -3.463650 -0.226003 0.249372

24 H -3.580121 -0.875429 1.118514

25 H -3.819416 0.778873 0.486302

26 C -4.136372 -0.796496 -0.994419

27 H -3.971689 -0.146576 -1.854429

28 H -5.210683 -0.880461 -0.820573

29 H -3.743910 -1.787783 -1.224379

**Table S54**. Final Cartesian coordinates (X, Y, Z in Å) for the optimized TNZ molecule (conformer no. 26)

**Number Atom X Y Z**

1 S 1.904025 -0.442973 -0.146616

2 O -1.035883 -2.261649 -0.960787

3 O -2.025974 -2.615499 0.944765

4 O 2.389894 -1.456016 -1.084287

5 O 0.999242 -0.797759 0.951916

6 N -2.666182 1.467332 0.951451

7 N -1.313352 0.530856 -0.559766

8 N -1.603161 -1.879678 0.064098

9 C -2.655251 0.120092 1.155840

10 H -3.235599 -0.364659 1.922581

11 C -1.830785 -0.482002 0.238820

12 C -1.855134 1.692705 -0.070165

13 C -1.583594 3.040064 -0.644602

14 H -1.786971 3.073593 -1.718956

15 H -0.548581 3.360117 -0.491006

16 H -2.237452 3.751121 -0.143268

17 C -0.296425 0.458560 -1.607785

18 H -0.597243 1.137597 -2.407215

19 H -0.288672 -0.552659 -2.003575

20 C 1.099116 0.869858 -1.129270

21 H 1.062464 1.766499 -0.511530

22 H 1.751487 1.039623 -1.986508

23 C 3.350956 0.390279 0.573397

24 H 3.941169 -0.447947 0.950247

25 H 3.895290 0.836988 -0.261120

26 C 2.990910 1.379464 1.672496

27 H 2.418210 2.226499 1.288426

28 H 3.902897 1.774391 2.125107

29 H 2.400469 0.890090 2.446951

**Table S55**. Final Cartesian coordinates (X, Y, Z in Å) for the optimized TNZ molecule (conformer no. 120)

**Number Atom X Y Z**

1 S 1.903921 -0.443078 0.146472

2 O -1.035230 -2.261599 0.960546

3 O -2.026121 -2.615540 -0.944593

4 O 0.999221 -0.797442 -0.952225

5 O 2.389790 -1.456457 1.083728

6 N -2.666180 1.467302 -0.951396

7 N -1.313375 0.530820 0.559846

8 N -1.603019 -1.879675 -0.064076

9 C -2.655267 0.120082 -1.155779

10 H -3.235624 -0.364693 -1.922496

11 C -1.830808 -0.482042 -0.238760

12 C -1.855072 1.692663 0.070192

13 C -1.583551 3.040055 0.644534

14 H -0.548206 3.359563 0.492042

15 H -1.788221 3.074117 1.718638

16 H -2.236546 3.751194 0.142191

17 C -0.296544 0.458593 1.608018

18 H -0.289125 -0.552479 2.004158

19 H -0.597288 1.138073 2.407092

20 C 1.099131 0.869440 1.129535

21 H 1.751536 1.038867 1.986815

22 H 1.062715 1.766290 0.512079

23 C 3.350857 0.390613 -0.573189

24 H 3.894934 0.837330 0.261490

25 H 3.941332 -0.447439 -0.950008

26 C 2.990801 1.379831 -1.672234

27 H 2.400729 0.890400 -2.446937

28 H 3.902781 1.775141 -2.124539

29 H 2.417704 2.226636 -1.288240

**Table S56**. Final Cartesian coordinates (X, Y, Z in Å) for the optimized TNZ molecule (conformer no. 25)

**Number Atom X Y Z**

1 S -1.799372 -0.390676 -0.162387

2 O 0.973278 -2.084740 1.215974

3 O 2.182311 -2.699125 -0.486440

4 O -2.352932 -1.287732 0.854188

5 O -0.801463 -0.860456 -1.127674

6 N 2.914415 1.341333 -0.954311

7 N 1.366325 0.625917 0.488913

8 N 1.671568 -1.848333 0.228569

9 C 2.895119 -0.020891 -0.979919

10 H 3.551457 -0.608436 -1.599353

11 C 1.951577 -0.488649 -0.100009

12 C 1.992079 1.707535 -0.078273

13 C 1.686844 3.121470 0.277385

14 H 1.771502 3.297795 1.353741

15 H 0.681772 3.423534 -0.032127

16 H 2.406778 3.754803 -0.237386

17 C 0.229188 0.703850 1.404322

18 H 0.446131 1.484427 2.135058

19 H 0.150966 -0.243528 1.929845

20 C -1.091106 1.050105 0.708660

21 H -0.966015 1.844606 -0.027275

22 H -1.830135 1.348740 1.452341

23 C -3.176485 0.316418 -1.114487

24 H -2.741632 1.089096 -1.750891

25 H -3.472258 -0.519254 -1.752749

26 C -4.324210 0.809825 -0.245423

27 H -4.673565 0.014156 0.412994

28 H -5.155795 1.125948 -0.878690

29 H -4.033001 1.664931 0.368585

**Table S57.** Final Cartesian coordinates (X, Y, Z in Å) for the optimized TNZ molecule (conformer no. 121)

**Number Atom X Y Z**

1 S 1.799379 -0.389858 -0.162347

2 O -0.971820 -2.084391 1.216547

3 O -2.179617 -2.700210 -0.486304

4 O 0.801903 -0.860354 -1.127722

5 O 2.353270 -1.286373 0.854542

6 N -2.915698 1.339624 -0.954221

7 N -1.366851 0.625600 0.488851

8 N -1.669962 -1.848864 0.228814

9 C -2.895083 -0.022561 -0.980008

10 H -3.550899 -0.610648 -1.599464

11 C -1.951088 -0.489473 -0.100104

12 C -1.993508 1.706683 -0.078414

13 C -1.689431 3.120901 0.277105

14 H -0.684529 3.423812 -0.032172

15 H -1.774530 3.297348 1.353421

16 H -2.409807 3.753487 -0.237975

17 C -0.229686 0.704471 1.404089

18 H -0.151033 -0.242568 1.930136

19 H -0.446876 1.485438 2.134327

20 C 1.090343 1.050926 0.708047

21 H 1.829340 1.350396 1.451430

22 H 0.964704 1.844888 -0.028378

23 C 3.176305 0.317833 -1.114401

24 H 3.471504 -0.517264 -1.753669

25 H 2.741357 1.091269 -1.749809

26 C 4.324586 0.810048 -0.245315

27 H 4.034067 1.665041 0.369182

28 H 5.156190 1.125872 -0.878713

29 H 4.673497 0.013785 0.412621

**Table S58**. Final Cartesian coordinates (X, Y, Z in Å) for the optimized TNZ molecule (conformer no. 160)

**Number Atom X Y Z**

1 S -1.843116 -0.058909 0.154533

2 O 0.702355 2.082813 1.295489

3 O 1.569493 2.949476 -0.502968

4 O -1.074602 0.623899 -0.891626

5 O -2.427873 0.690799 1.268432

6 N 3.000995 -0.865356 -1.200432

7 N 1.514338 -0.487128 0.423832

8 N 1.314787 2.003686 0.229443

9 C 2.720671 0.467927 -1.171739

10 H 3.181871 1.181756 -1.833138

11 C 1.808038 0.729415 -0.180810

12 C 2.267062 -1.417888 -0.247540

13 C 2.273991 -2.870642 0.082092

14 H 1.314161 -3.352291 -0.127801

15 H 2.513140 -3.048182 1.134772

16 H 3.035697 -3.345737 -0.533154

17 C 0.515147 -0.801117 1.443804

18 H 0.321527 0.098665 2.020707

19 H 0.947385 -1.549279 2.109883

20 C -0.785205 -1.366426 0.863666

21 H -1.374603 -1.830795 1.655199

22 H -0.592166 -2.090425 0.071829

23 C -3.169237 -1.002838 -0.657030

24 H -2.685100 -1.632177 -1.405731

25 H -3.624686 -1.625845 0.115126

26 C -4.171350 -0.036970 -1.280941

27 H -4.619082 0.600555 -0.517818

28 H -4.964915 -0.602654 -1.772543

29 H -3.683889 0.595891 -2.023395

**Table S59.** Final Cartesian coordinates (X, Y, Z in Å) for the optimized TNZ molecule (conformer no. 167)

**Number Atom X Y Z**

1 S 1.843142 -0.059382 0.154587

2 O -0.701066 2.082291 1.295712

3 O -1.568155 2.949892 -0.502327

4 O 2.427482 0.689970 1.268928

5 O 1.075141 0.623748 -0.891702

6 N -3.001816 -0.864023 -1.200377

7 N -1.514504 -0.486985 0.423572

8 N -1.313708 2.003783 0.229754

9 C -2.720726 0.469116 -1.171441

10 H -3.181613 1.183359 -1.832612

11 C -1.807605 0.729839 -0.180781

12 C -2.267937 -1.417161 -0.247797

13 C -2.275839 -2.869914 0.081805

14 H -2.518599 -3.047344 1.133695

15 H -1.315288 -3.351550 -0.124710

16 H -3.035505 -3.345064 -0.535913

17 C -0.515335 -0.801836 1.443350

18 H -0.947788 -1.550319 2.108928

19 H -0.321515 0.097523 2.020860

20 C 0.784841 -1.367070 0.862820

21 H 0.591595 -2.090457 0.070467

22 H 1.374080 -1.832186 1.654033

23 C 3.169569 -1.003196 -0.656698

24 H 3.625305 -1.625612 0.115759

25 H 2.685634 -1.633096 -1.405057

26 C 4.171225 -0.037251 -1.281173

27 H 3.683484 0.594981 -2.023980

28 H 4.965058 -0.602840 -1.772459

29 H 4.618712 0.600918 -0.518438

**Table S60**. Final Cartesian coordinates (X, Y, Z in Å) for the TNZ molecule taken from the crystal structure of TNZ-monoclinic (conformer TNZ-mono-sp); single-point calculations at the B3LYP-GD3BJ/6-311G(d,p) level of theory

**Number Atom X Y Z**

1 S 2.503653 -0.644711 0.071210

2 O -2.282313 2.912374 0.104208

3 O -0.436617 2.046443 -0.612229

4 O 2.679479 -1.585483 -1.007135

5 O 3.258389 -0.850852 1.278386

6 N -3.605763 -0.954206 0.432046

7 N -1.529731 -0.533512 -0.267029

8 N -1.583039 1.944844 -0.181172

9 C -3.426867 0.382127 0.403697

10 C -2.157978 0.670089 -0.012749

11 C -2.449295 -1.484228 0.034643

12 C -2.187328 -2.937191 -0.039375

13 C -0.142246 -0.774889 -0.680547

14 C 0.786335 -0.647813 0.517382

15 C 2.860642 0.972131 -0.557712

16 C 2.761840 2.076285 0.476981

17 H 1.742988 2.160688 0.815722

18 H 3.072573 3.008978 0.037142

19 H 3.400073 1.843181 1.312595

20 H -4.176486 1.116612 0.671078

21 H -1.480187 -3.213580 0.724491

22 H -1.783414 -3.180235 -1.007731

23 H -3.107681 -3.475819 0.111483

24 H 0.143997 -0.051433 -1.445372

25 H -0.055165 -1.774747 -1.108312

26 H 0.599016 -1.477658 1.200427

27 H 0.556163 0.277571 1.047445

28 H 3.869301 0.971115 -0.973510

29 H 2.167993 1.191901 -1.371482

**Table S61**. Final Cartesian coordinates (X, Y, Z in Å) for the molecule 1 taken from the crystal structure of TNZ-triclinic (conformer TNZ-tri-mol1-sp); single-point calculations at the B3LYP-GD3BJ/6-311G(d,p) level of theory

**Number Atom X Y Z**

1 S -1.818039 -0.003750 0.012323

2 O 2.996104 -2.230792 -0.776956

3 O 1.893065 -2.105089 1.076209

4 O -1.145446 -0.169667 -1.241405

5 O -2.216697 1.324081 0.362271

6 N 2.120368 1.770844 -1.155607

7 N 1.249459 0.580079 0.518756

8 N 2.310357 -1.606398 0.033703

9 C 2.552526 0.491259 -1.267606

10 C 2.033633 -0.263091 -0.257032

11 C 1.346688 1.794486 -0.078069

12 C 0.685852 3.024729 0.428295

13 C 0.387670 0.253315 1.660795

14 C -0.786839 -0.652939 1.306823

15 C -3.232841 -1.061739 0.053183

16 C -4.161461 -0.778179 -1.101047

17 H -4.441824 0.261621 -1.089129

18 H -5.043932 -1.388850 -1.010197

19 H -3.661760 -1.004737 -2.027815

20 H 3.211349 0.122598 -2.044092

21 H 0.942089 3.857040 -0.205369

22 H 1.017772 3.223230 1.433460

23 H -0.382203 2.886275 0.424149

24 H 0.000437 1.180589 2.085639

25 H 0.990325 -0.235730 2.427557

26 H -1.395572 -0.806915 2.199021

27 H -0.401206 -1.625107 0.996246

28 H -2.908137 -2.102391 0.009696

29 H -3.767588 -0.914619 0.992695

**Table S62**. Final Cartesian coordinates (X, Y, Z in Å) for the molecule 2 taken from the crystal structure of TNZ-triclinic (conformer TNZ-tri-mol2-sp); single-point calculations at the B3LYP-GD3BJ/6-311G(d,p) level of theory

**Number Atom X Y Z**

1 S -1.955926 -0.029721 0.037907

2 O 3.459782 -1.959688 -0.756247

3 O 2.042784 -2.163234 0.865022

4 O -1.353053 0.367192 -1.199585

5 O -2.555161 1.002409 0.826281

6 N 2.184856 1.913274 -0.966279

7 N 1.256633 0.536737 0.508890

8 N 2.583686 -1.505478 -0.013299

9 C 2.761907 0.706014 -1.098211

10 C 2.214298 -0.163126 -0.211434

11 C 1.281201 1.795607 0.004759

12 C 0.430990 2.910290 0.453710

13 C 0.340799 0.054171 1.555104

14 C -0.745183 -0.871953 1.030036

15 C -3.158415 -1.293293 -0.283740

16 C -4.313994 -0.772415 -1.077748

17 H -4.832431 -0.020864 -0.506501

18 H -4.988576 -1.581148 -1.303182

19 H -3.952777 -0.339805 -1.995517

20 H 3.545648 0.463572 -1.805219

21 H 0.567951 3.753200 -0.202547

22 H 0.703599 3.188038 1.457936

23 H -0.601374 2.604083 0.433943

24 H -0.130053 0.913124 2.035498

25 H 0.919167 -0.474829 2.314005

26 H -1.246365 -1.346188 1.875139

27 H -0.282273 -1.659067 0.433003

28 H -2.684724 -2.109752 -0.830816

29 H -3.523639 -1.691767 0.663947

**Table S63**. Final Cartesian coordinates (X, Y, Z in Å) for the molecule 1 taken from the crystal structure of TNZ-hemihydrate (conformer TNZ-hemi-mol1-sp); single-point calculations at the B3LYP-GD3BJ/6-311G(d,p) level of theory

**Number Atom X Y Z**

1 S -1.810705 -0.005976 0.022577

2 O 2.985060 -2.235391 -0.773775

3 O 1.903647 -2.087600 1.096221

4 O -1.124204 -0.166960 -1.224574

5 O -2.222683 1.319022 0.375195

6 N 2.088692 1.752828 -1.181727

7 N 1.244954 0.587037 0.516837

8 N 2.304955 -1.603464 0.042726

9 C 2.516487 0.474103 -1.293798

10 C 2.015899 -0.261582 -0.261963

11 C 1.329129 1.797648 -0.088987

12 C 0.692244 3.035398 0.414788

13 C 0.387156 0.271602 1.670844

14 C -0.784907 -0.638654 1.324486

15 C -3.217407 -1.075396 0.050121

16 C -4.125846 -0.796127 -1.126870

17 H -4.423447 0.238843 -1.112698

18 H -5.000032 -1.421834 -1.061918

19 H -3.600972 -1.006382 -2.043511

20 H 3.157100 0.094806 -2.080333

21 H 0.900335 3.846848 -0.262098

22 H 1.085827 3.270791 1.389268

23 H -0.372568 2.888945 0.483021

24 H -0.000078 1.202427 2.087853

25 H 0.992917 -0.210043 2.439839

26 H -1.395760 -0.782198 2.216973

27 H -0.397979 -1.614292 1.026661

28 H -2.885849 -2.114208 0.015192

29 H -3.769519 -0.927406 0.979397

**Table S64**. Final Cartesian coordinates (X, Y, Z in Å) for the molecule 2 taken from the crystal structure of TNZ-hemihydrate (conformer TNZ-hemi-mol2-sp); single-point calculations at the B3LYP-GD3BJ/6-311G(d,p) level of theory

**Number Atom X Y Z**

1 S -1.962416 -0.010163 0.078803

2 O 3.419750 -1.982640 -0.750565

3 O 2.047706 -2.145689 0.906060

4 O -1.334134 0.373127 -1.151907

5 O -2.583590 1.026210 0.849909

6 N 2.153825 1.889931 -1.022768

7 N 1.253678 0.549901 0.508533

8 N 2.564953 -1.514777 0.000071

9 C 2.716211 0.672496 -1.145945

10 C 2.184105 -0.172606 -0.226708

11 C 1.276839 1.797303 -0.032522

12 C 0.437659 2.924575 0.414785

13 C 0.354725 0.089944 1.576803

14 C -0.751054 -0.825556 1.088950

15 C -3.141735 -1.293273 -0.239429

16 C -4.193121 -0.868808 -1.225280

17 H -4.733927 -0.024856 -0.831322

18 H -4.875046 -1.684606 -1.396691

19 H -3.723062 -0.591765 -2.153838

20 H 3.477764 0.407868 -1.869063

21 H 0.740892 3.822711 -0.096421

22 H 0.554049 3.059595 1.476930

23 H -0.594671 2.714970 0.190532

24 H -0.094781 0.959870 2.057900

25 H 0.942641 -0.437926 2.329125

26 H -1.251719 -1.263852 1.953537

27 H -0.305887 -1.640183 0.515810

28 H -2.620933 -2.169908 -0.627447

29 H -3.623496 -1.577207 0.697357
